# Biochemical pathways analysis of microarray results: regulation of myogenesis in pigs

**DOI:** 10.1186/1471-213X-7-66

**Published:** 2007-06-13

**Authors:** Marinus FW te Pas, Ina Hulsegge, Albart Coster, Marco H Pool, Henri H Heuven, Luc LG Janss

**Affiliations:** 1 Animal Breeding and Genetics Centre (ABGC), Animal Sciences Group, Wageningen University and Research Centre, Lelystad, The Netherlands; 2 ABGC, Wageningen University, Department of Animal Breeding and Genetics, Wageningen, The Netherlands; 3 ETH Statistical Animal Genetics Group, ETH Zentrum, Zürich, Switzerland

## Abstract

**Background:**

Combining microarray results and biological pathway information will add insight into biological processes. Pathway information is widely available in databases through the internet.

Mammalian muscle formation has been previously studied using microarray technology in pigs because these animals are an interesting animal model for muscle formation due to selection for increased muscle mass. Results indicated regulation of the expression of genes involved in proliferation and differentiation of myoblasts, and energy metabolism. The aim of the present study was to analyse microarrays studying myogenesis in pigs. It was necessary to develop methods to search biochemical pathways databases.

**Results:**

PERL scripts were developed that used the names of the genes on the microarray to search databases. Synonyms of gene names were added to the list by searching the Gene Ontology database. The KEGG database was searched for pathway information using this updated gene list. The KEGG database returned 88 pathways. Most genes were found in a single pathway, but others were found in up to seven pathways. Combining the pathways and the microarray information 21 pathways showed sufficient information content for further analysis. These pathways were related to regulation of several steps in myogenesis and energy metabolism. Pathways regulating myoblast proliferation and muscle fibre formation were described. Furthermore, two networks of pathways describing the formation of the myoblast cytoskeleton and regulation of the energy metabolism during myogenesis were presented.

**Conclusion:**

Combining microarray results and pathways information available through the internet provide biological insight in how the process of porcine myogenesis is regulated.

## Background

Microarray technology can simultaneously measure the differential expression of large numbers of genes in a tissue and thereby identify the genes involved in the regulation of different stages of a process. Typically, microarray experiments produce long lists of genes that are differentially expressed between two different situations. In order to understand the biology of these data it may be relevant to include physiological information of the genes in the study. Many databases such as the Kyoto Encyclopaedia of Genes and Genomes (KEGG) contain information on biological pathways [1]. Combination of microarray data and pathway data may highlight the processes taking place in the cell providing information on the tissue- and process-specific functioning of the genome.

Mammalian myogenesis, the formation of multinucleated muscle fibres from mononucleated precursor cells called myoblasts, is an exclusive prenatal process [2,3]. Muscle fibre formation takes place in two waves, the primary and secondary muscle fibre formation [4]. Each wave consists of proliferation of myoblasts and fusion to form new muscle fibres. Primary muscle fibres are formed de novo. Secondary myofibres are formed using the primary fibres as a template. Muscle fibre numbers may be related to body and muscle hypertrophy, muscle strength and muscle function [5]. Complex genetic regulatory mechanisms underlie spatial and temporal myogenesis. Central to this regulatory process is the MRF (muscle regulatory factors) gene family, four basic helix-loop-helix transcription factors regulating differentiation stage-specific gene expression [6,7]. A network of genes is involved in the regulation of the expression of the MRF genes thereby regulating the progress through the myogenesis process [8-14].

Pig breeding has mainly focused during the past decades on improving growth rate and muscularity [15]. Furthermore, pig breeds differ in muscle traits such as muscularity, muscle fibre type, colour, etc [16,17]. It can be expected that differences in myogenesis are a major underlying mechanism for the observed phenotypes. This makes the pig an attractive species to study mammalian myogenesis.

Using microarray technology we previously reported on the expression of genes known to affect myogenesis in laboratory animals and *in vitro *model systems [18-21]. Using two extreme pig breeds (Duroc and Pietrain) known to differ for muscle fibre characteristics [16,17], we showed differential expression of myogenesis related genes. The results suggest that the heavier muscled Pietrain breed is delayed in myogenesis and expression of the genes during secondary myogenesis is higher than in the less muscled Duroc breed [18,20]. Furthermore, expression of genes of energy metabolism was higher in the heavy muscle breed compared to the less muscled pig breed. We also reported on the expression profiles during muscle development in Duroc foetuses [19-21]. The profiles showed that both myoblast proliferation and differentiation during primary and to a lesser extent during secondary muscle fibre formation are associated with differential expression of many genes known to regulate these processes. Furthermore, different energy metabolism mechanisms (i.e. pathways) seems to be involved in proliferation (high expression of genes related to oxidative phosphorylation energy mechanism) and differentiation (expression of genes related to glycolysis mechanism).

The present study aimed to relate these myogenesis microarray results with known cellular physiological processes accessible through online pathways databases. Knowing these relationships may provide a better understanding of the regulation of mammalian myogenesis. Pathway database information of species with low genomic information is less than species with sequenced genomes. Software packages usually use species-specific gene IDs to find pathway information in databases. However, the concept of comparative genomics enables to use information from related species. Therefore, we have developed and present here software tools that streamline the process of searching for pathways in online databases followed by combining pathway and microarray data. This enabled us to identify relevant pathways from the KEGG database. Combination of the microarray results with physiologic/biochemical pathway information highlighted regulatory mechanisms of porcine myogenesis.

## Results

### Database searches

**Figure 1:**
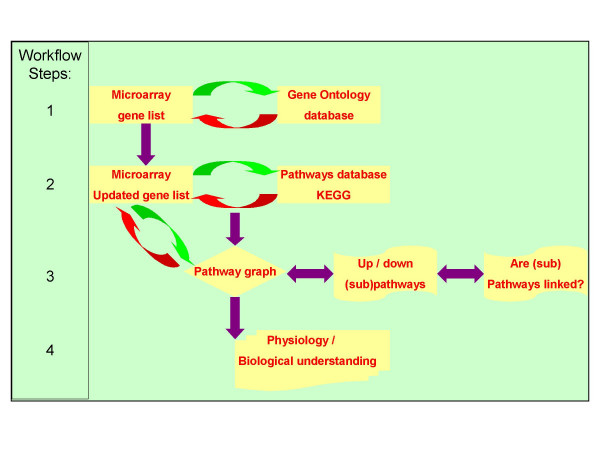
The workflow diagram describing the individual steps taken by the software from microarray data to physiological understanding via pathways analysis. Step 1: A PERL script uses a text file with a list of all genes on the microarray to search the Gene Ontology database for synonyms. These Synonyms are added to the gene list. Step 2 uses this updated gene list to search the KEGG pathway database for pathways in which the genes are involved. If one or more pathways were found for a gene the KEGG database returns a list of pathway names for that gene and a link to the reference pathway for each pathway. Both are added to the file. Step 3 combines the results of the microarray and the pathways. All genes of the pathway represented on the microarray have an expression pattern consisting of the expression in the Longissimus muscle at seven time points during gestation. First all genes of the pathway are considered. Secondly, if more than one biochemical path is specified by the pathway (i.e. called subpathways) the individual subpathways are investigated separately. Thirdly, if KEGG-pathways are linked either because the pathway indicates it or because at least one gene is found in two or more pathways, a network of these pathways is constructed. In step 4 the expression patterns of these pathways and networks were analysed for comparable expression patterns that may indicate common regulatory events linking genes in pathways, subpathways, or networks of pathways creating biological understanding of the physiology of the studied processes.

**Figure 2:**
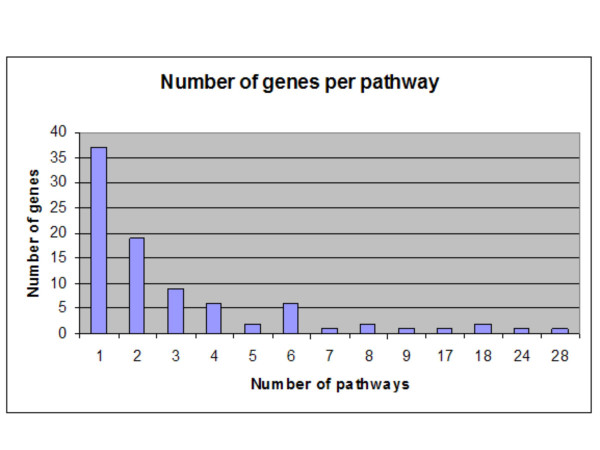
Number of genes per pathway. The KEGG database was screened for pathway information of genes on the microarray with known effect on myogenesis. The Figure shows that the number of genes on the microarray differed per pathway ranging from one gene (28 pathways) up to 37 genes (one pathway).

The list of gene names on the microarray was used to identify pathways in which the genes could be active in the cell (see workflow diagram, Figure 1). The first PERL script collected all synonyms of genes on the array at the GO database. A total of 1524 synonyms of the 509 genes on the microarray were found and added to the list, ranging from zero to a maximum of 56 synonyms in one extreme case (the RPS3 gene). However, it should be noted that not all of these so called synonyms were usable in the pathway database search. Of these 1524 synonyms 22 were contig numbers, 81 were ORF (open reading frame) numbers, and 30 were chromosome localisation numbers. The updated list was used by the second PERL script to search the KEGG database for pathways. From the 509 unique genes on the microarray with known effect on myogenesis and metabolism we found 214 unique genes with known pathway information in the KEGG database. These 214 genes were shown to be active in a total of 88 pathways. The number of genes with microarray information differed per pathway. Figure 2 shows that the number of genes on the microarray differed per pathway. While 37 pathways were represented with only a single gene on the microarray the maximum number of genes on the microarray per pathway was 28. Microarray data from a single gene of a pathway is not sufficient to describe a common regulatory mechanism of a pathway. Furthermore, microarray information of two genes of a pathway not related to a biochemical path is similarly not sufficient to describe a common regulatory mechanism. Therefore, pathways with at least two genes with information on the microarray and localized on one biochemical path were further analysed. Due to this several of these pathways were called not conclusive because of 1 gene entry with microarray data) or low informative (two or more gene entries, but not in a single biochemical path) (Table 1). Twenty-one pathways were further analysed. From the genes on these pathways 5–32 percent was represented on the microarray.

**Table 1:** Number of pathways returned by the KEGG database search using the PERL script

**Pathway statistics**	**N pathways**
Total Number of pathways	88
Not conclusive*	38
low informative**	29
Moderately informative***	12
Highly informative****	9

*: Pathways with two or less genes on the microarray. Two genes are not located on a single biochemical route.

**: Pathways with more than two genes on the microarray, but the genes on the microarray are spread over the pathway

***: Pathways with more than two genes on the microarray and the genes are clustered together on a (sub)pathway

****: Many genes of the pathway have microarray information

**Figure 3:**
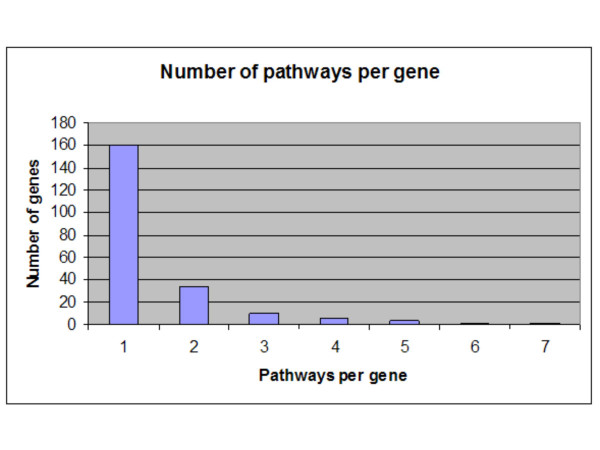
Number of pathways per gene. The KEGG database was screened for pathway information of genes on the microarray with known effect on myogenesis. While most genes were found active in a single pathway, many others proved to be involved in more than one pathway with a maximum of seven pathways for a single gene

We also observed that genes could be active in more than one pathway. Figure 3 show that 160 of the 214 unique genes the KEGG database reported information on one pathway, while one gene was found in seven different pathways. In total we observed information of more than one pathway per gene for 54 genes. This may be a result of the network of pathways that together constitute the processes inside the living cell, but those genes were not covered by the microarray.

### Pathways involved in regulation of porcine myogenesis

Pathways with microarray results for more than one gene were further analysed using previously reported mRNA expression profiles of seven different gestational time points describing the different developmental phases of myogenesis [19-21]. The genes of the microarray were first localised on the pathway. Genes that localise on a biochemical path suggesting biochemical relationships were analysed separately. We named such groups subpathways. From the genes on these subpathways we found 31–75 percent represented on the microarray. The statistical analysis revealed that the expression profiles of genes within a subpathway were more similar than those of genes within pathways (P < 0.001). Only pathways with at least two genes with microarray results in a single subpathway were analysed in detail using the expression profiles provided by the microarray data (see for details the additional files of [19]). Thus, 21 pathways were further analysed (Table 2) for common expression profiles during the myogenesis development. We suggest that similar expression profiles of the genes in a (sub)pathway suggest a common regulatory mechanism, which may be related to the biological trait under investigation. Therefore, (sub)pathways operating in muscle tissue of which the genes show similar expression profiles during the process of myogenesis were analysed for myogenesis developmental stage-specific regulation of expression. The results of all analyses is available in the additional information. Here we present examples of two categories of pathways, without (i.e. Notch and WNT signalling) and with division into subpathways (i.e. Calcium signalling and ATP synthesis), all with indication of common regulation of the expression of the individual genes(Additional files 1,2,3). Each pathway description is accompanied by a figure showing the KEGG pathway with the genes on the microarray encircled (Figures A) and the expression profiles of all the genes of the pathway on the microarray (Figures B). If one or more specific subpathways are indicated the expression profiles of the genes on each subpathway is shown in the following Figure parts (Figures C and further). Note that the genes may be represented by more than one probe, which is indicated.

**Table 2:** (Sub)Pathways further analyzed in detail for common expression profile

**pathway**	**No. of genes on the pathway**	**No genes with microarray data**	**No subpathways**
ATP synthesis	19	6	3
Bile acid biosynthesis	46	7	2
Biosynthesis of steroids	63	3	2
Calcium signalling pathway	48	20	7*
Cell Communication	17	9	N.A.**
Cell cycle	69	4	2*
Fatty acid metabolism	19	6	1
Focal adhesion	57	9	5*
Glutathione metabolism	30	3	1
Glycerolipid metabolism	43	3	1
Glycolysis/Gluconeogenesis	52	16	2
Hedgehog signalling pathway	19	5	1
Jak-STAT signalling pathway	27	3	3***
MAPK signalling pathway	120	26	3
Notch signalling pathway	24	5	1
Oxidative phosphorylation	105	26	3
Proteasome	32	7	5
Purine metabolism	169	6	1
Ribosome	142	19	7****
TGF-beta signalling pathway	55	5	3*****
WNT signalling pathway	70	9	3

*: Genes participate in more than one subpathway

**: Pathway reviews other pathways in a network format

***: Only one subpathway could be analyzed

****: Only four subpathways were analyzed

*****: Only one subpathway was analyzed

#### Notch signalling pathway

**Figure 4:**
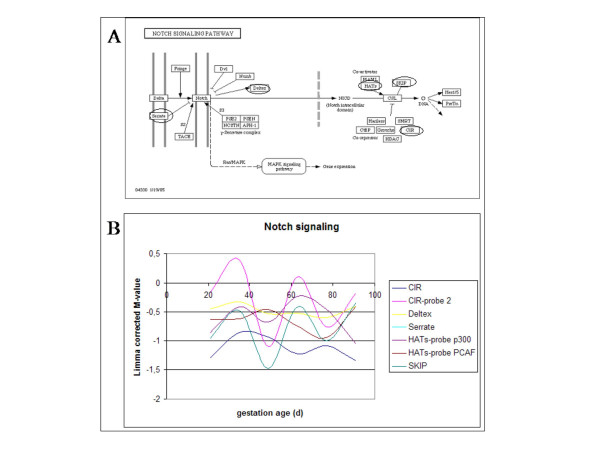
The Notch signalling pathway derived from the KEGG database with the genes on the microarray encircled (A) and their corresponding expression profiles of genes during porcine myogenesis (B). The expression profiles indicate the gestational age at the X-axis and the expression profile as indicated by the differential expression on the microarray on the Y-axis. Since all genes are located in a single biochemical pathway there are no subpathways.

**Figure 5:**
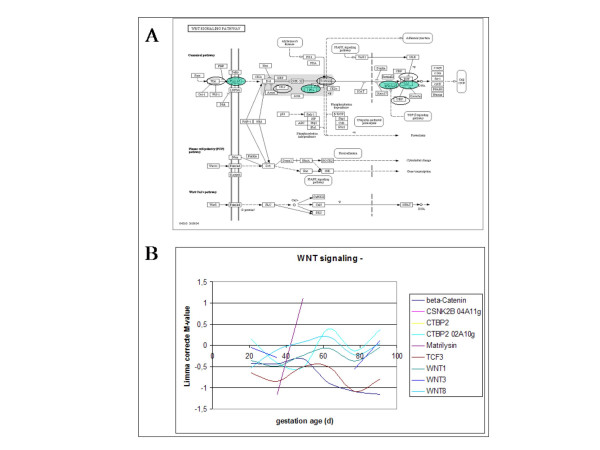
The WNT signalling KEGG pathway consisting of three subpathways with the genes on the microarray encircled (A) and expression profiles of genes during porcine myogenesis (B). The expression profiles indicate the gestational age at the X-axis and the expression profile as indicated by the differential expression on the microarray on the Y-axis. All genes on the microarray are located on the top subpathway directed to influence the cell cycle, i.e. proliferation of myoblasts in myogenesis.

**Figure 6:**
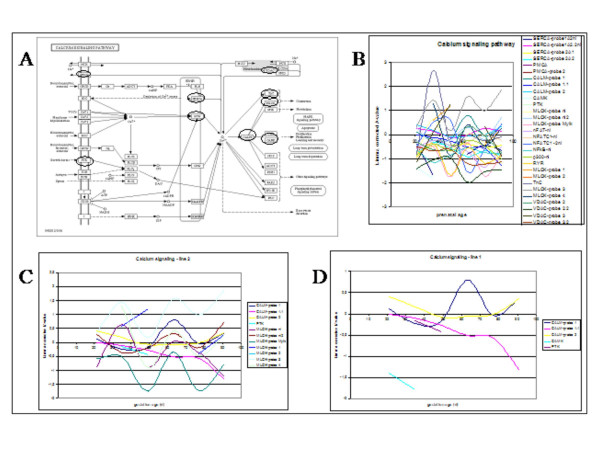
The KEGG derived calcium signalling pathway with the genes on the microarray encircled (A) – the pathway is complex and can be divided in at least five different subpathways all around the calcium ion -, expression profiles of genes during porcine myogenesis (B) including central subpathway with similar expression p;atterns (C) and non-related subpathway (D) gene expression profiles. The expression profiles indicate the gestational age at the X-axis and the expression profile as indicated by the differential expression on the microarray on the Y-axis.

**Figure 7:**
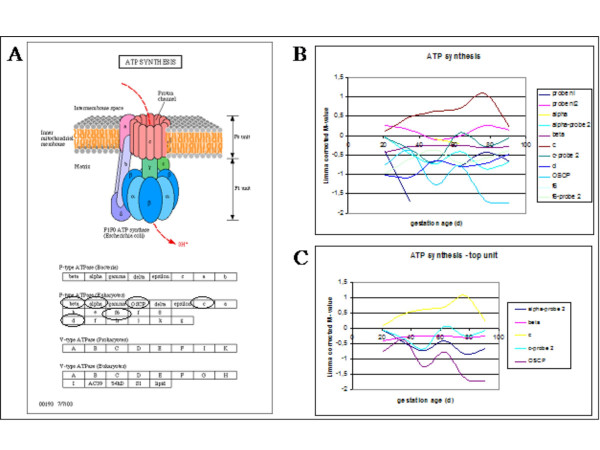
The ATP synthesis pathway derived from the KEGG database indicating how the ATP synthesis complex is composed with the genes on the microarray encircled (A), expression profiles of genes during porcine myogenesis (B) including the top subunit genes (C). The expression profiles indicate the gestational age at the X-axis and the expression profile as indicated by the differential expression on the microarray on the Y-axis.

The Notch family of receptors is activated by their ligands, the Delta gene family, activating a pathway leading to up regulation of myf-5 and down regulation of MyoD expression resulting in blocking of differentiation. As a result the myoblasts are kept in an undifferentiated proliferative state [22,23]. Figure 4 shows the Notch signalling pathway derived from the KEGG database with the genes on the microarray encircled (Figure 4A) and their corresponding expression profiles (Figure 4B). The expression profiles in Figures 4, 5, 6, 7 indicate the gestational age at the X-axis and the expression profile as indicated by the differential expression on the microarray on the Y-axis. Since all genes are located in a single biochemical pathway there are no subpathways. The profile indicates that expression is increased during development twice related to age, and subsequent to developmental stage of myogenesis. The timing of the increases suggests that the expression profile is related to the primary and secondary waves of myofibre formation in pigs. Furthermore, since most genes do show similar expression profiles this may indicate regulation of the expression of all genes in the pathway by a single mechanism. The profiles and regulatory mechanism may indicate a major function of the Notch pathway in porcine myogenesis.

#### WNT signalling pathway

The WNT family of ligands are signalling proteins that regulate developmental processes such as myogenesis and adipogenesis [24]. The WNTs activates the Frizzled receptors during somitogenesis. In the early somite the activation of the pathway leads to down regulation of myf-5 expression. This affects commitment of cells to become myoblasts. Subsequently myoblast proliferation is induced [25]. Figure 5 shows that the KEGG pathway consists of three subpathways (Figure 5A). All genes on the microarray are located on the top subpathway directed to influence the cell cycle, i.e. proliferation of myoblasts in myogenesis. The expression profiles (Figure 5B) give some indication for attenuation of secondary myofibre formation thought the peak expression in the period of 50 – 70 days of gestation.

#### Calcium signalling pathway

Myogenic differentiation involves the fusion of myoblasts to become multinucleated myofibres. Calcium seems to be involved in the IGF-calcineurin-NFATc3 signalling pathway to enhance myogenic differentiation [26]. Figure 6A shows the KEGG derived calcium signalling pathway with the genes on the microarray encircled. The pathway is complex and can be divided in at least five different subpathways all around the calcium ion. The expression profile of all genes of the pathway on the microarray (Figure 6B) is complex, but might indicate that subsets of the genes or subpathways could have common regulation. Therefore subpathways were analysed independently. Figure 6C shows that the genes in the centre subpathway have similar expression pathways suggesting a common regulation, while genes outside this subpathway (Figure 6D) have different expression profiles. Furthermore, these genes do not belong to a single biochemical subpathway and their expression profiles are not related suggesting also different regulatory mechanisms.

#### ATP synthesis

Energy metabolism has been shown to be involved in myogenesis [18-21,27-29]. ATP itself may act via the SWI/SNF complex to modulate chromatin structure to modulate expression of myogenesis differentiation stage-specific genes to block terminal differentiation [30]. Figure 7 shows the ATP synthesis pathway derived from the KEGG database with the genes on the microarray encircled. This pathway differs from the pathways before as this indicates how the ATP synthesis complex is composed. The complex consists of three groups of proteins linked together. The genes on the microarray are divided over the three groups but are mainly focussed on the top group. Figure 7B shows the expression pattern of the genes on our microarray, which is complex and suggest regulation on several different levels. The top line genes show a more similar expression profile (Figure 7C) suggesting common regulation distinct from the genes on the other two lines.

### Network analysis

**Figure 8:**
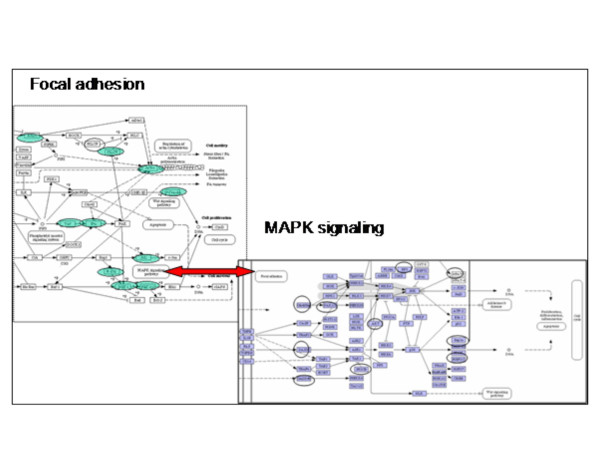
The Figure shows an example of the connection of the KEGG pathways Focal adhesion (partial pathway, Green genes) and MAPK signalling (partial pathway, blue genes). Both biochemical pathways indicate connections to each other (indicated in the boxes) and gene profiles were produced on the microarray.

A network of pathways can be constructed in two ways: (1) KEGG pathways contain boxes indicating possible connections with other pathways, and (2) genes that are active in more than one pathway may indicate direct connections between pathways interacting at the level of the biochemical path. For the latter it is enough that a single gene connects the pathways. KEGG pathway information from boxes indicating possible connections was combined with microarray data to indicate potential interaction between the pathways and used to create potential networks of pathways. For this analysis all pathways returned from the KEGG search were used. If the subpathway reaching to the box of the other pathway was found on the microarray this interaction was added to the network. Figure 8 shows an example of the connection of the KEGG pathways Focal adhesion (partial pathway, Green genes) and MAPK signalling (partial pathway, blue genes). Both biochemical pathways indicate connections to each other (indicated in the boxes) and gene profiles were produced on the microarray. All pathways were screened for such interactions and the networks of interactions between the pathways were created. At least two networks of pathways were found (Additional files 4). One network of pathways describes the formation and maintenance of the muscle cell cytoskeleton including a link to regulation of the contraction mechanism. The other network of pathways describes the interaction between pathways regulating the interactions between the diverse mechanisms involved in energy metabolism.

Genes active in more than one pathway (Figure 3) may strengthen the network. Eight of such genes were found linking pathway components of the network regulating the cytoskeleton formation leading to proliferation and differentiation of myoblasts and contraction of myofibres. Furthermore, 14 genes connected diverse pathways components of the energy metabolism network. Six of them provided a direct connection between the oxidative phosphorylation pathway and ATP synthesis, eight of them connected the Glycolysis/Glucogenesis pathway with several other pathway components of the energy metabolism network.

## Discussion

### Merge microarray data with biological database information

To produce biological meaningful results from the expression patterns of many genes (up to whole genome scans) resulting from microarray experiments we use databases with physiological pathway information. Biochemical pathways such as stored in the KEGG database describe physiological processes taking place in the cell. The physiology of a process may differ between species. Therefore both general (called reference) pathways and species-specific pathways can be searched. Porcine-specific pathways are often not (yet) provided. However, it is noticed that pig physiology closely resembles human physiology [31]. Therefore we used the human pathways information and always compared them with the reference pathways, which were in these experiments always similar. Therefore we decided that we could extrapolate these pathways to be used for the pig.

We developed a PERL script to find all the pathways in the database of the genes on the microarray. The PERL script provides a link to the entire pathway including the genes on the microarray and those not on the microarray. Software tools that can be found on the internet (such as Bioconductor [32], Whole Pathway Scope [33], GOminer [34], and GenMapp [35]) identify genes with their species-specific IDs. As a result only species-specific pathways can be found in pathway databases. However, for many species, including many livestock species like pigs, such data are not available for many pathways resulting in missing information. Therefore, we use the names and synonyms of the genes which find also pathways from other species using the concept of comparative genomics. Therefore, it is possible to analyse data from species with relative low physiological and/or sequence information in the database, giving our software an advantage for those species over the other software tools. However, due to the unlimited finding of pathways from other species it is important to screen for false positive pathways such as photosynthesis metabolism in these analyses.

Searching the KEGG database did not return pathway information for each gene. The main reason for this is that no pathway in the KEGG was found related to these particular genes. For many genes the information on how they act in the cell is still lacking, or not included in the database. Alternatively, a gene may be in the KEGG database with a name not specified in the Gene Ontology database resulting in missing information. This influences the completeness of analyses. Future analyses using other databases may add new data. However, from this point onwards we analyzed the data from the genes with known pathway information and discarded the remaining genes.

The number of pathways per gene differed from one to seven, with almost 75% of the genes with known pathway information belonging to a single pathway. Physiological processes within a cell are not separated but usually linked together in a network. The genes that are active in more than one pathway could be the point of intersection that joins together the pathways into a network.

Due to the relative low number of genes on the microarray which was aimed at studying the myogenesis regulatory processes approximately 60% of the pathways harboured only a low number of genes (i.e. one to three genes). However, most pathways can be divided into subpathways making analysis with only a limited number of genes within the pathway possible. In this study we analysed subpathways harbouring at least two genes on the microarray. Furthermore, statistical analysis showed that expression profiles within subpathways were more similar than within pathways. Biologically, the effect of a pathway on a trait may often be regulated via one of the subpathways of a pathway. Therefore, we argue that where possible pathways analyses always should be analyzed within subpathways.

### Pathways associated with (regulation of) myogenesis

Muscle development is an important characteristic of meat deposition in livestock animals [2,3]. In pigs muscle development has been selected for growth rate and muscularity during the last decades [15]. Therefore, it can be expected that the muscle regulatory mechanism has been under selection pressure that may have influenced the pathways found. Up regulation of positive acting genes and/or down regulation of negative regulatory genes may be more pronounced because of selection. This divergent selection makes the pig an excellent species to study the regulation of myogenesis in mammals.

Expression profiles of genes within a (sub) pathway were compared with the expression profiles of all the genes within the pathway, and with the genes outside the subpathway but in the pathway. Subpathways where the genes showed similar expression profile changes during the gestational period studied were considered to be under a common regulation associated with myogenesis. Therefore, such pathways may highlight the regulatory mechanism underlying muscle development itself. Of these pathways 21 of the 88 pathways had information of at least 2 genes in a subpathway, which we found sufficient for further detailed analyses.

Previously we reported myogenesis differentiation phase-specific regulation of the gene expression using microarrays. Known information regarding the physiology of the genes in the general cell division mechanism and myoblast-specific cell division and cell fusion was used to associate the expression levels of the genes the proliferation and differentiation phase so primary and secondary myofibre formation [19-21]. The results included changes in the expression of energy metabolism genes which were compared to the existing literature on some candidate genes [27-29]. Differences in the timing of these processes between pig breeds were also reported [18]. The present study reports the pathways in which the genes participate aiming to elucidate the underlying regulatory mechanisms.

#### Myogenesis differentiation stage regulating pathways

Myogenesis in mammals proceed in two distinct waves. The Notch signalling pathway described above shows two peak expressions coinciding with the timing of the two waves of myofibre formation [2,3]. The role of the Notch signalling pathway in myogenesis is to keep the cells in an undifferentiated proliferative state by up regulation of proliferation stimulatory genes (Myf-5) and down regulation of differentiation switch genes (MyoD) [22,23]. The two peaks of the expression profile coincide with the two waves of myofibre formation suggesting that myoblasts at least partly regulate proliferation and postponement of terminal differentiation through the Notch pathway. The subpathway of the calcium signalling pathway leading to proliferation of cells showed a similar expression profile, while other subpathways of the same calcium signalling pathway showed different unrelated expression profiles. Contrary to the Notch pathway the calcium signalling pathway enhances the differentiation of myoblasts probably through involvement in the initiation of the fusion of the myoblast cells. Both pathways show peak expression at the switch moment of proliferation to differentiation in primary and secondary myogenesis in pigs [2,3,19-21]. Although speculative, these results may suggest that the balance between the two pathways decide when the cells move from the proliferative state into the differentiation state.

The WNT signalling pathway is suggested to be important for early development in commitment of cells to become myoblasts and start proliferating in somites [25]. The WNT pathway of the KEGG database suggests that most of our genes are on the subpathway leading to cell cycle, so initiate proliferation. Surprisingly not all genes on this subpathway show similar expression profiles. Again there are two peak expressions, but different genes seem to be involved in primary and secondary waves of muscle fibre formation. Some genes may indicate that they may be at a peak expression during somite phase, but in this experiment data on this early time point in gestation are missing. So, although the results are at least suggestive it is uncertain to conclude that the WNT signalling pathway is involved in porcine myogenesis. Furthermore, the Focal adhesion pathway which is suggested to be involved in fusion of myoblasts shows a similar expression profile to WNT signalling (data not shown, see additional data). A similar conclusion as for the WNT signalling pathway can be drawn. Alternatively, it can be suggested that genes within these pathways may be important for different waves of myofibre formation as there are important differences between the two waves of myogenesis [2,3]. During primary muscle fibre formation myofibres are formed *de novo *while during the secondary muscle fibre formation the newly formed myofibres use the primary myofibres as a template to form. This may require different gene expression especially from the WNT and Focal adhesion pathways.

In conclusion these results show that different physiological pathways were found to be involved in the regulation of the proliferation and differentiation steps in myogenesis. These results therefore indicate the way the genome functions to regulate a developmental process.

#### Myogenesis differentiation phase energy metabolism pathways

The ATP synthesis pathway also shows an expression profile similar to the Notch signalling pathway and the calcium signalling pathway. ATP synthesis has been shown to modulate myogenesis by stimulating chromatin structure modulating mechanisms to block differentiation [30]. Previously we reported that we observed ATP metabolism genes (synthesis and associated ATPases) expressions associated with proliferations while glycolysis metabolism related more to differentiation [19]. We argued that the higher energy requiring proliferation may be supported by ATP metabolism while differentiation probably needed reduction of energy support. This is in agreement with the present finding that the ATP synthesis pathway is associated with the proliferative state before terminal differentiation of myoblasts takes place.

Other energy metabolism pathways show more complex expression profiles. The oxidative phosphorylation pathway shows the formation of five complexes. Of these complexes III and V show similar expression profiles suggesting that there may be involvement of the oxidative phosphorylation pathway too, but the other three complexes show different expression profiles making conclusion difficult (data not shown, see additional information). The glycolysis/gluconeogenesis pathway does not show a similar expression profile. Moreover, there seems to be several different profiles linked to one biochemical subpathway. This may either suggest that the regulation of the expression of the genes in this pathway is not associated with myogenesis or that unknown other phases are involved (data not shown, see additional information).

### Networks of pathways

The output of a pathway may be the input of the next pathway. Alternatively, genes may be active in more than one pathway, thereby linking the pathways biochemically. Thus, either directly or indirectly pathways act in a network to fulfil their task in the cell. However, it should be remembered that inside the cell pathways may be separated by compartmentalization or because of activity is separated in time. This may hamper the reported interaction. Networks of pathways should be checked with physiological research.

We found evidence of two networks of pathways, one regulating the formation of the muscle structure, and one showing interactions between the several mechanisms supplying energy to the cells. We reported previously that both mechanisms take a central position during myogenesis [18-21].

Previously we reported on the regulation of the expression of muscle structural genes [19-21]. The expression of muscle structural genes is almost undetectable before myogenesis starts and increases rapidly during the very early stages of myogenesis, i.e. the proliferation phase of the first wave of myogenesis. The cytoskeleton of myoblasts will be different from the contractile apparatus forming the cytoskeleton of the muscle fibres. The network of pathways we observed is focused on regulating the actin cytoskeleton not specifically in muscle fibres. Regulation of proliferation processes but not of differentiation processes was observed. Therefore, we suggest that this network of pathways is especially dedicated to the regulation of the cytoskeleton in myoblasts while related but possibly different pathways regulate the formation of the cytoskeleton during muscle fibre formation. However, the network of pathways also regulates contraction of cells. This could be related to the general cytoskeleton in myoblasts, but may also indicate regulation of the mechanism of muscle cell functioning. The latter may be an indication for regulation of muscle-specific functioning.

We previously reported complex regulation of energy metabolism during porcine myogenesis. Mammalian myogenesis takes place in two waves of proliferation of myoblasts followed by differentiation of preformed myoblasts into multinucleated myofibres or muscle cells [2,3]. During both proliferation phases the genes for ATP synthesis and oxidative phosphorylation reach peak levels while the genes for glycolysis are at a nadir of the expression profile. Contrary to this during differentiation phases the genes for glycolysis show peak levels in the expression profiles and the genes for ATP synthesis and oxidative phosphorylation are at the nadir [19-21]. The network of pathways regulating energy metabolism indeed show the pathways for glycolysis and the citrate cycle in the centre of the network with many connections to other pathways also suggesting a highly regulated network. The network shows that fuel for the energy metabolism may be delivered from several different biochemical routes, i.e. via the anaerobe glycolysis route, and via the oxidative phosphorylation route. Thus, different availability of the biochemical routes during phases in myogenesis may underlie the observed changes in the expression of the energy metabolism mechanisms.

In conclusion: Both networks of pathways provide the first step towards a holistic view of the biochemical reactions that together make up the cell. So, the approach we took has taken us from microarrays to PERL script and bioinformatics to the first step in systems biology. The next steps will be to study interactions of pathway between cells, and between different types of cells towards tissue functioning, organ functioning, etc.

Finally, these results were obtained using the data of a single database. However, there are many more databases accessible through the internet, such as Biocarta [36], Reactome [37], and many more. Adding the data from these databases could highlight more biochemical relationships related to the trait.

## Conclusion

We have analysed data from microarray experiments using bioinformatics tools and biochemical pathways from the KEGG database. The results of combining these sources of data indicate several pathways and subpathways involved in the regulation of myogenesis, especially the crucial moment of the switch between the proliferation and differentiation state that is important for determining the number of muscle fibres [2,3,38,39]. Furthermore, networks of pathways indicating complex regulation of cytoskeleton formation and energy metabolism during myogenesis were found to be active. The expression profiles of genes related to each other in physiological pathways show how the genome functions to regulate prenatal muscle tissue formation. The network of pathways provides a holistic view of the physiology inside the cells during this process. The method is ready to investigate more cellular types in the same way. Future combination of results of several cell or tissue types will highlight the functioning of the genome during tissue formation, a first step towards Systems Biology understanding of life.

## Methods

### Animals and tissue collection

Longissimus muscle tissue samples were collected from the foetuses of pregnant Duroc sows at 14, 21, 35, 49, 63, 77, 91 days of gestation characterising both waves of myogenesis in pigs. RNA was extracted from six foetuses taken from different litters for each gestational age. RNA samples were pooled per gestational age. For more details see te Pas et al. [19].

### Microarrays and analysis

Microarrays were composed of 509 genes known to affect myogenesis and related processes such as fat and energy metabolism. Microarrays were hybridized with Cy3 and Cy5 labelled RNA pools. Each pool consists of RNA isolated from muscle tissue of 6 unrelated foetuses. All experiments comprised pools of two successive gestational ages. Each experiment was done in duplicate and dye swap duplicate. For more details on the microarrays including gene lists and the microarray hybridisations see te Pas et al. [19,20] and Cagnazzo et al. [18]. Differential gene expression was calculated using the Limma package of Bioconductor to correct for microarray-specific hybridization differences. Limma is a statistical method which can be used to identify differentially expressed genes in complex microarray experiments. This method analyzes each gene using a linear regression model. Empirical Bayesian methods are used to provide stable results even when the number of arrays is small. In the results file effects are given for all levels for each factor, compared to the reference. The reference level itself is therefore omitted. We used the measured value of day 14 as the reference value. Differential expression was denoted as the M-value [18,19].

### Database search

The KEGG data base [40] contains general information on biological pathways including gene names and information on species-specific pathways[1] While searching the KEGG database with known pathways we found that genes may be represented with several synonyms that were not all linked to the pathways in the KEGG data base. Therefore, we first linked the microarray data with a local MySQL installation of the Gene Ontology database [41] which contains data of the monthly release of 2006-02-01 to collect all the common names (some of them obsolete) and added these to the file before searching the KEGG database. To automate the searching and retrieving of pathway data from the KEGG database [1] a PERL script was written using the KEGG API [42]. Direct links to each pathway for each gene were added to the file. All database searches were performed with homemade PERL scripts [43]. For additional information see te Pas et al. additional file 5 – software information.

### Analysis combining microarray and database information

Expression profile information about genes (from both regulated and not-regulated genes – see additional data files te Pas et al. [19]) was added to each pathway. Some of the pathways could be divided into several subpathways. When a pathway as defined by the KEGG database was composed of more than one parallel biochemical reaction paths, each path was denoted as a subpathway. Both pathways and subpathway gene expression profiles were analysed. Using the combined information of the KEGG pathways and microarray expression profiles, networks of pathways influencing each other and interacting with each other were identified.

### Statistical analyses

We tested the significance of a common expression pattern within subpathway compared to within pathway with an ANOVA model. Since we were interested in the patterns of the slopes, we calculated the absolute value of the slopes of the slopes. We used these values for fitting and comparing a model with a day*pathway interaction term to a model which included a day*subpathway interaction term. We used the Akaike Information Criterion [44] to contrast the goodness of fit of both models (AIC = -2·log Likelihood + 2·n parameters, and lower AIC values correspond to better models).

## List of abbreviations

KEGG: Kyoto Encyclopaedia of Genes and Genomes

MRF: muscle regulatory factors

PERL: Practical Extraction and Report Language

## Authors' contributions

MFWtP performed the pathways and network of pathways analyses, and wrote the manuscript; IH made the PERL script and performed the database searches; AC did the pathways and subpathways statistical analyses; MHP participated in the LIMMA analyses and supervised the analyses including statistical analyses of the microarrays; HH supervised the statistical analyses of AC; LLGJ performed the LIMMA analyses. All authors read and approved the final version of the manuscript.

## Supplementary Material

Additional File 4 Networks of Pathways and construction. Shows the relationships and interactions between several pathways (called networks), and shows an example of how these networks can be created. Click here for file

Additional File 5 Software information. This file contains: 1. The PERL script to search the Gene Ontology (GO) database using the list of gene names of a microarray, and 2. The source code and PERL script to search the Kyoto Encyclopaedia of Genes and Genomes (KEGG) database for pathways using this updated gene list. Click here for file

Additional File 1 KEGG data and analyses. Shows all data and first analyses of the genes of the microarrays with pathways reported by the KEGG database Click here for file

Additional File 2 Relevant KEGG pathways. Shows the pathways returned by KEGG with the genes with information on the microarrays indicated as circles around the gene name. Click here for file

Additional File 3 Relevant KEGG pathways. Shows the pathways returned by KEGG with the genes with information on the microarrays indicated as circles around the gene name. Gene expression profiles are included Click here for file
