# Supplementary material for: Biochemical pathways analysis of microarray results: regulation of myogenesis in pigs
Source: BMC Dev Biol. 2007 Jun 13;7:66. doi: 10.1186/1471-213X-7-66 (PMC1919358; doi:10.1186/1471-213X-7-66)
Supplement: Additional File 4 — Networks of Pathways and construction. Shows the relationships and interactions between several pathways (called networks), and shows an example of how these networks can be created. [file 1471-213X-7-66-S4.ppt]

## Slide 1
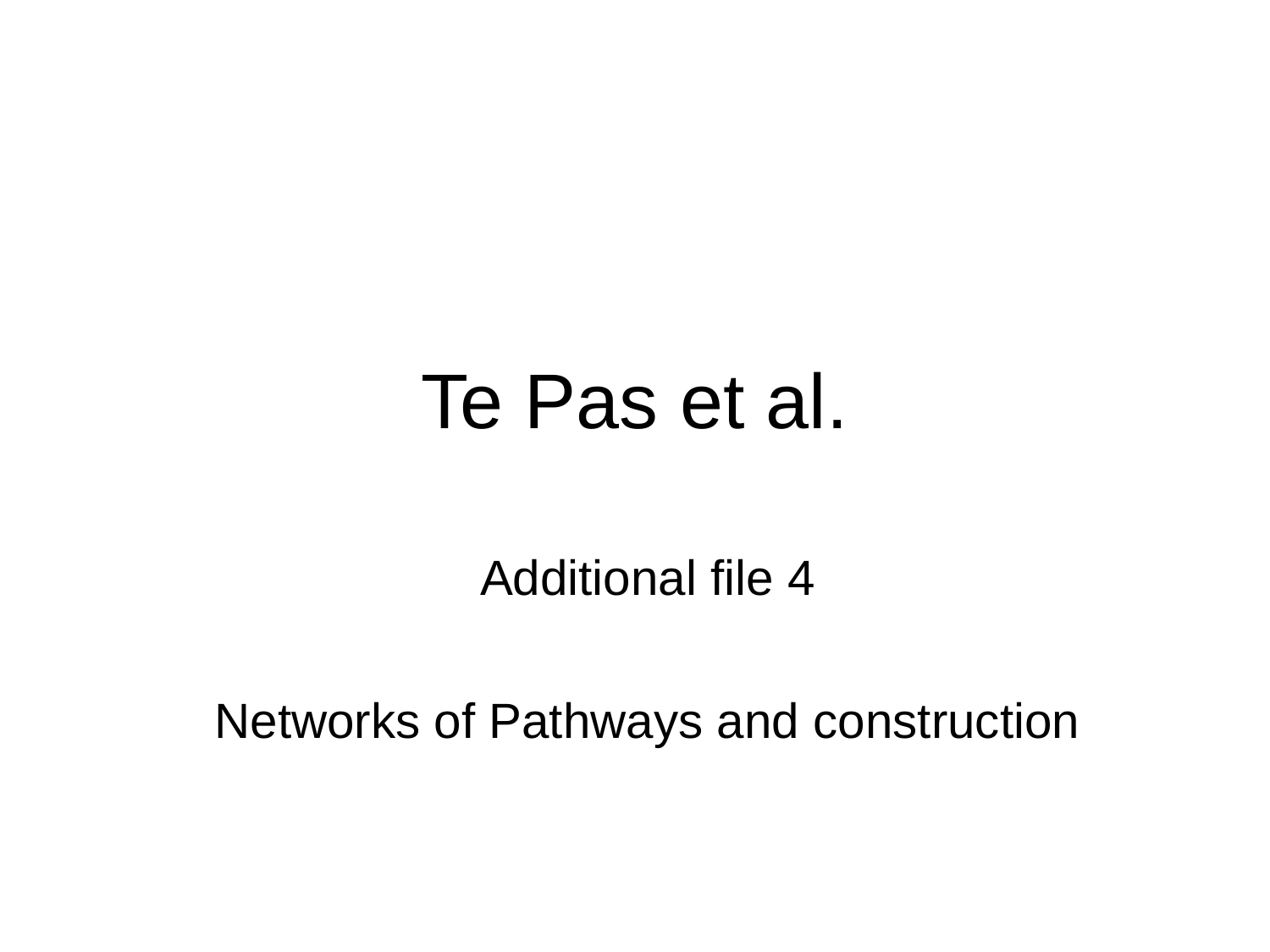

# Te Pas et al.
Additional file 4
Networks of Pathways and construction

## Slide 2
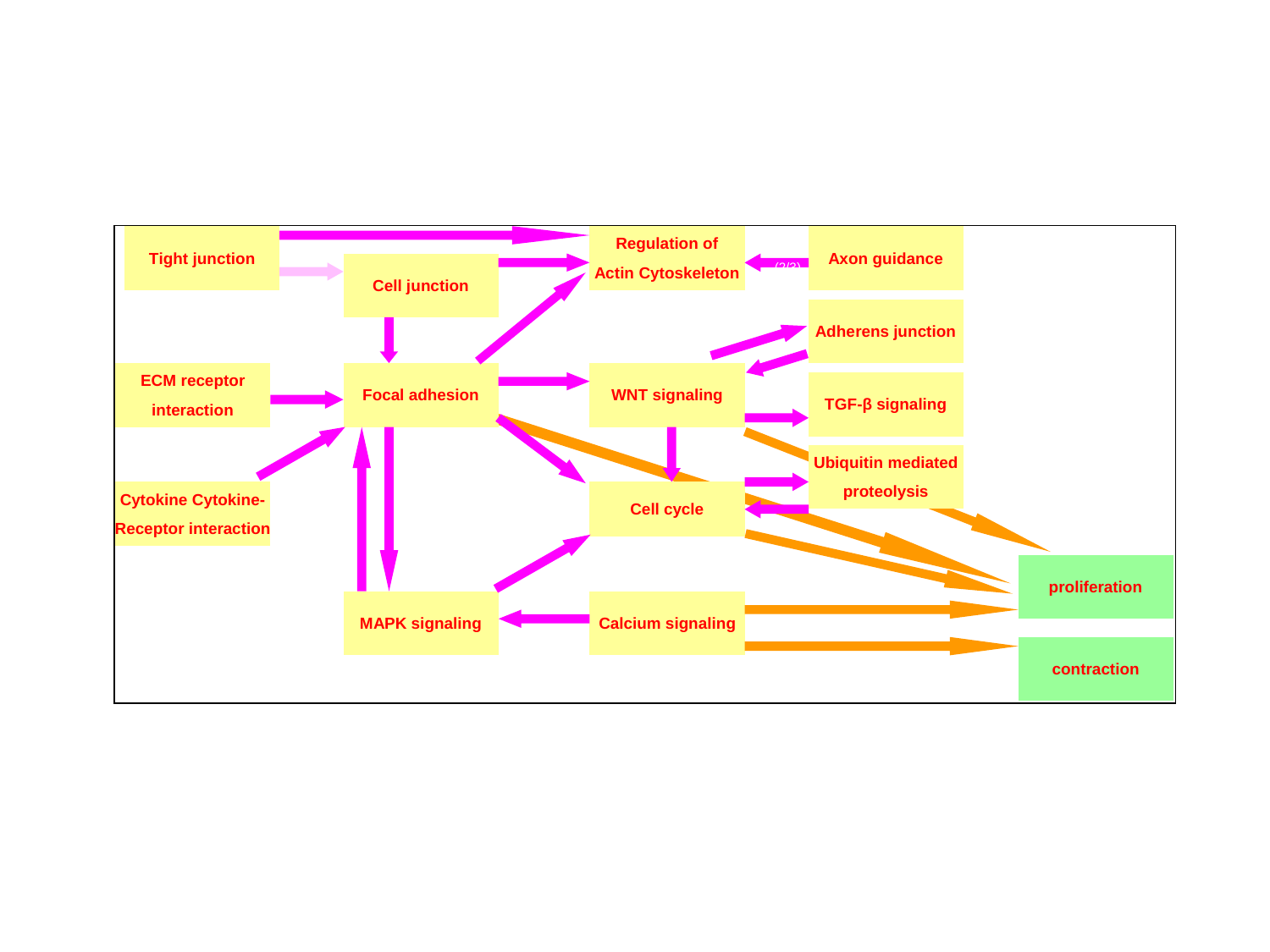

#

## Slide 3
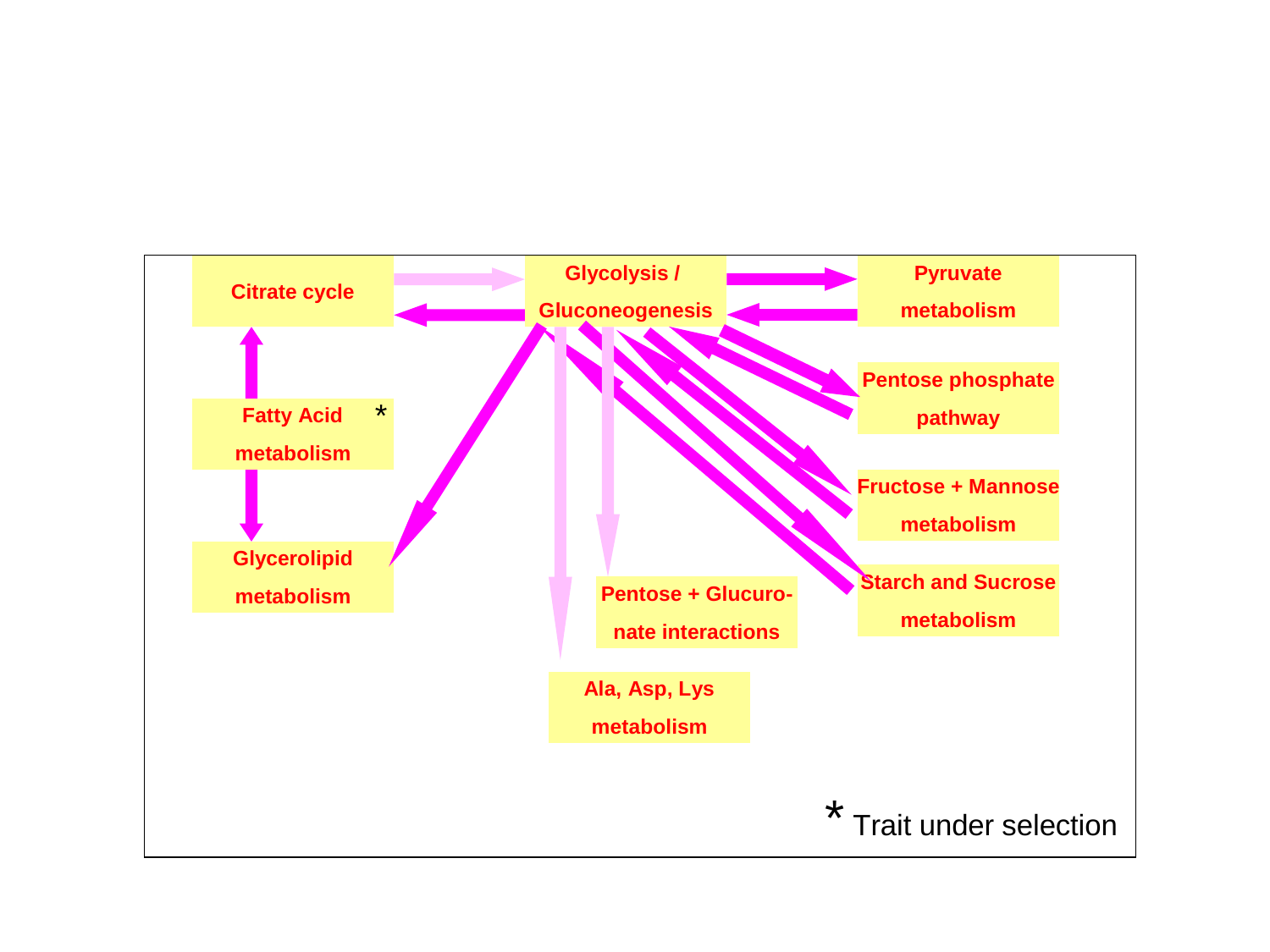

#
*

## Slide 4
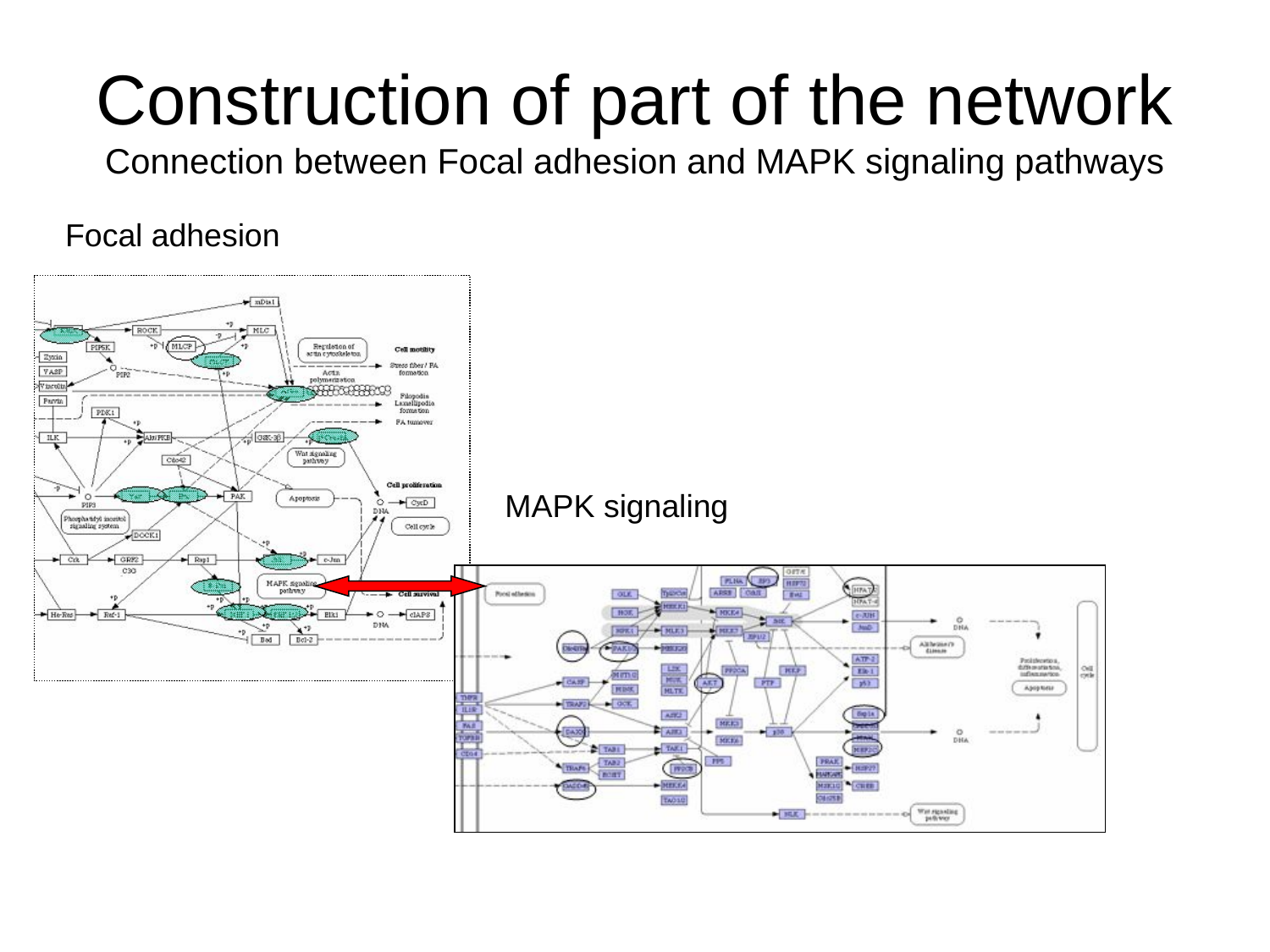

# Construction of part of the network Connection between Focal adhesion and MAPK signaling pathways
Focal adhesion
MAPK signaling

## Slide 5
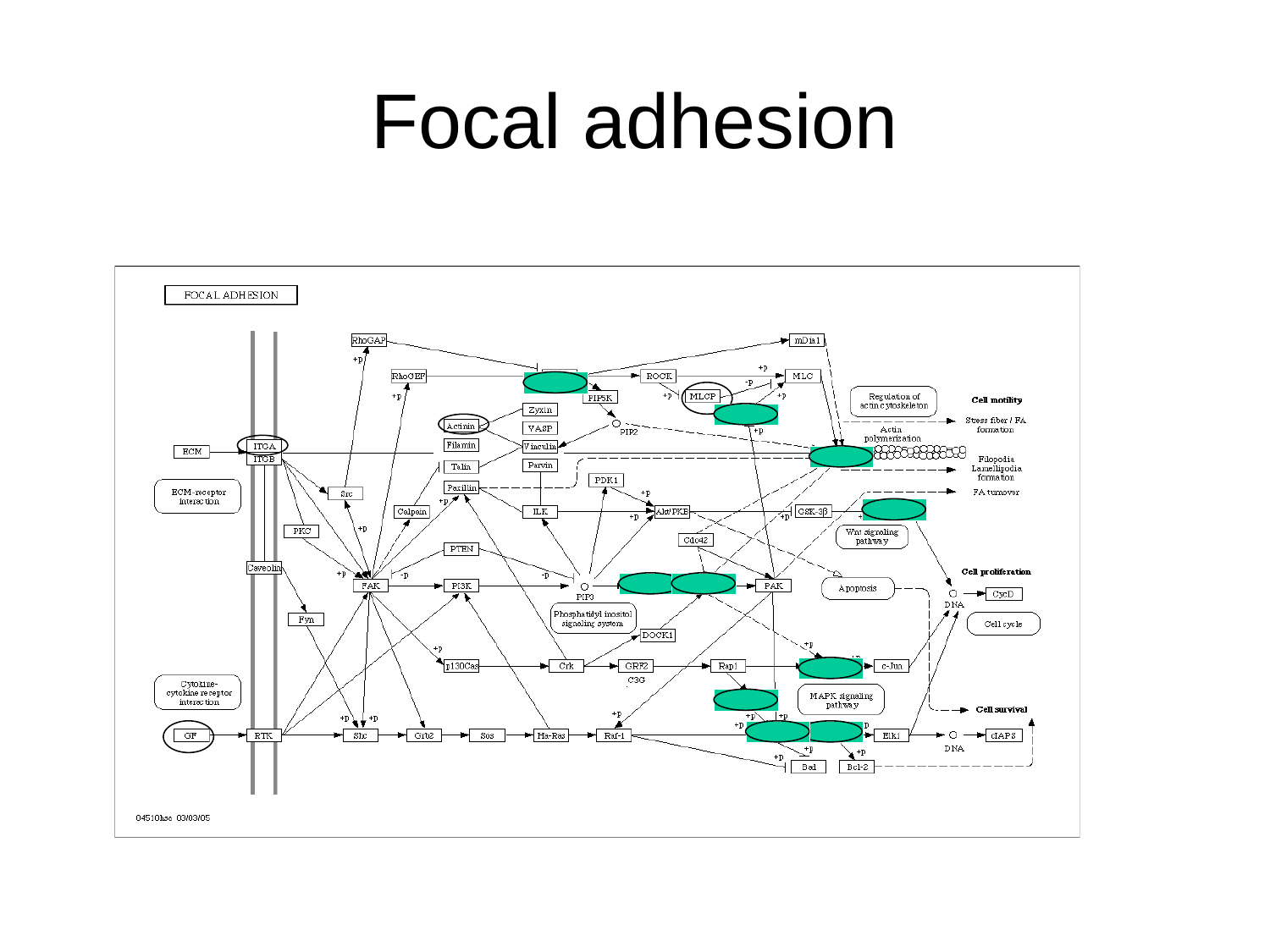

# Focal adhesion

## Slide 6
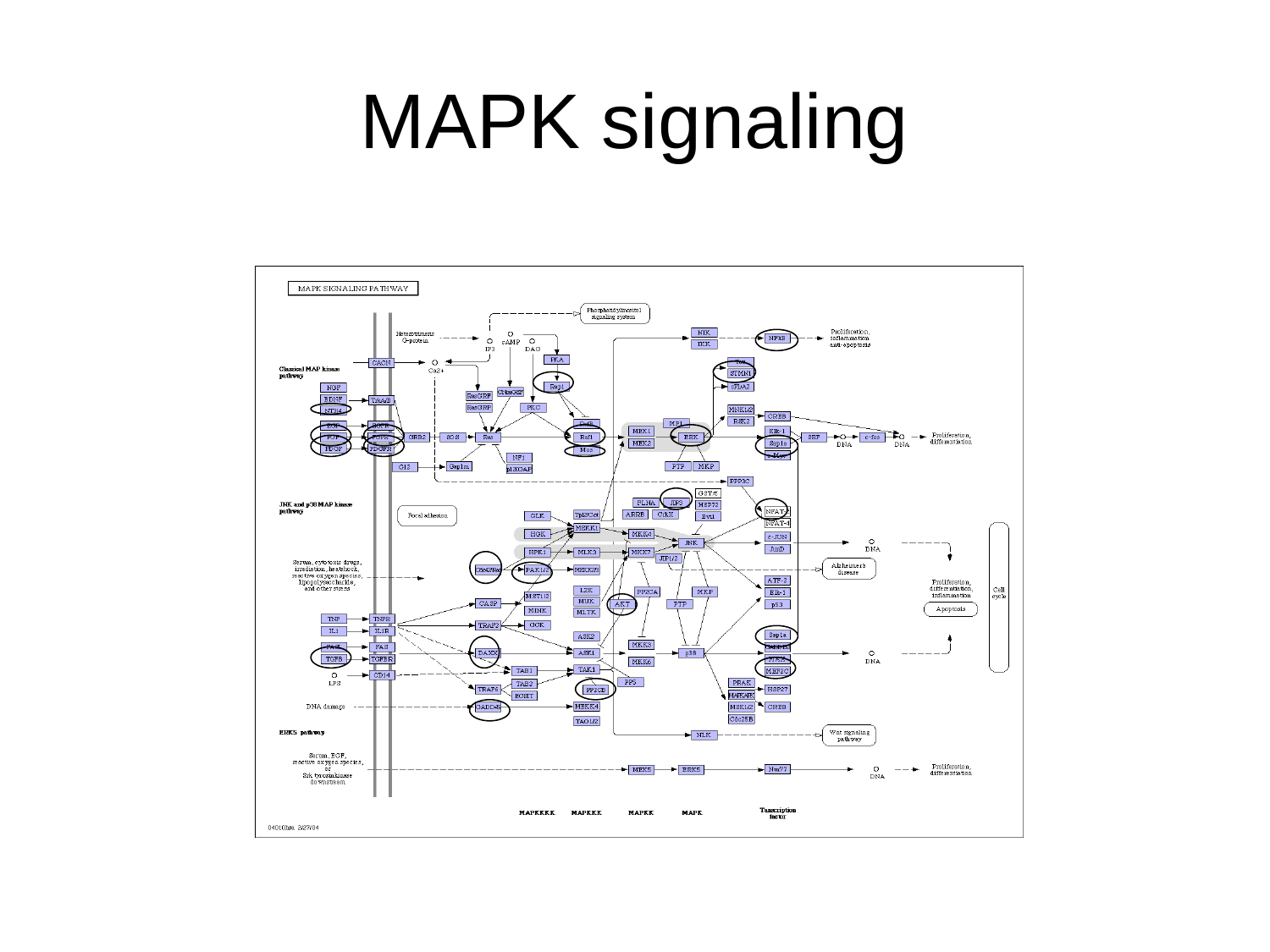

# MAPK signaling
