# Supplementary material for: Biochemical pathways analysis of microarray results: regulation of myogenesis in pigs
Source: BMC Dev Biol. 2007 Jun 13;7:66. doi: 10.1186/1471-213X-7-66 (PMC1919358; doi:10.1186/1471-213X-7-66)
Supplement: Additional File 5 — Software information. This file contains: 1. The PERL script to search the Gene Ontology (GO) database using the list of gene names of a microarray, and 2. The source code and PERL script to search the Kyoto Encyclopaedia of Genes and Genomes (KEGG) database for pathways using this updated gene list. [file 1471-213X-7-66-S5.doc]

**This file contains:**

**1. The PERL script to search the Gene Ontology (GO) database using the list of gene names of a microarray**

**and**

**2. The source code and PERL script to search the Kyoto Encyclopaedia of Genes and Genomes (KEGG) database for pathways using this updated gene list.**

**The Gene Ontology (GO) Database search**

**Introduction**

Microarray results were analysed for pathway information in the KEGG database using gene names of genes on the microarray. Genes may be known with different names. Therefore, the Gene Ontology database was searched before the KEGG database to collect all possible synonyms of gene names.

**Practical information**

The in-house GO database consists of a local MySQL database server that captures GO content,

which contains a download of the monthly release of the GO database. For the present work the release of 2006-02-01 was used.

The installation files of the monthly are available from the GO consortium and MySQL is available from www.mysql.com (both are freely available).

A Perl script is used to collect all the common names (some of them obsolete) of a list of interested genes (see below).

**Perl-script (GO)**

#!/usr/bin/perl -w

use strict;

use DBI; # database independent interface for Perl

use Fcntl qw(:flock); # import LOCK_* constants

my($dbh,$sth,$file,$outfile,$gene,$i);

#Connect to local mysql go (gene ontology) database

$dbh = DBI->connect("dbi:mysql:mygo:localhost",'root','',) ||

die "Unable to connect to contacts Database: $dbh->errstr\n";

$dbh->{RaiseError} = 1;

#name input-file

#$file="c:/eadgene/realdata/gene_name.txt";

$file="c:/eadgene/realdata/gene_name.txt";

#name output-file

$outfile=$file;

$outfile=~s/.txt/_go.txt/;

$outfile=~s/\\/\//g;

#open file with genes names for reading

open(INFILE, "<$file") || die "$! Can't open $file !\n";

flock(INFILE,LOCK_EX) || die "Lock stop $file failed\n"; # exclusive lock, appropriate for reading

#open file for overwring results

open(OUTFILE, ">$outfile") || die "$! Can't open $outfile !\n";

flock(OUTFILE,LOCK_EX) || die "Lock stop $outfile failed\n"; # exclusive lock, appropriate for writing

#write header to output file

print OUTFILE "Gene_Name\tSymbol_GO\tSynonym\n";

#read input-file

RESULT: while(<INFILE>){

chomp $_; # avoid \n on last field

$gene=$_;

print $gene."\n";

#preparing sql select statement

$sth=$dbh->prepare("SELECT gene_product.id, gene_product.symbol,gene_product_synonym.gene_product_id, gene_product_synonym.product_synonym FROM gene_product , gene_product_synonym WHERE gene_product.id = gene_product_synonym.gene_product_id AND (gene_product.symbol='$gene' OR gene_product_synonym.product_synonym='$gene')");

#executing prepared select statement

$sth->execute || die "Unable to execute query: $dbh->errstr\n"; ;

#fetching query result

my $table = $sth->fetchall_arrayref or die "$sth->errstr\n";

#indicate no more data

$sth->finish;

#write results to output-file

if($#{$table}==-1){

print OUTFILE "$gene\n";

next;

}

for $i ( 0 .. $#{$table} ) {

chomp $table->[$i][3];

$table->[$i][1]=~s/\s$//g;

$table->[$i][3]=~s/\s$//g;

next if $table->[$i][1] eq $table->[$i][3];

if($i==0 && lc($table->[$i][1]) eq lc($gene)){

print OUTFILE "$gene\t$table->[$i][1]\t$table->[$i][3]\n";

}elsif($i==0 && lc($table->[$i][3]) eq lc($gene)){

print OUTFILE "$gene\t$table->[$i][3]\t$table->[$i][1]\n";

}elsif($i!=0 && lc($table->[$i][1]) eq lc($gene)){

print OUTFILE "\t$table->[$i][1]\t$table->[$i][3]\n";

}elsif($i!=0 && lc($table->[$i][3]) eq lc($gene)){

print OUTFILE "\t$table->[$i][3]\t$table->[$i][1]\n";

}elsif(($i==0 && lc($table->[$i][1]) ne lc($gene)) && ($i==0 && lc($table->[$i][3]) ne lc($gene))){

print OUTFILE "$gene\n";

}

}

}

close(OUTFILE); # close file for overwring results

close(INFILE); # close file with genes names for reading

#disconnecting from local mysql go (gene ontology) database";

$dbh->disconnect || warn $dbh->errstr;

exit;

**Searching the Kyoto Encyclopaedia of Genes and Genomes (KEGG) database**

**Introduction**

The KEGG database was searched for pathway information of the genes on the microarray using the GO database updated gene list. The search retrieved pathway information from different species including a direct link with the pathway and indication of the position of the gene in the pathway. This pathway information was compared with the reference pathway, which was used for further analysis.

**Practical information**

Pathway_kegg is a in-house web-based tool to search through KEGG pathways. The searching is done by a Perl-written CGI-script running on a in-house web-server. A HTML form is used to upload a text-file containing a list of keywords (gene_names). The tool generates a text file (filename_pathway.txt). The file contains: gene names (keywords), pathway_name and URL.

**Source-code HTML form (KEGG)**

<html>

<head>

<html>

<head>

<title>Pathway_kegg</title>

<link rel="stylesheet" href="style/blauw_01.css" type="text/css">

<style type="text/css">

<!--

.style1 A:link {color:blue}

.style1 A:visited {color:blue}

.style1 A:active {color:blue}

A:hover {color:red}

-->

</style>

<script language="JavaScript" TYPE="text/javascript">

<!--

function cursor()

{

document.form.gene_name_file_up.focus();

}

function validate()

{

document.form.gene_name_file.value=document.form.gene_name_file_up.value;

// Check to make sure the uploaded file is a .txt file

var is_file_ok=document.form.gene_name_file_up.value.indexOf('.txt');

if (is_file_ok==-1 )

{

alert ("\r File type not .txt or no file selected!");

document.form.gene_name_file_up.focus();

document.form.gene_name_file_up.select();

return false;

}

// Check to make sure the uploaded file is on T:

var is_file_ok=document.form.gene_name_file_up.value.indexOf('T:\\');

if ((is_file_ok==-1))

{

alert ("\r File not on T:!");

document.form.gene_name_file_up.focus();

document.form.gene_name_file_up.select();

return false;

}

}

//-->

</script>

</head>

<body link="#ffffff" vlink="#ffffff" alink="#ffffff" background="../img/dnabkgd.gif" onLoad="cursor()">

<h5 align="center"><a href="javascript:void()" onclick="window.open('../pathway_kegg_help.html','','scrollbars=yes,width=700,height=500,left=200,top=150,resizable=yes')"

OnMouseOver="window.status='Help pathway_kegg'; return true"

OnMouseOut="window.status=''; return true"><img align="right" src="../img/help_f2.gif"></a>

Search the KEGG Pathways of genes </h5>

<body>

<form enctype="multipart/form-data"&"text/csv" method="post" action="../cgi-bin/pathway_kegg.pl" name="form" onSubmit="return validate();">

<fieldset>

<dd>This tool search through KEGG pathways to retrieve information of pathways related to genes.</dd></dd><br>

<dd>Pathways can be searched by keywords (gene_names)</dd><br><br>

</fieldset>

<fieldset>

<br>

<table>

<tr><td width="20%">File with keywords: </td>

<td><input type="file" name="gene_name_file_up" size=20></td><td width="55%"><font size="-1">

<input type="hidden" name="gene_name_file">

<span class="style1"><a href="javascript:void()" onclick="window.open('example_pathway_kegg.txt','','scrollbars=no,width=150,height=120,left=200,top=150,resizable=yes')">Here </a></span>is an example file with 6 keywords (gene_names).</i></font></td></tr>

</table>

<br>

</fieldset>

<tr><td><input type="submit" value="Search pathway"></td></tr></table><br>

</body>

</html>

**Perl-script (KEGG)**

#!c:/perl/bin/perl -w

print "Content-type: text/html\n\n";

#===============================================================================

#pathway_kegg.pl,version 0.1

#

# Animal Sciences Group

#

#===============================================================================

#This script used KEGG API to obtain pathway data directly from KEGG's website

#KEGG API is a web service to use the KEGG system from your program via SOAP/WSDL

#For the general information on KEGG API see the following page at GenomeNet:

# <URL:http://www.genome.jp/kegg/soap/>

#KEGG API can access to all the resources stored in KEGG as a batch processing style

#Manual: http://www.genome.jp/kegg/docs/keggapi_manual.html

#Reference: KEGG API: A Web Service Using SOAP/WSDL to Access the KEGG System

#Shuichi Kawashima, Toshiaki Katayama, Yoko Sato, Minoru Kanehisa

#Genome Informatics 14: 673-674 (2003)

#===============================================================================

print <<ENDHTML;

<html>

<head>

<title>Search the KEGG Pathways of genes </title>

<style type="text/css">

<!--

body { font-family: Arial, Helvetica, Sans-serif }

h2,h3,h4 { font-family: Arial, Helvetica, Sans-serif; color: #003366 }

h2 { color: #ffffff; background-color: #336699; border: width: 100%; font-size: 1.46em }

h5 { color: #ffffff; background-color: #003366; border: solid thin #336699; width: 100%; font-size: 1.46em }td { font-family: Arial, Helvetica, Sans-serif; color: #003366 }

A:hover { color:#ff0000; text-decoration: underline }

.style1 A:link {color:#003366}

.style1 A:visited {color:#003366}

.style1 A:active {color:#003366}

.style1 A:hover {color:red}

-->

</style>

</head>

<body link="#ffffff" vlink="#ffffff" alink="#ffffff" background="/img/dnabkgd.gif">

<h5 align="center"> Search the KEGG Pathways of genes </h5>

<table align="center"><tr><td>

<div style="font-size:8pt;padding:2px;border:solid black 1px">

<span id="progress1">&nbsp; &nbsp;</span>

<span id="progress2">&nbsp; &nbsp;</span>

<span id="progress3">&nbsp; &nbsp;</span>

<span id="progress4">&nbsp; &nbsp;</span>

<span id="progress5">&nbsp; &nbsp;</span>

<span id="progress6">&nbsp; &nbsp;</span>

<span id="progress7">&nbsp; &nbsp;</span>

<span id="progress8">&nbsp; &nbsp;</span>

<span id="progress9">&nbsp; &nbsp;</span>

<span id="progress10">&nbsp; &nbsp;</span>

</div>

</td></tr></table>

<script language="javascript">

var progressEnd = 10; // set to number of progress <span>'s.

var progressColor = '#003366'; // set to progress bar color

var progressInterval = 1200; // set to time between updates (milli-seconds)

var progressAt = progressEnd;

var progressTimer;

function progress_clear() {

for (var i = 1; i <= progressEnd; i++) document.getElementById('progress'+i).style.backgroundColor = 'transparent';

progressAt = 0;

}

function progress_update() {

progressAt++;

if (progressAt > progressEnd) progress_clear();

else document.getElementById('progress'+progressAt).style.backgroundColor = progressColor;

progressTimer = setTimeout('progress_update()',progressInterval);

}

function progress_stop() {

clearTimeout(progressTimer);

for (var i = 1; i <= progressEnd; i++) document.getElementById('progress'+i).style.backgroundColor = '#003366';

progressAt = 0;

}

</script>

ENDHTML

use strict;

use LWP::Simple;

use SOAP::Lite;

use Fcntl qw(:flock);

use CGI::Carp qw(fatalsToBrowser);

use CGI;

# start progress bar

print <<ENDHTML;

<script language="javascript">

progress_update(); // start progress bar

</script>

ENDHTML

#system() needed to start progress bar and alert text

system();

# disable file uploads

$CGI::DISABLE_UPLOADS = 1;

my $query = new CGI;

my ($wsdl,$serv,$find,@ids_def,@ids,$i,$url,$url_new,@text,$catalog,@matches,$j);

my ($pathways,$file,$outfile,$gene,$data,$link,$n);

# Read input variables form HTML

# retrieve the upload file name. Note that it is retrieved with

# the same name here ('gene_name_file') that it has in the HTML.

unless ($data = $query->param('gene_name_file'))

{ &showError('No file name specified.'); }

# check to make sure the uploaded file is safe to keep

# by checking its type.

# first retrieve the file type .

# if it is not '.txt' or not on T:, stop and complain.

unless ($data =~/.txt/i)

{

&showError("File type of $data not .txt.<BR>UPLOAD ABORTED.");

}

unless ($data =~ /T:\\/i)

{&showError("$data not on T:")}

$outfile=$data;

$outfile=~s/.txt/_pathways.txt/;

$outfile=~s/\\/\//g;

$n=0;

#URL wsdl

$wsdl = 'http://soap.genome.jp/KEGG.wsdl';

$serv = SOAP::Lite -> service($wsdl);

#open file with keywords

#$file="c:/pathway/example_pathway_kegg.txt";

open(INFILE, "<$data") || die "$! Can't open $data !\n";

flock(INFILE,LOCK_EX) || die "Lock stop $file failed\n";

#open file for results

open(OUTFILE, ">$outfile") || die "$! Can't open $outfile !\n";

flock(OUTFILE,LOCK_EX) || die "Lock stop $outfile failed\n";

RESULT: while(<INFILE>){

chomp $_;

$gene=$_;

$pathways="";

#searching entries by keywords; returns IDs and definitions of entries from KEGG

$find = $serv -> bfind("kegg W:\"$_\"");

if (length($find)==0){

print OUTFILE $gene."\n";

next;

}else{

@ids_def = split(/\n/, $find);

if($#ids_def > 100){

print OUTFILE "$gene\tNUMBER OF HITS FOUND > 100 !!!!\n";

next;

}

$url="http://www.genome.jp/dbget-bin/www_bget?";

#pathway KEGG

#The role of the gene product in a pathway context is shown here by

#linking to the KEGG pathway maps that contain this entry.

#Note that the rectangular object of this gene product is marked red in the pathway.

for($i=0;$i<=$#ids_def;$i++){

@matches="";

@ids = split(/ /, $ids_def[$i]);

#complete URL DBget result

$url_new="$url"."$ids[0]";

#open URL DBget result to read the pathways

$catalog = get($url_new);

@text=split(/\n/,join('',$catalog));

$_=$catalog;

if(/\<code\>PATH:/){

@matches=grep/\<code\>PATH:/,@text;

for($j=0;$j<=$#matches;$j++){

$_=$matches[$j];

/(dbget-bin.*)/;

$_=$&;

/\"\>/;

$link="http://www.genome.jp/$`";

$_=$';

/&nbsp;&nbsp;/;

$_=$';

/\</;

if($n==0){

$pathways=$`;

print OUTFILE "$gene\t$`\t$link\n";

}else{

print OUTFILE "\t$`\t$link\n" unless($pathways=~/\Q$`\E/);

$pathways=$pathways.";".$` unless($pathways=~/\Q$`\E/);

}

$n++;

}

}

}

}

$n=0;

if(length($pathways)==0){

print OUTFILE "$gene\n";

}

}

close(OUTFILE);

close(INFILE);

print "<h3 valign=\"top\"><font color=\"#003366\">Search pathways_kegg completed</h3><br>";

print "<b><h3>Results</h3></b>";

print "<span class=\"style1\"><a href=\"javascript:void(0)\" onclick=\"window.open('$outfile','',

'scrollbars=yes,width=700,height=500,left=200,top=150,resizable=yes')\">result search payhway kegg</a></span>";

print "<td><div style=position:absolute; top:0;>

<font color=\"#003366\"><a href=\"javascript:void(0)\" onclick=\"window.open('../not_available.html','',

'scrollbars=yes,width=700,height=500,left=200,top=150,resizable=yes')\"

OnMouseOver=\"window.status=\'Help Search pathway_kegg\'; return true\"

OnMouseOut=\"window.status=\'\'; return true\"><img align=\"center\" src=\"/img/help_f2.gif\"></a></div>";

# stop progress bar

print <<ENDHTML;

<script language="javascript">

progress_stop();

</script>

ENDHTML

#function errors

sub showError

{

# stop progress bar

print <<ENDHTML;

<script language="javascript">

progress_stop();

</script>

ENDHTML

my @error = @_;

print "<center><font color=\"red\">ERROR - @error</font><BR>\n";

exit;

}
