# Supplementary material for: Biochemical pathways analysis of microarray results: regulation of myogenesis in pigs
Source: BMC Dev Biol. 2007 Jun 13;7:66. doi: 10.1186/1471-213X-7-66 (PMC1919358; doi:10.1186/1471-213X-7-66)
Supplement: Additional File 2 — Relevant KEGG pathways. Shows the pathways returned by KEGG with the genes with information on the microarrays indicated as circles around the gene name. [file 1471-213X-7-66-S2.ppt]

## Slide 1
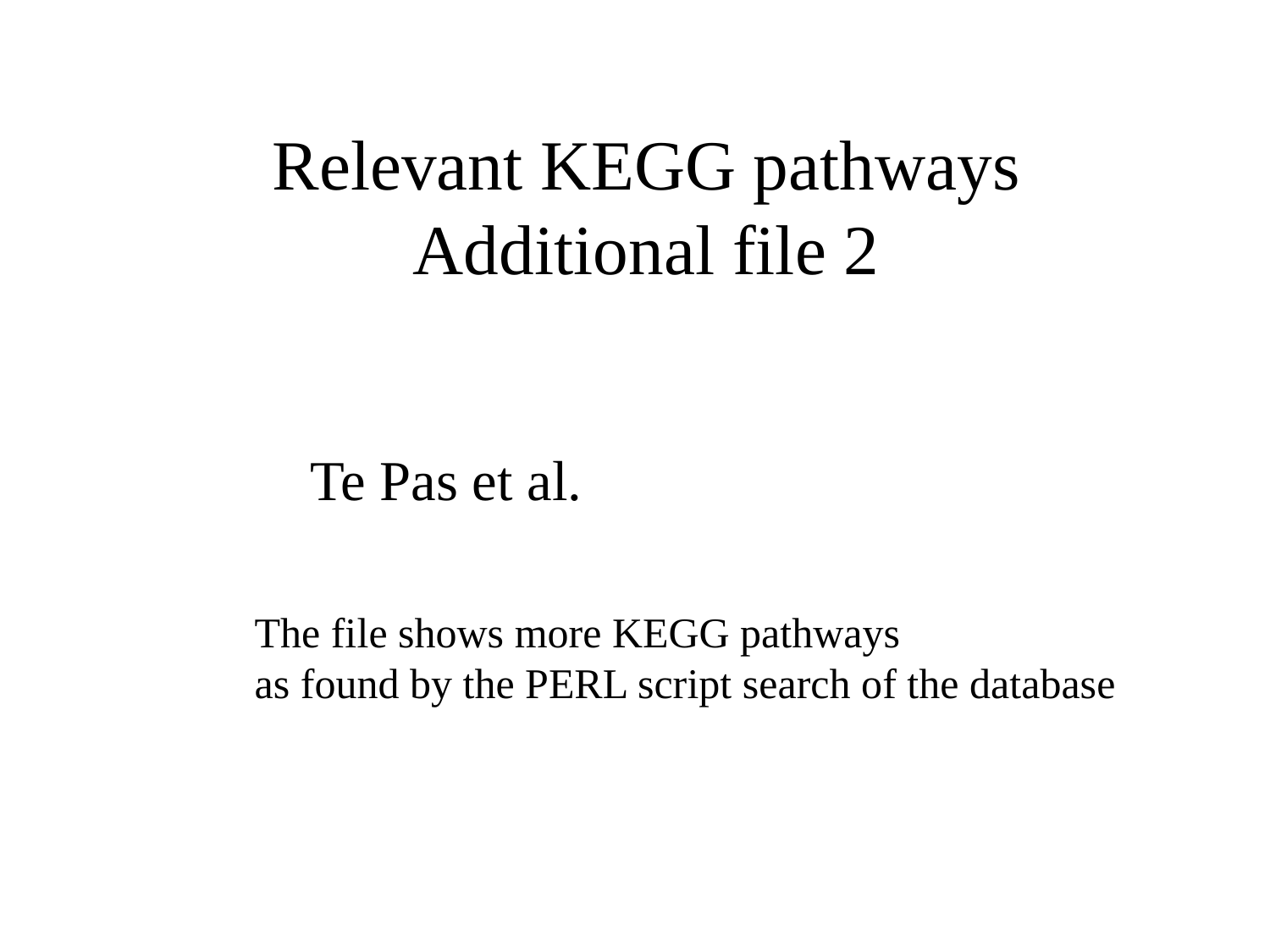

# Relevant KEGG pathways Additional file 2
Te Pas et al.
The file shows more KEGG pathways
as found by the PERL script search of the database

## Slide 2
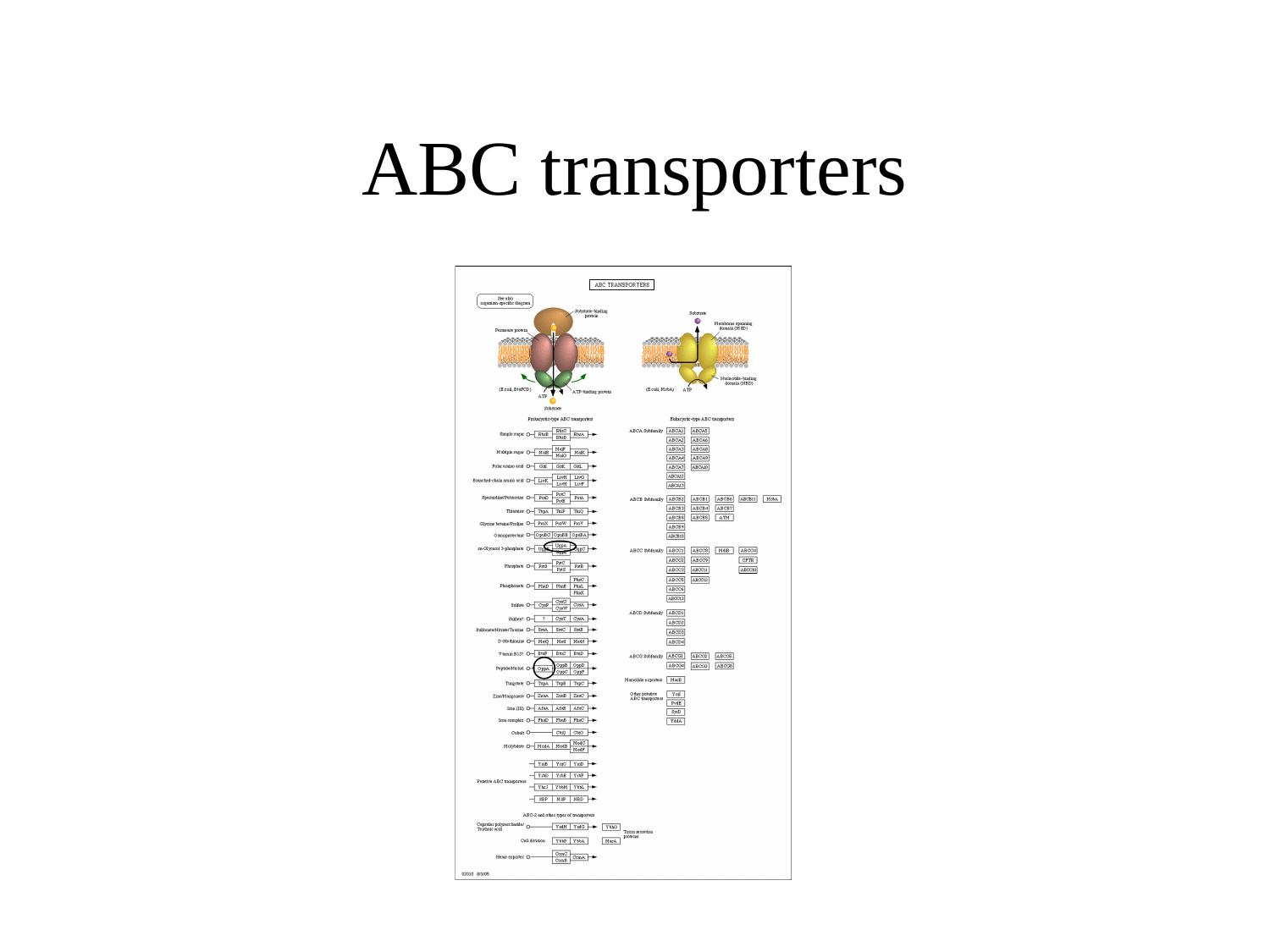

# ABC transporters

## Slide 3
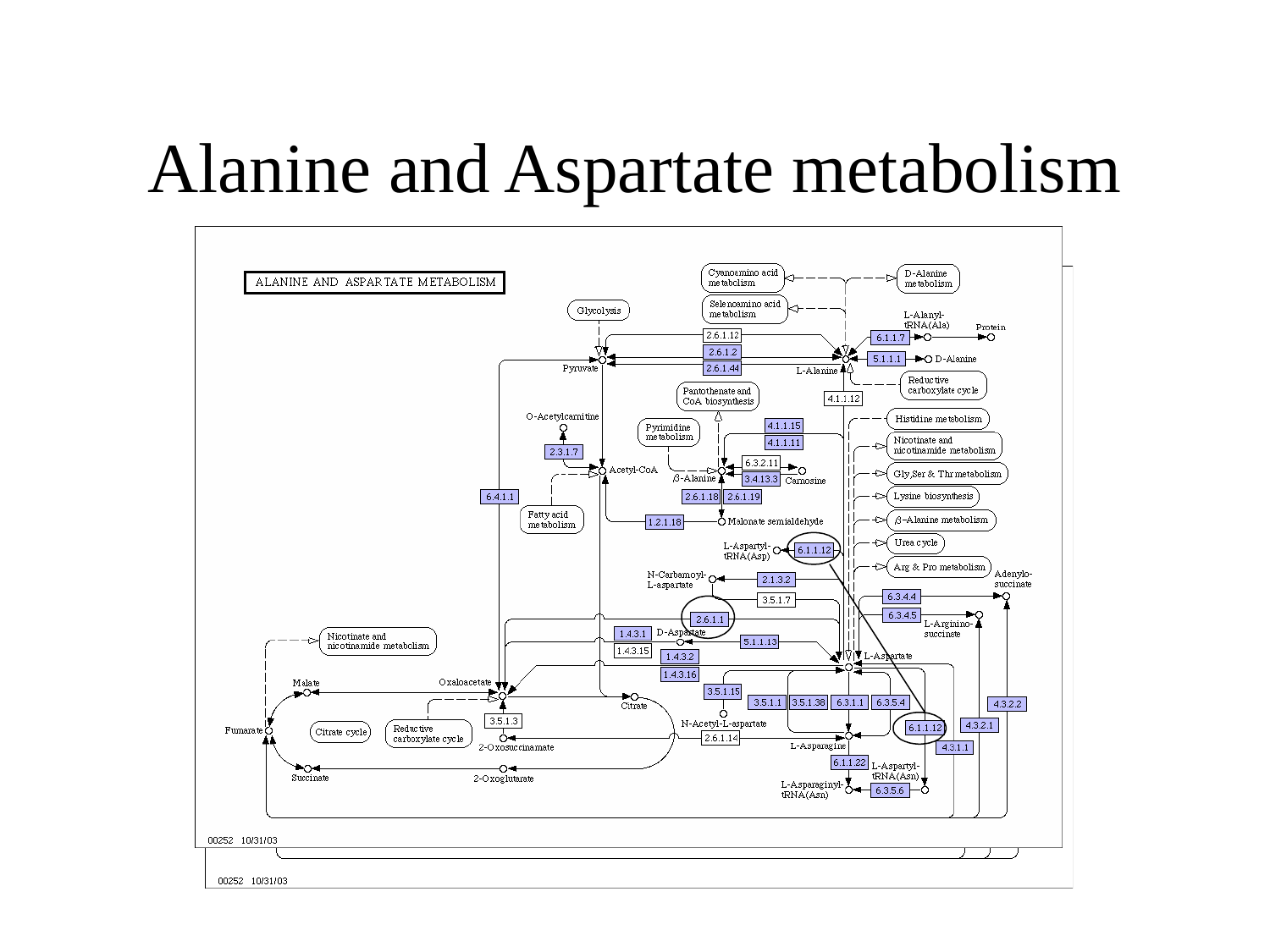

# Alanine and Aspartate metabolism

## Slide 4
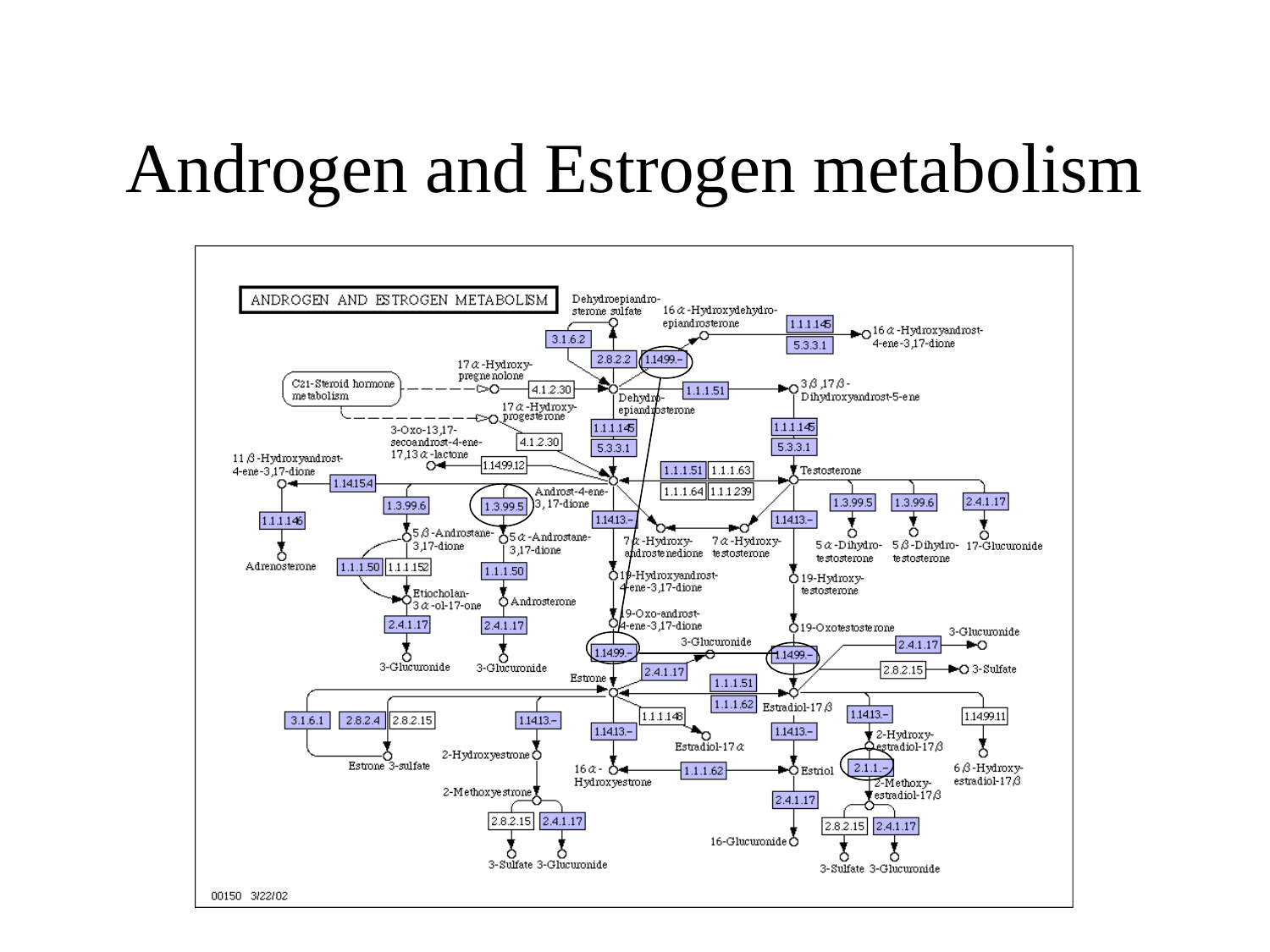

# Androgen and Estrogen metabolism

## Slide 5
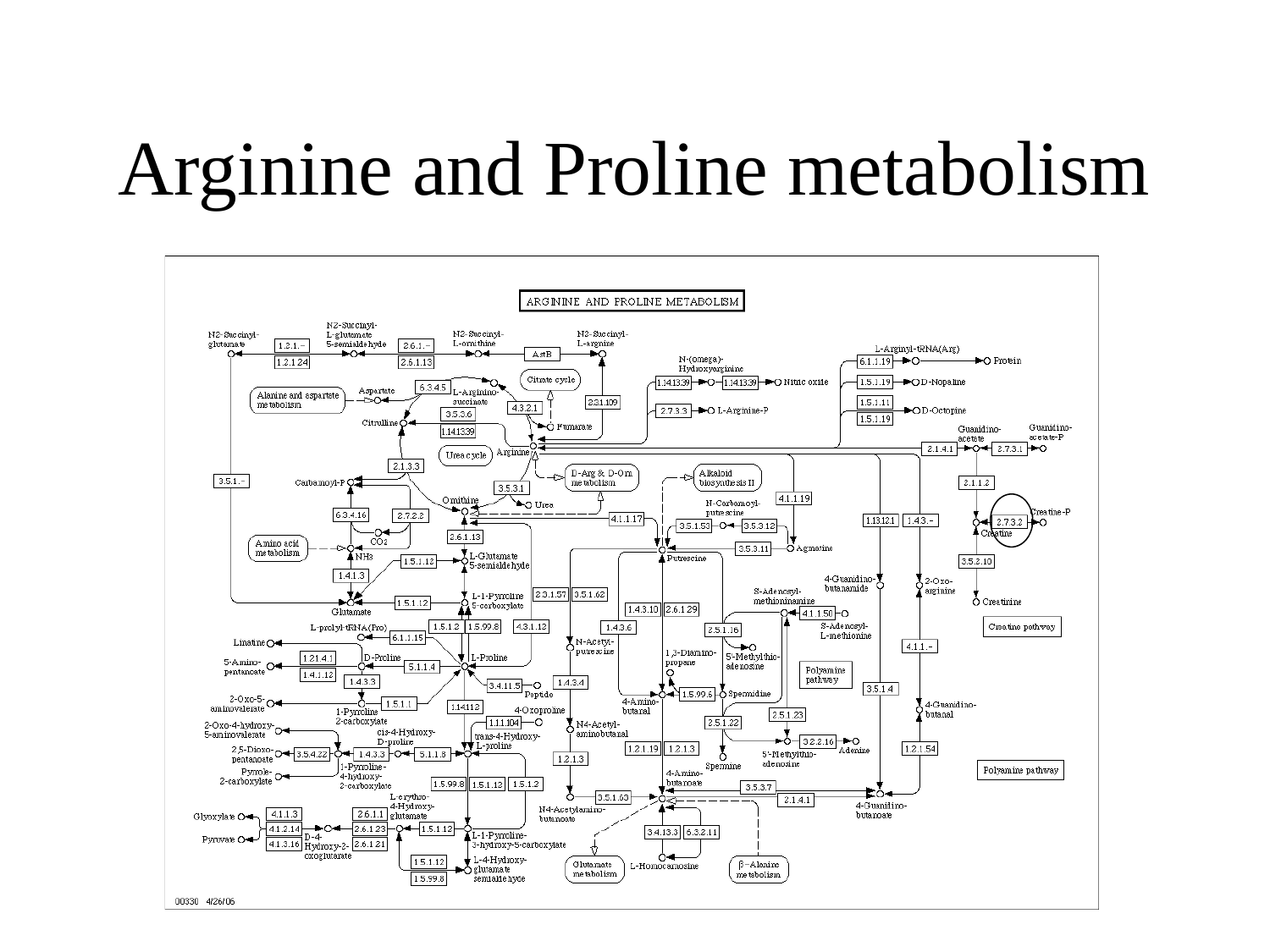

# Arginine and Proline metabolism

## Slide 6
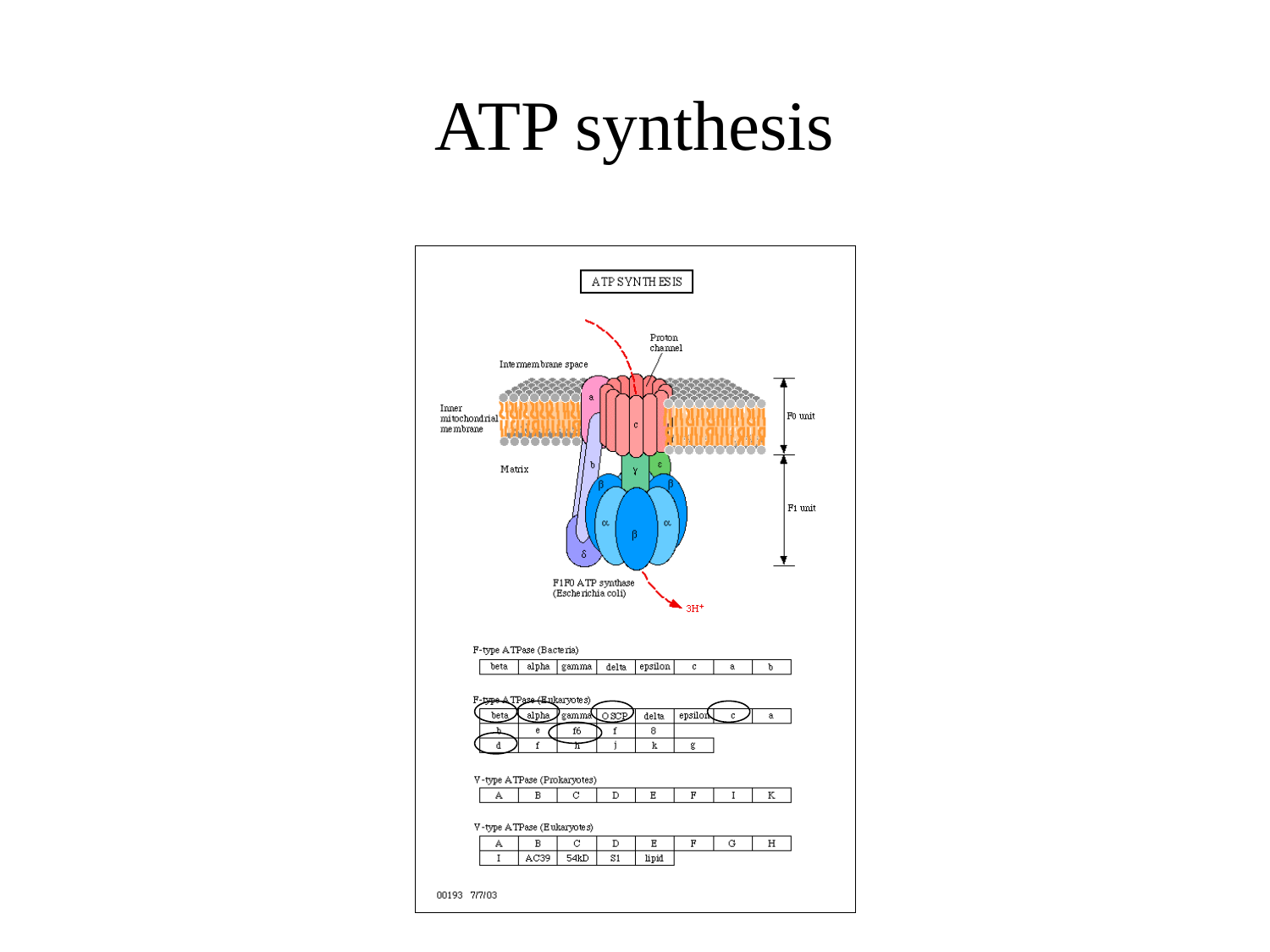

# ATP synthesis

## Slide 7
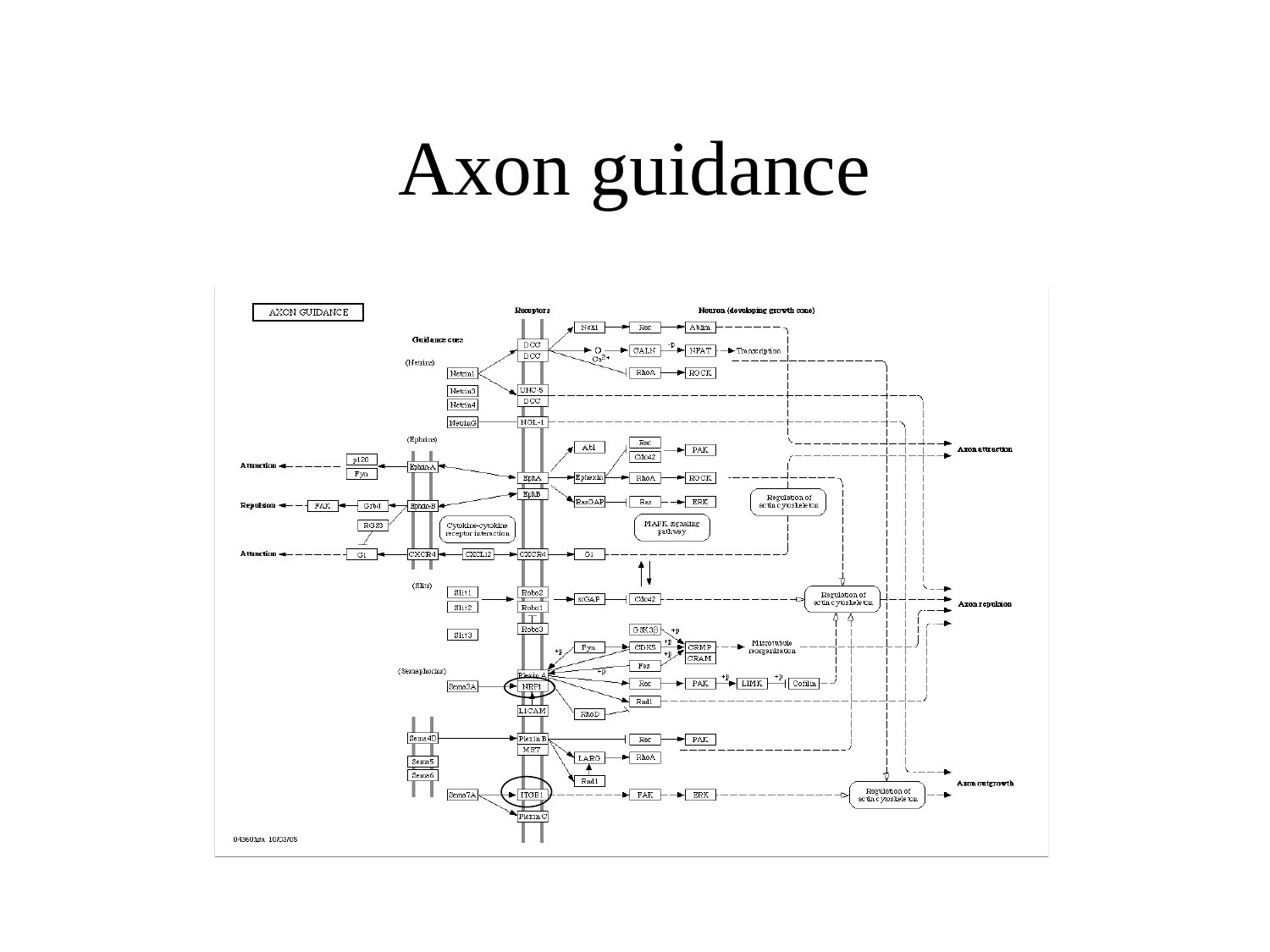

# Axon guidance

## Slide 8
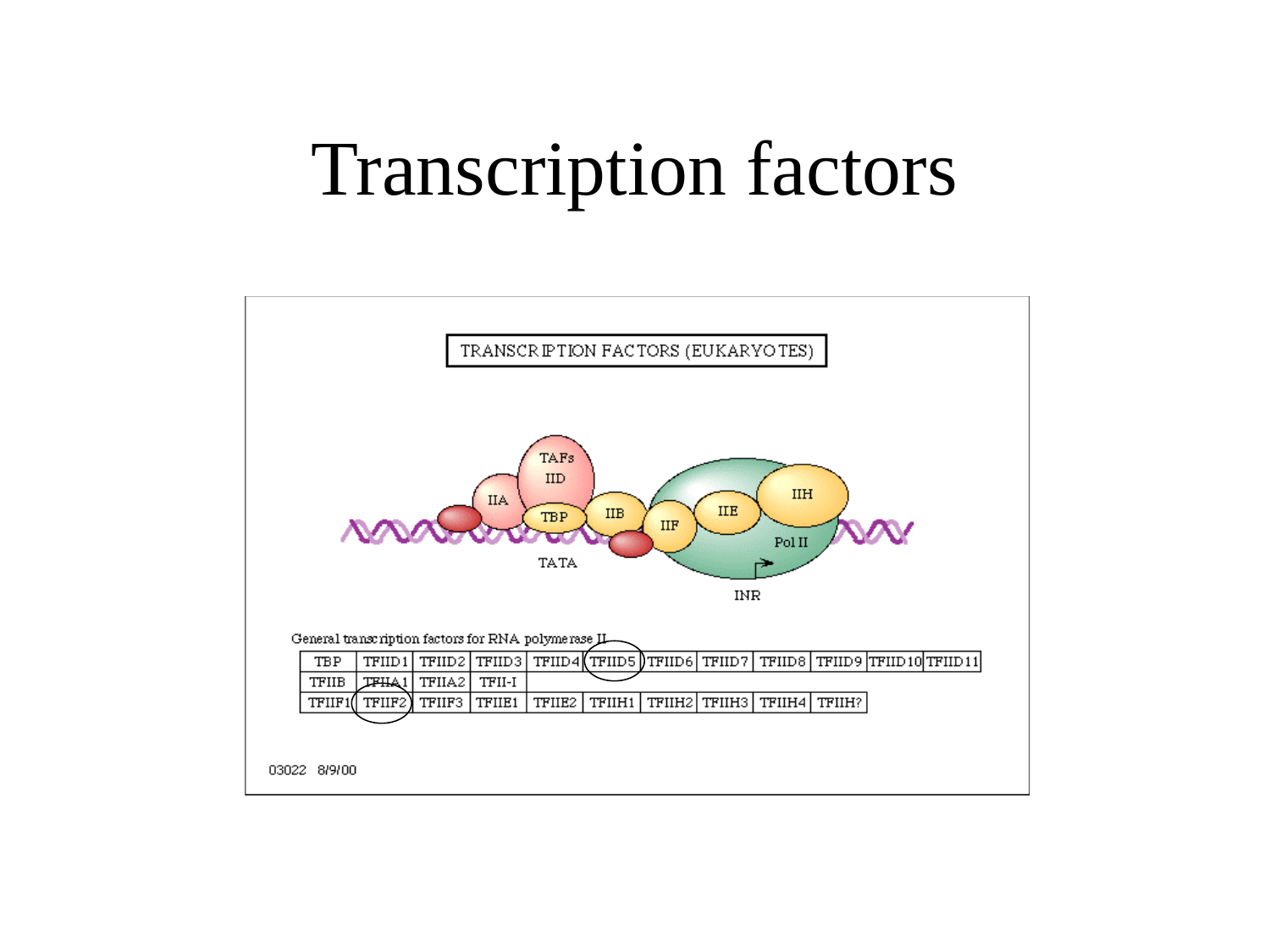

# Transcription factors

## Slide 9
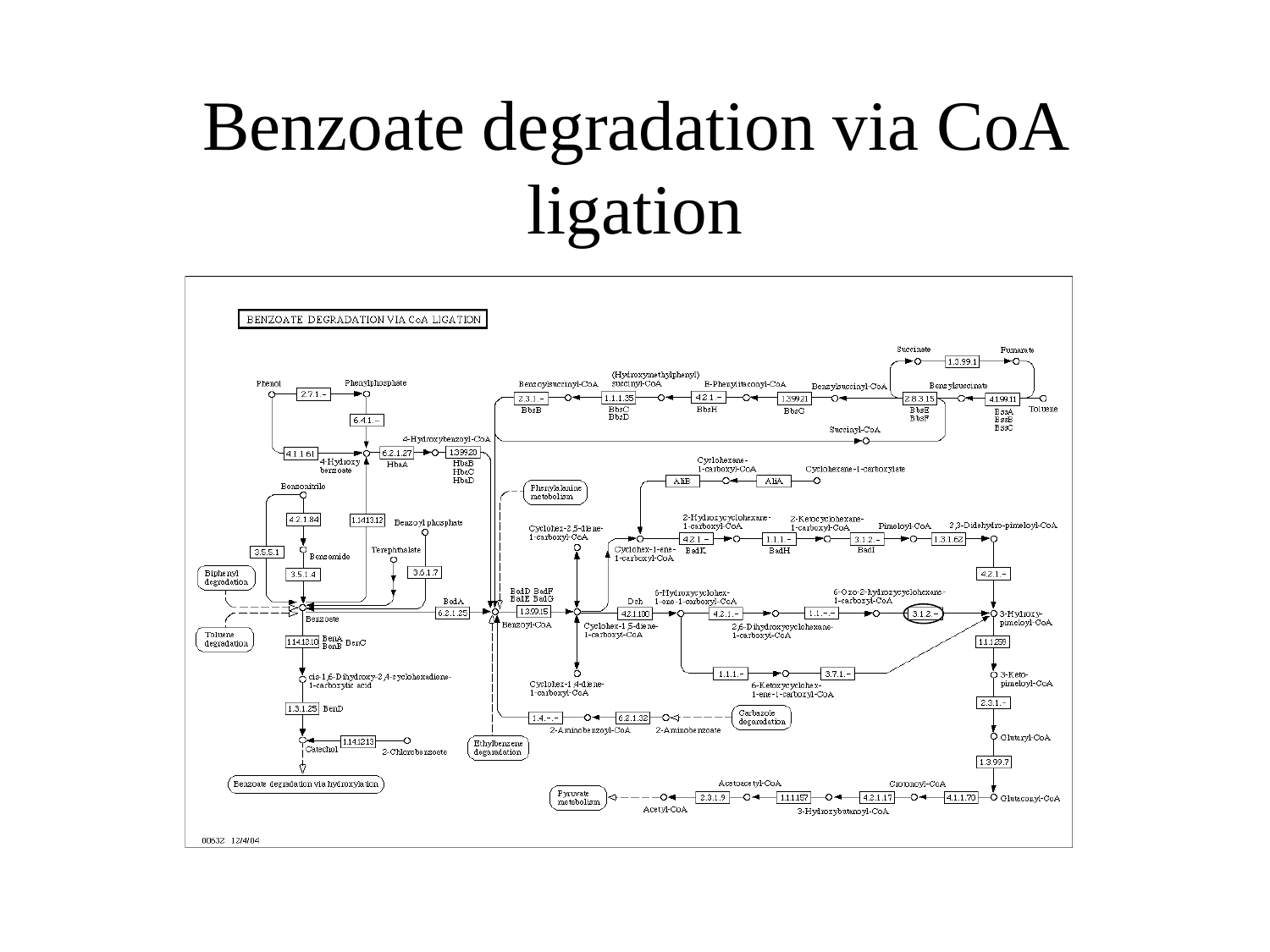

# Benzoate degradation via CoA ligation

## Slide 10
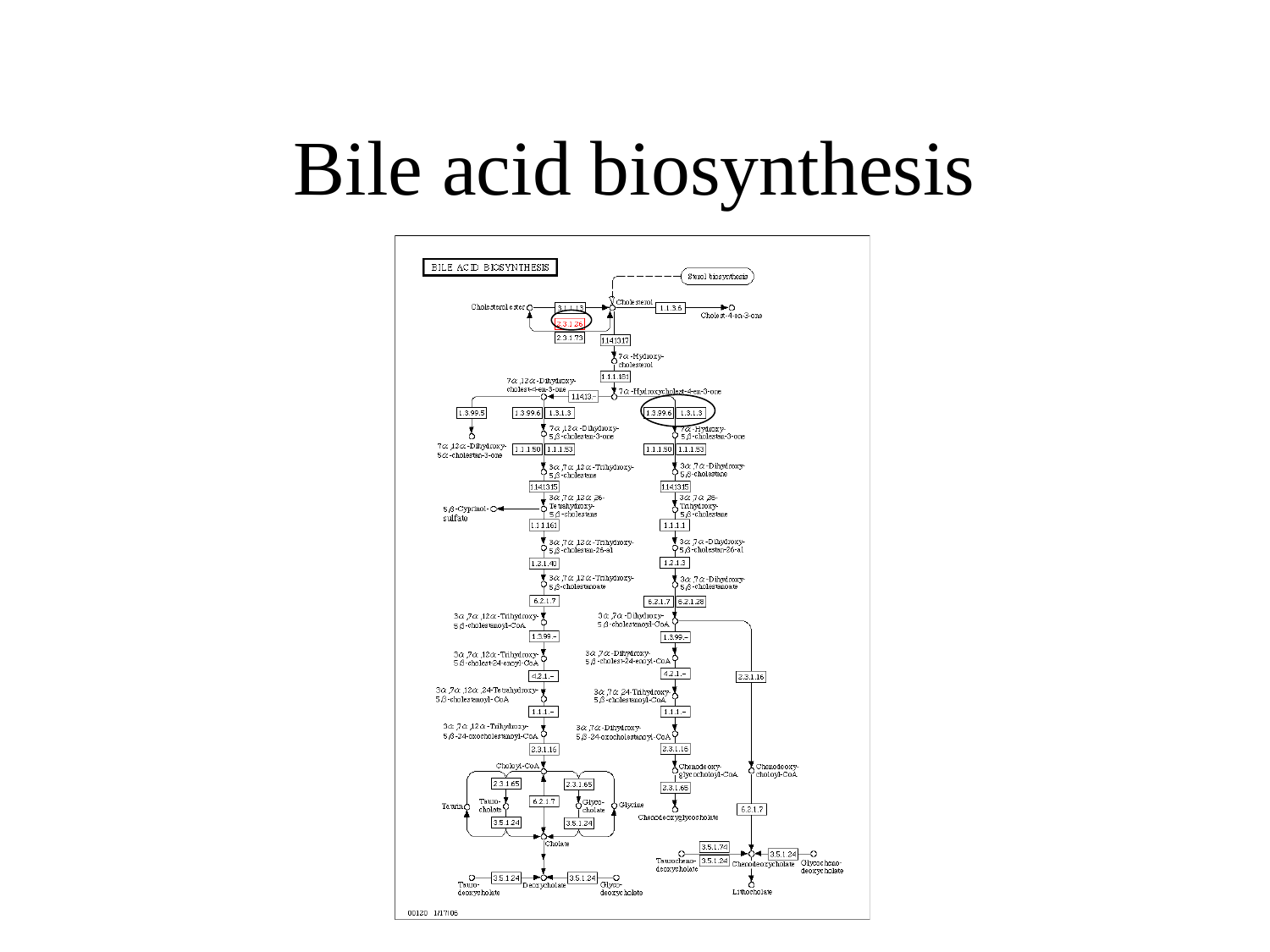

# Bile acid biosynthesis

## Slide 11
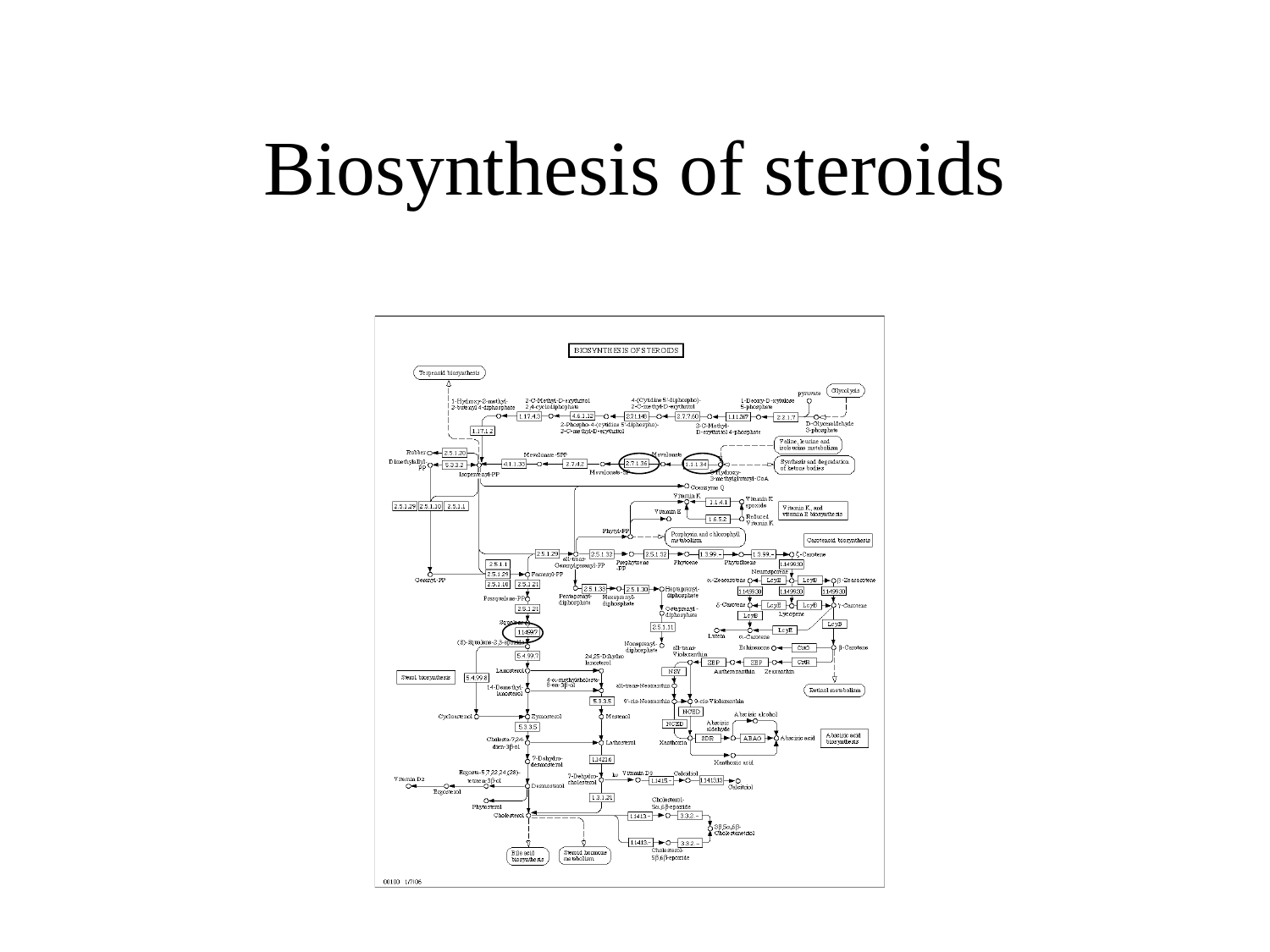

# Biosynthesis of steroids

## Slide 12
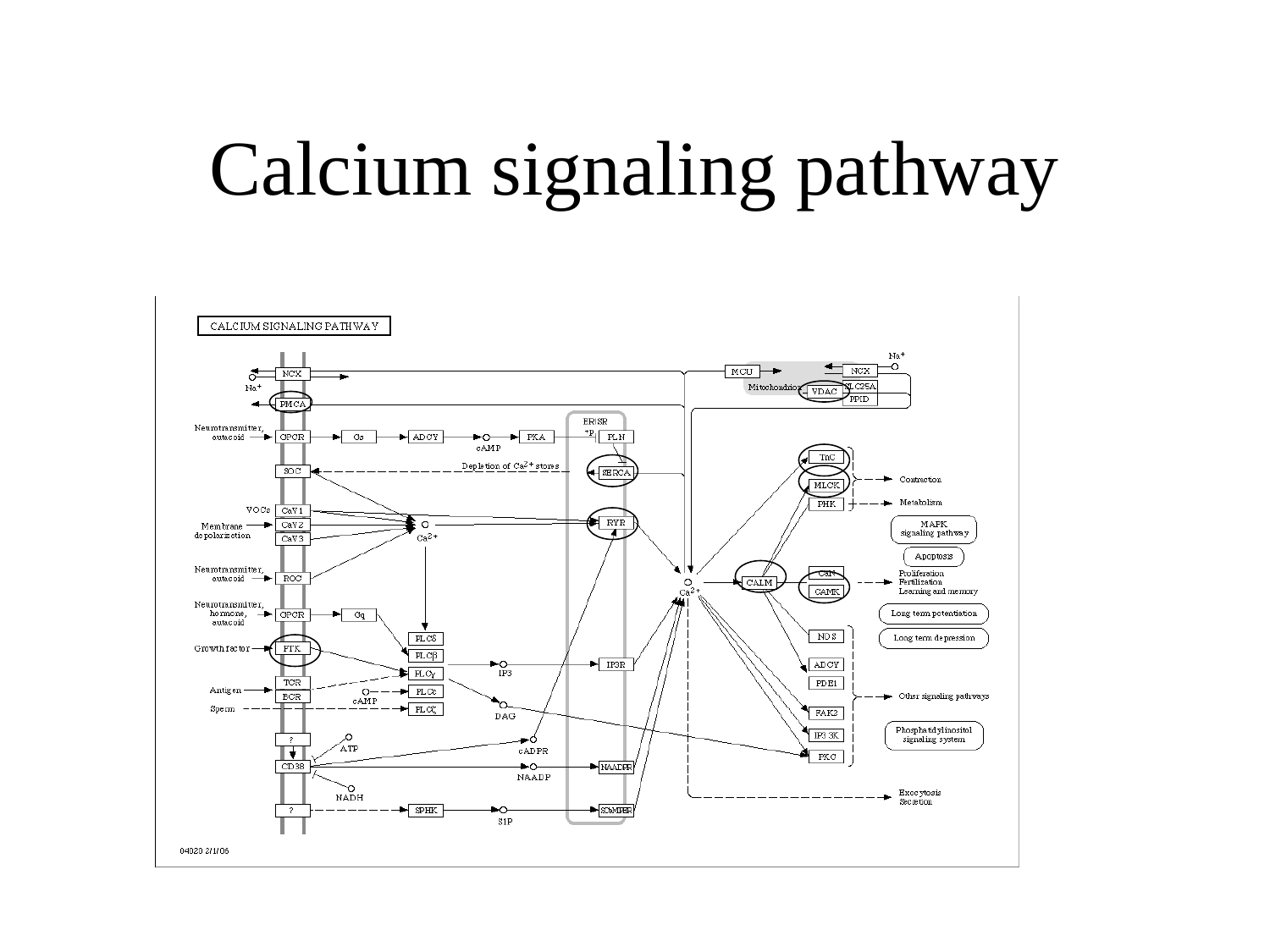

# Calcium signaling pathway

## Slide 13
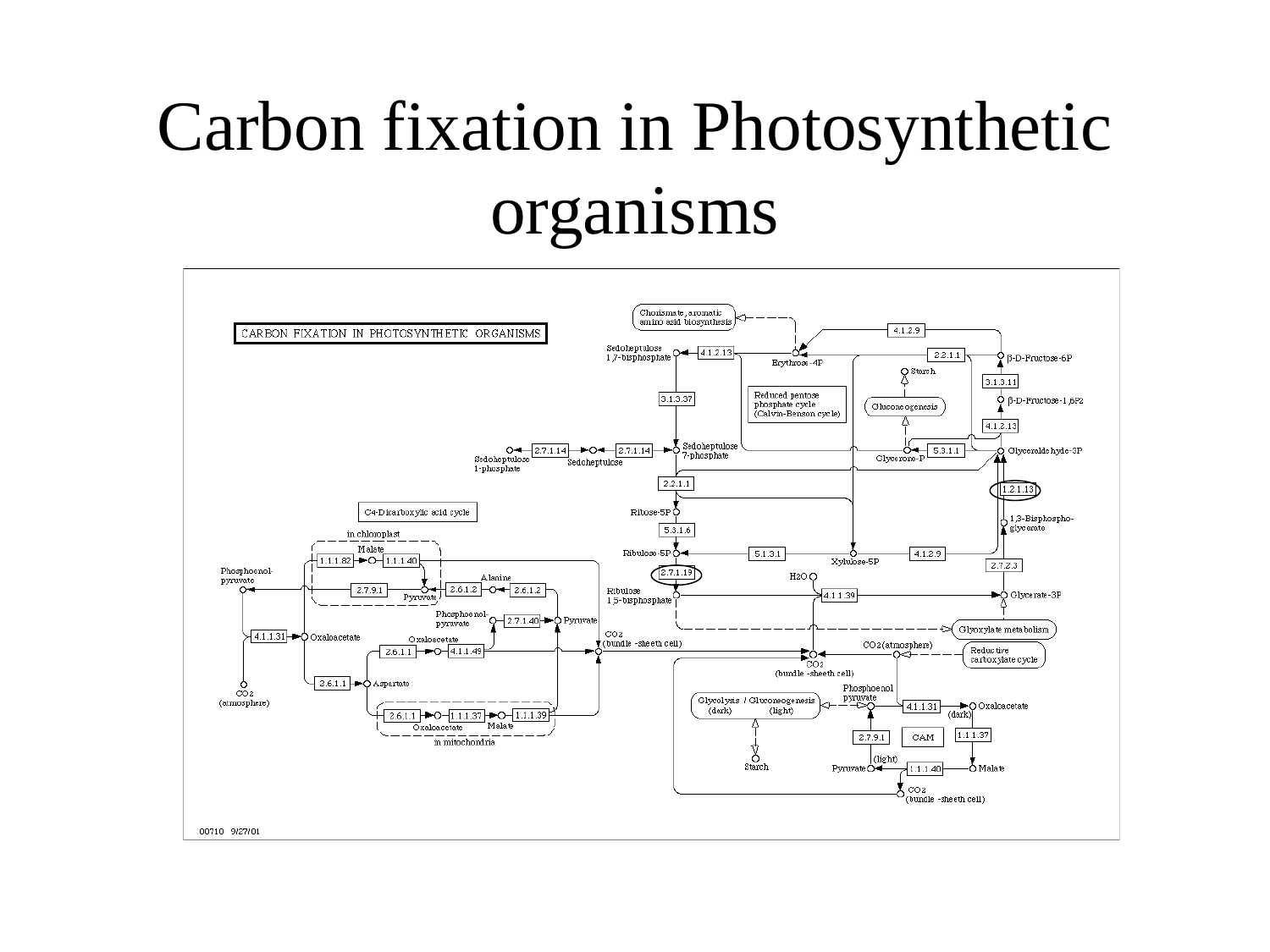

# Carbon fixation in Photosynthetic organisms

## Slide 14
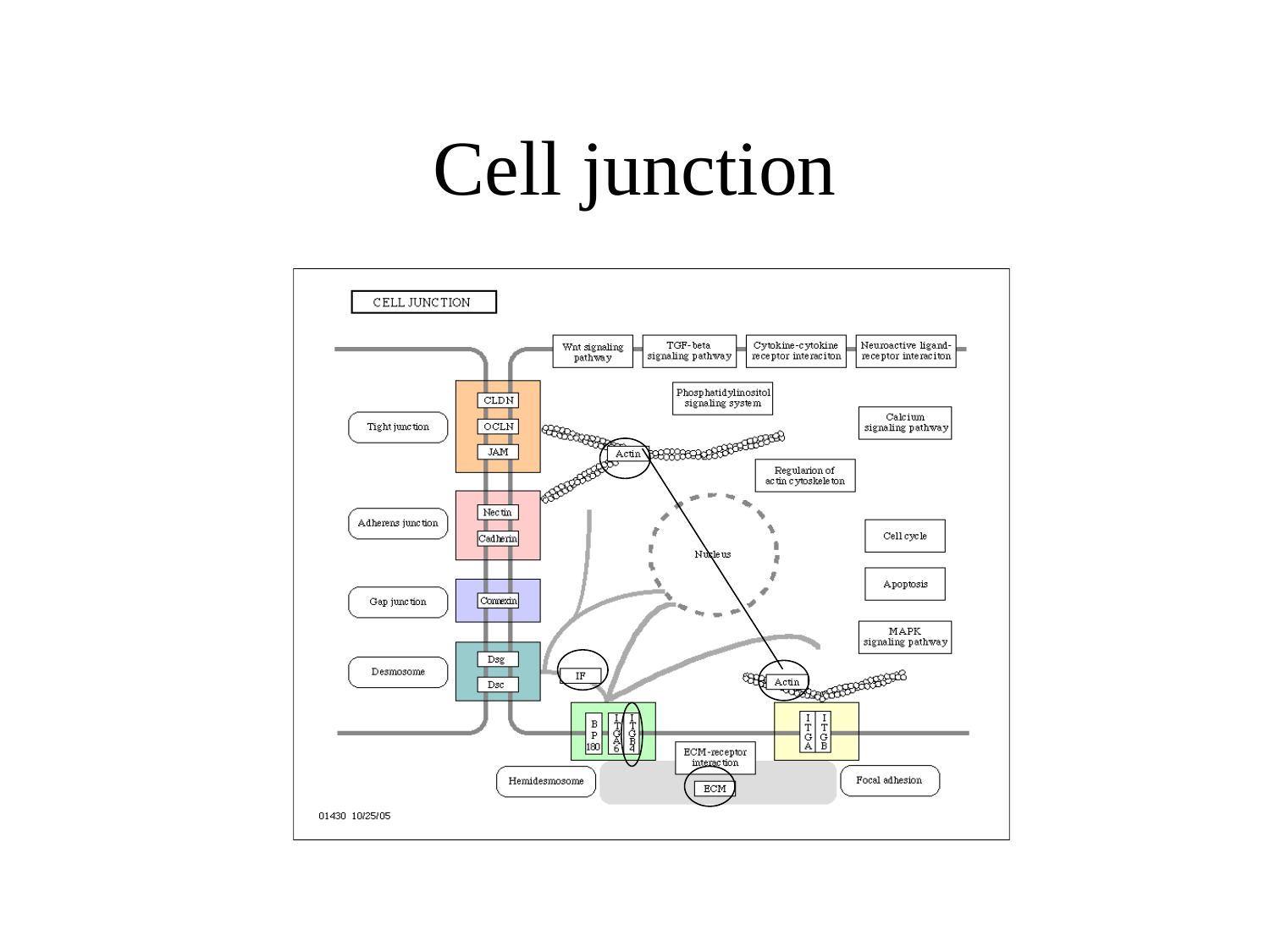

# Cell junction

## Slide 15
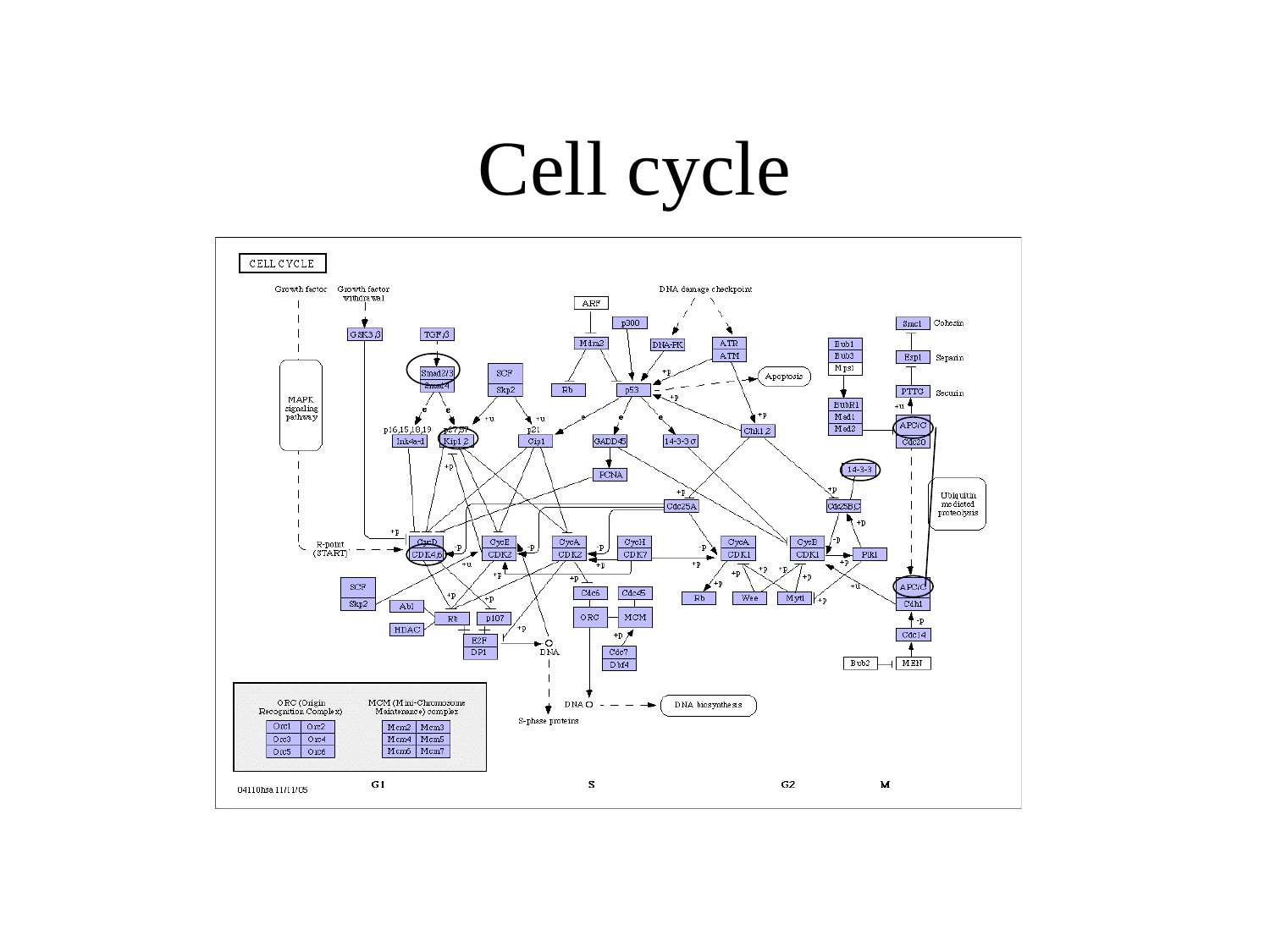

# Cell cycle

## Slide 16
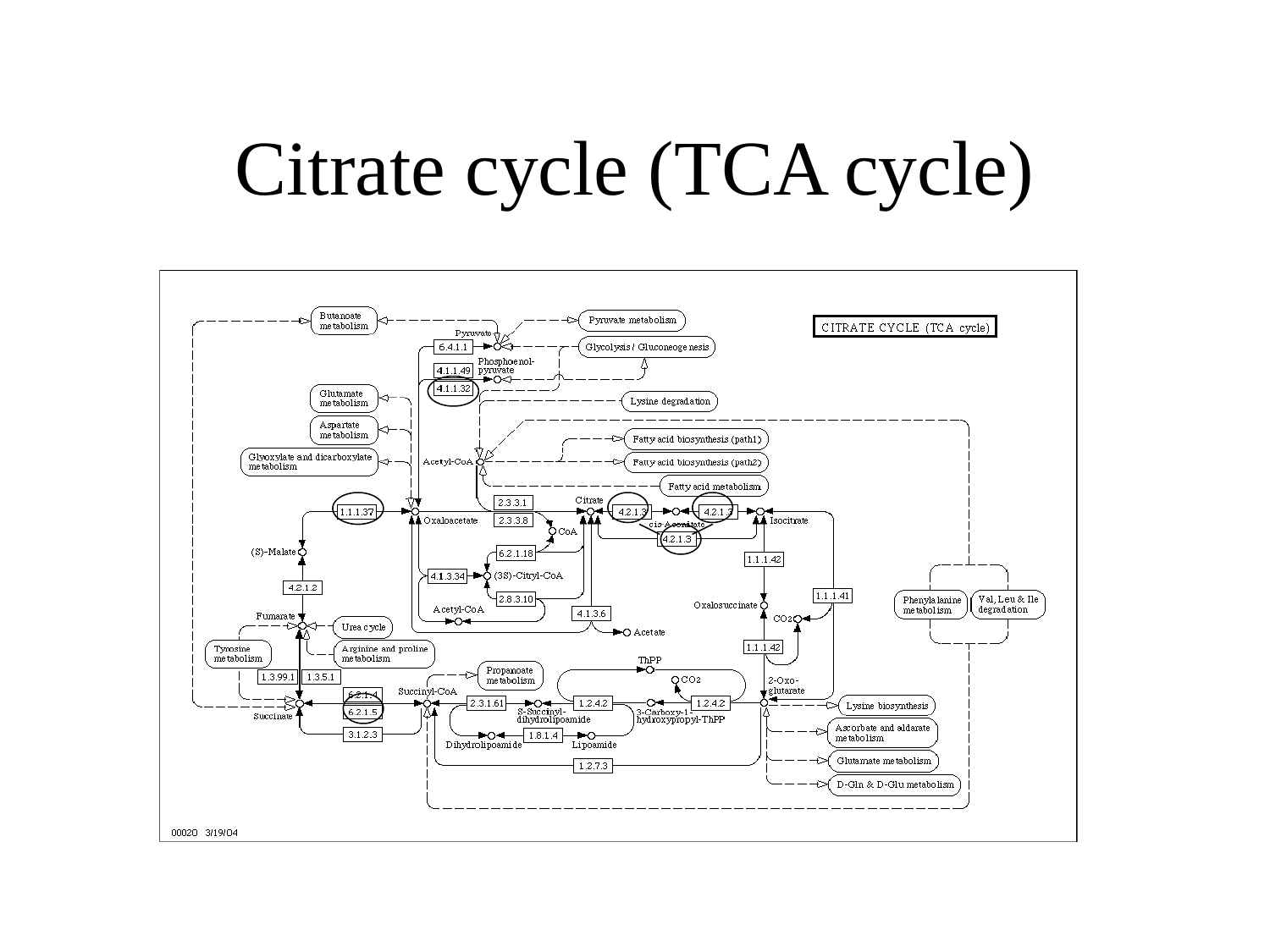

# Citrate cycle (TCA cycle)

## Slide 17
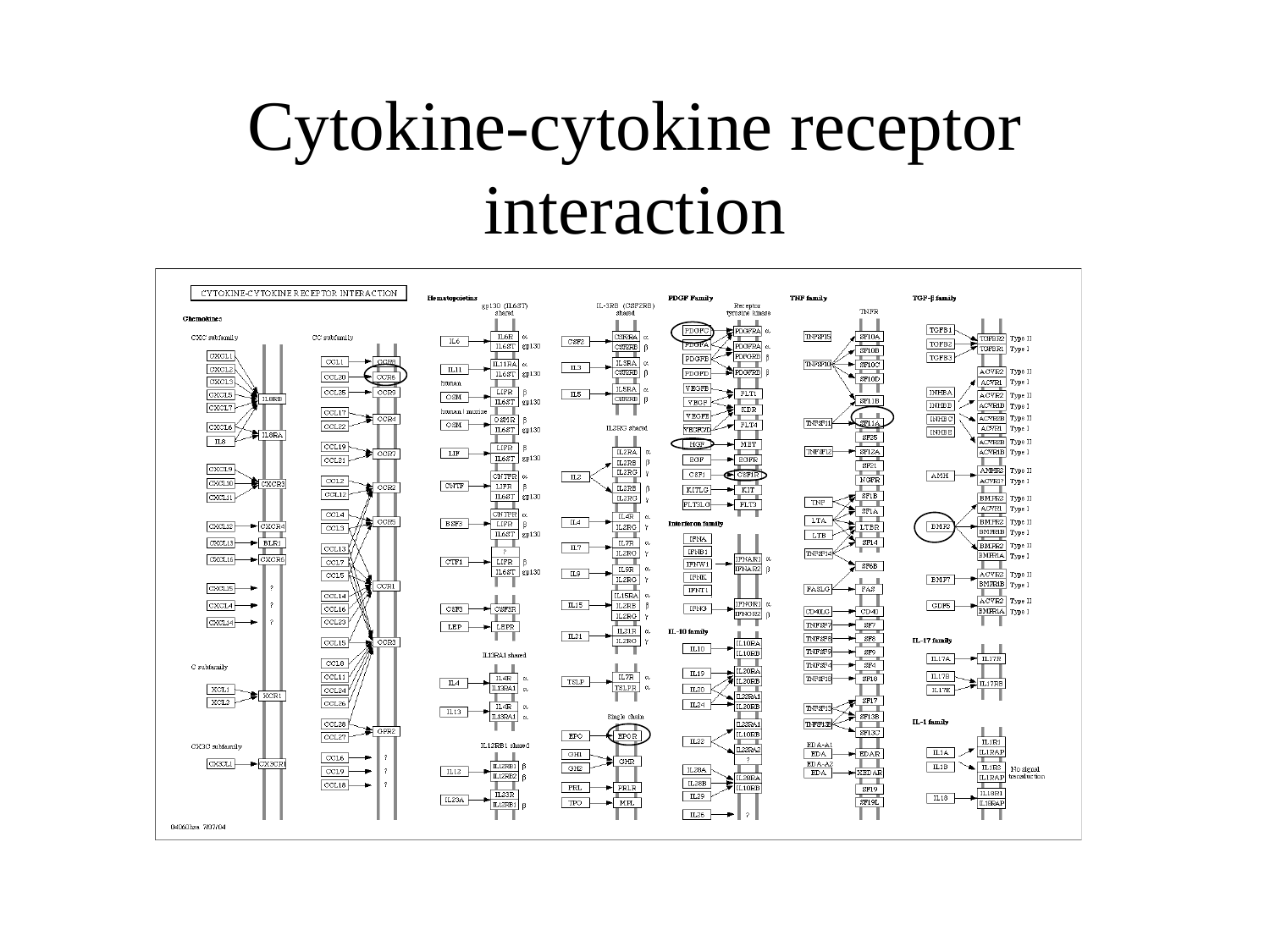

# Cytokine-cytokine receptor interaction

## Slide 18
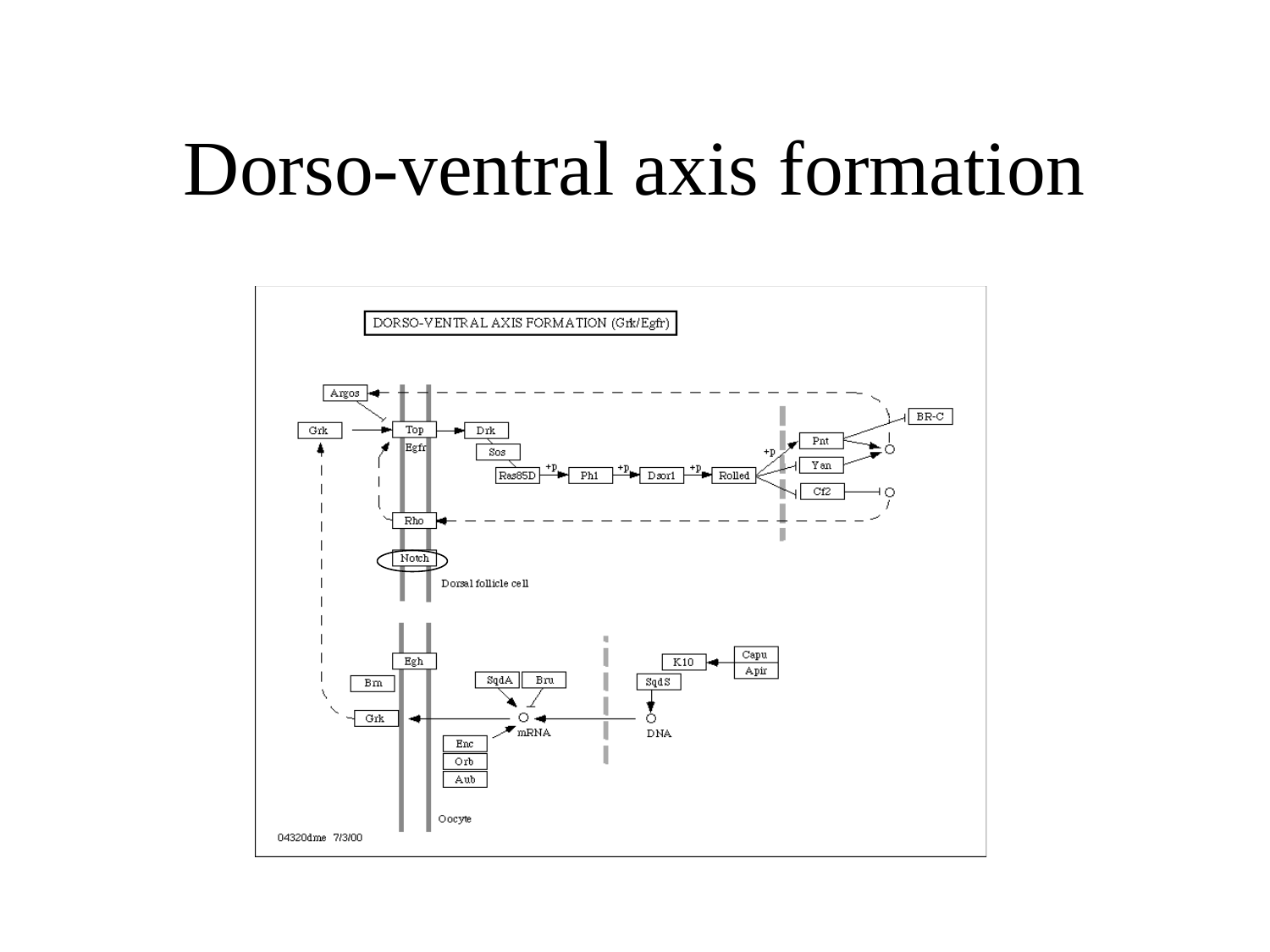

# Dorso-ventral axis formation

## Slide 19
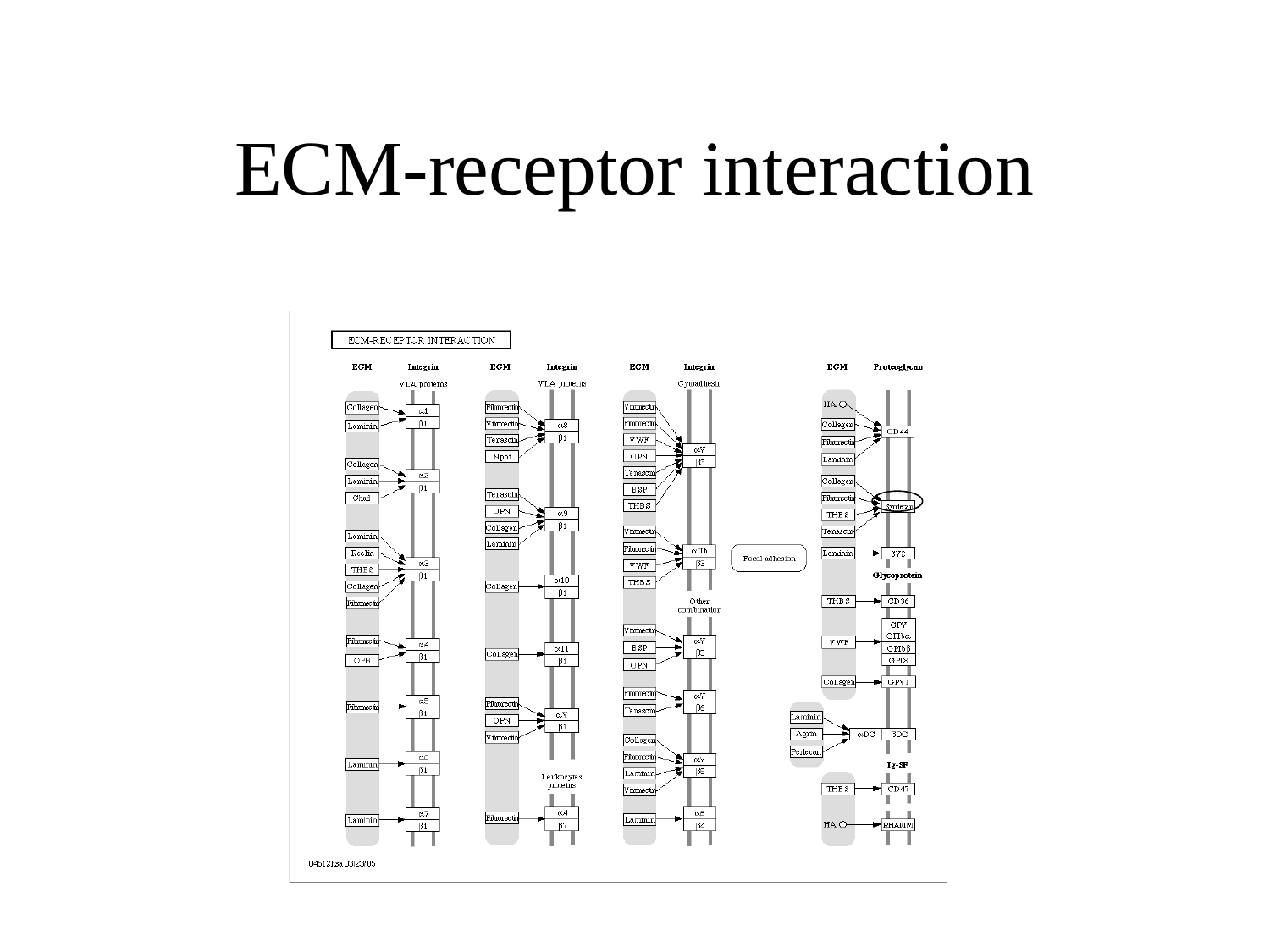

# ECM-receptor interaction

## Slide 20
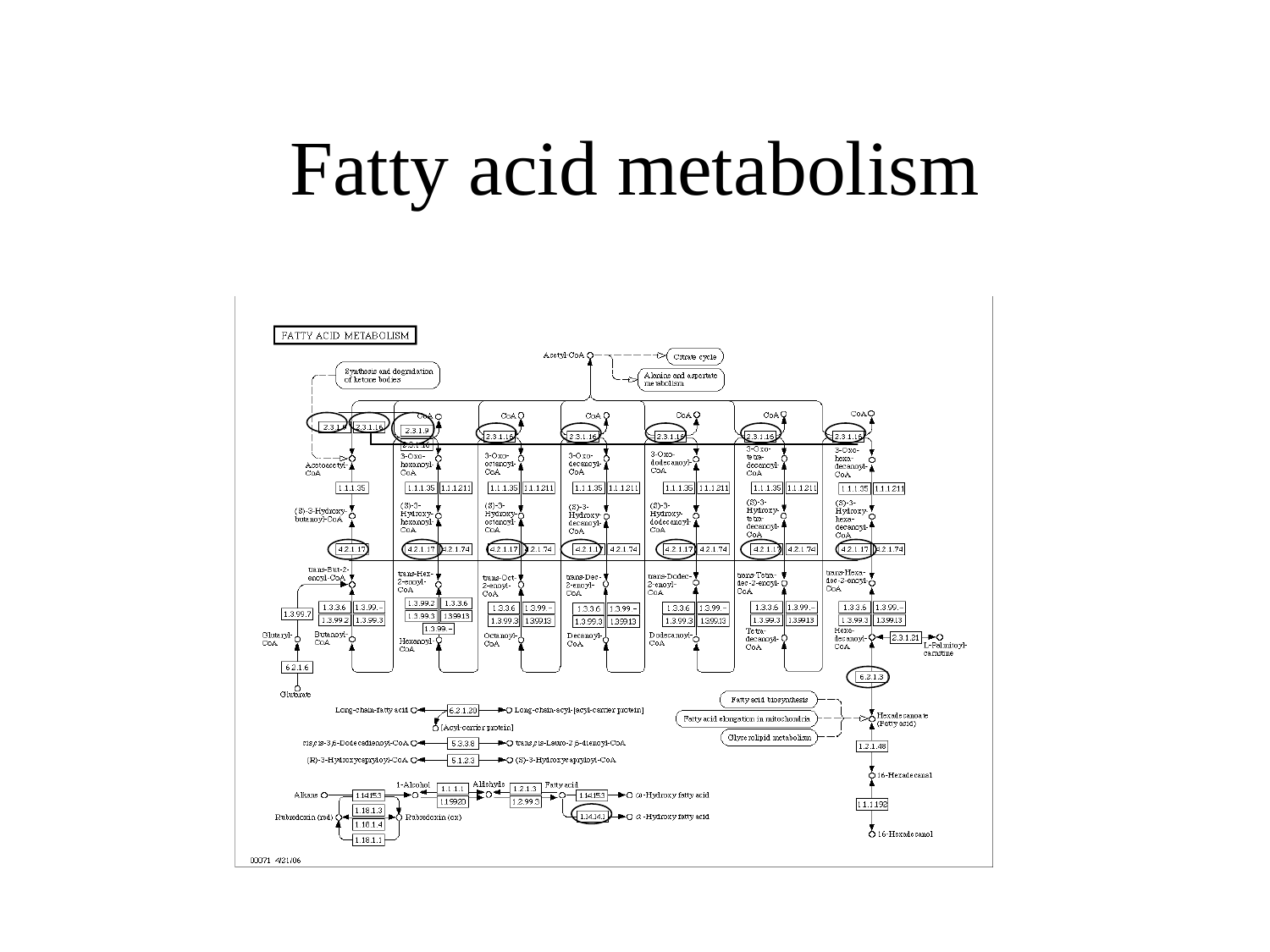

# Fatty acid metabolism

## Slide 21
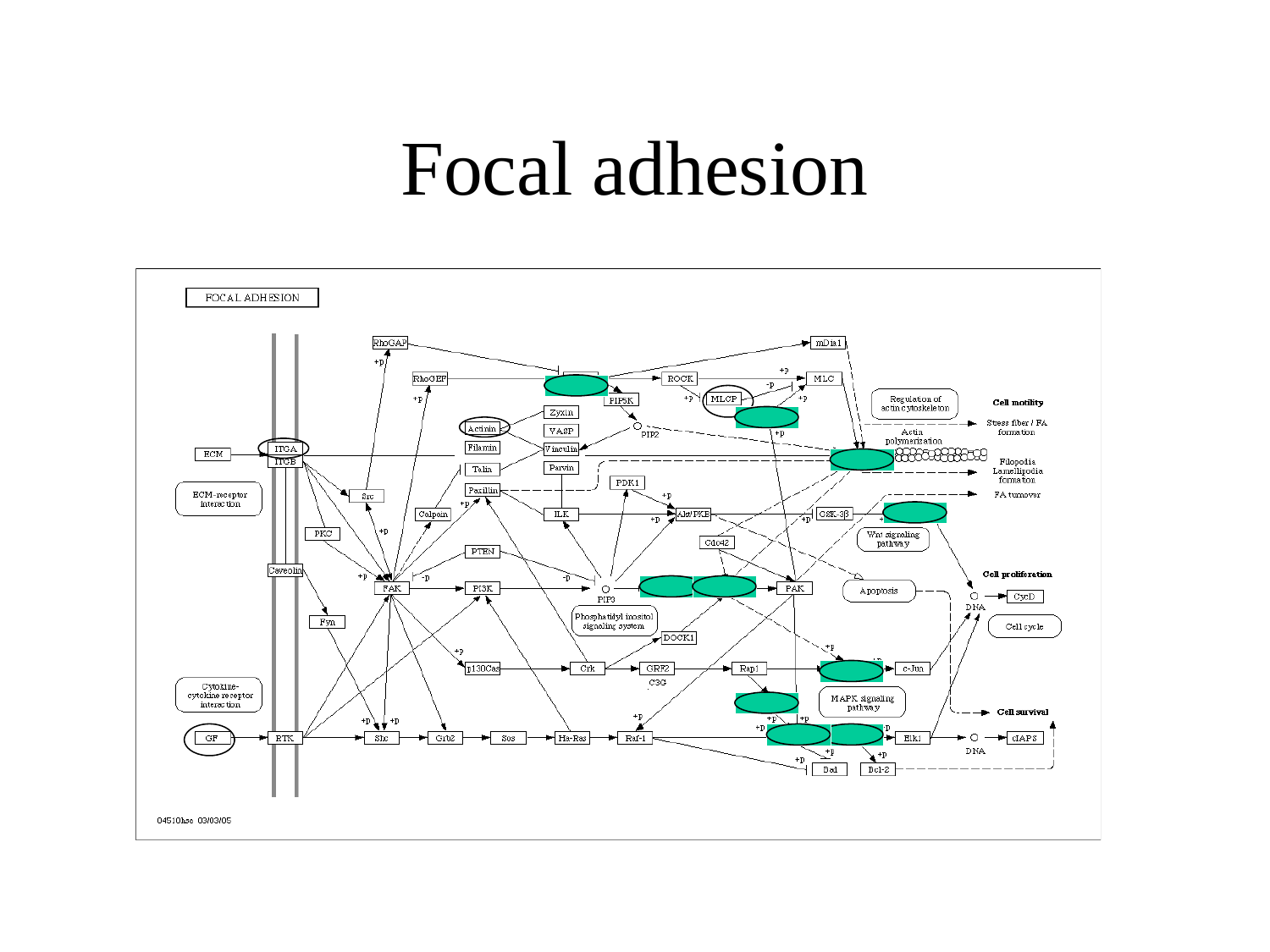

# Focal adhesion

## Slide 22
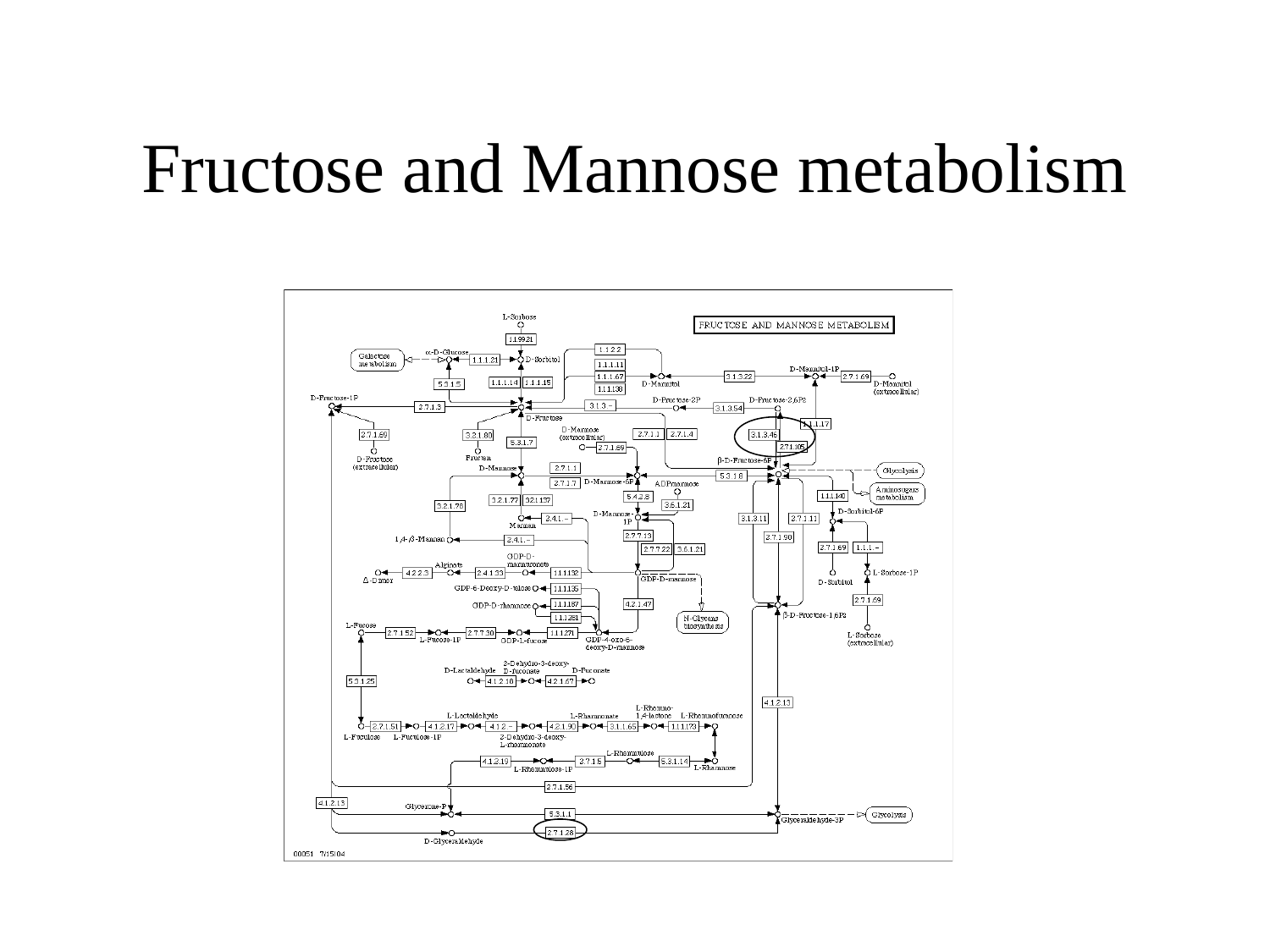

# Fructose and Mannose metabolism

## Slide 23
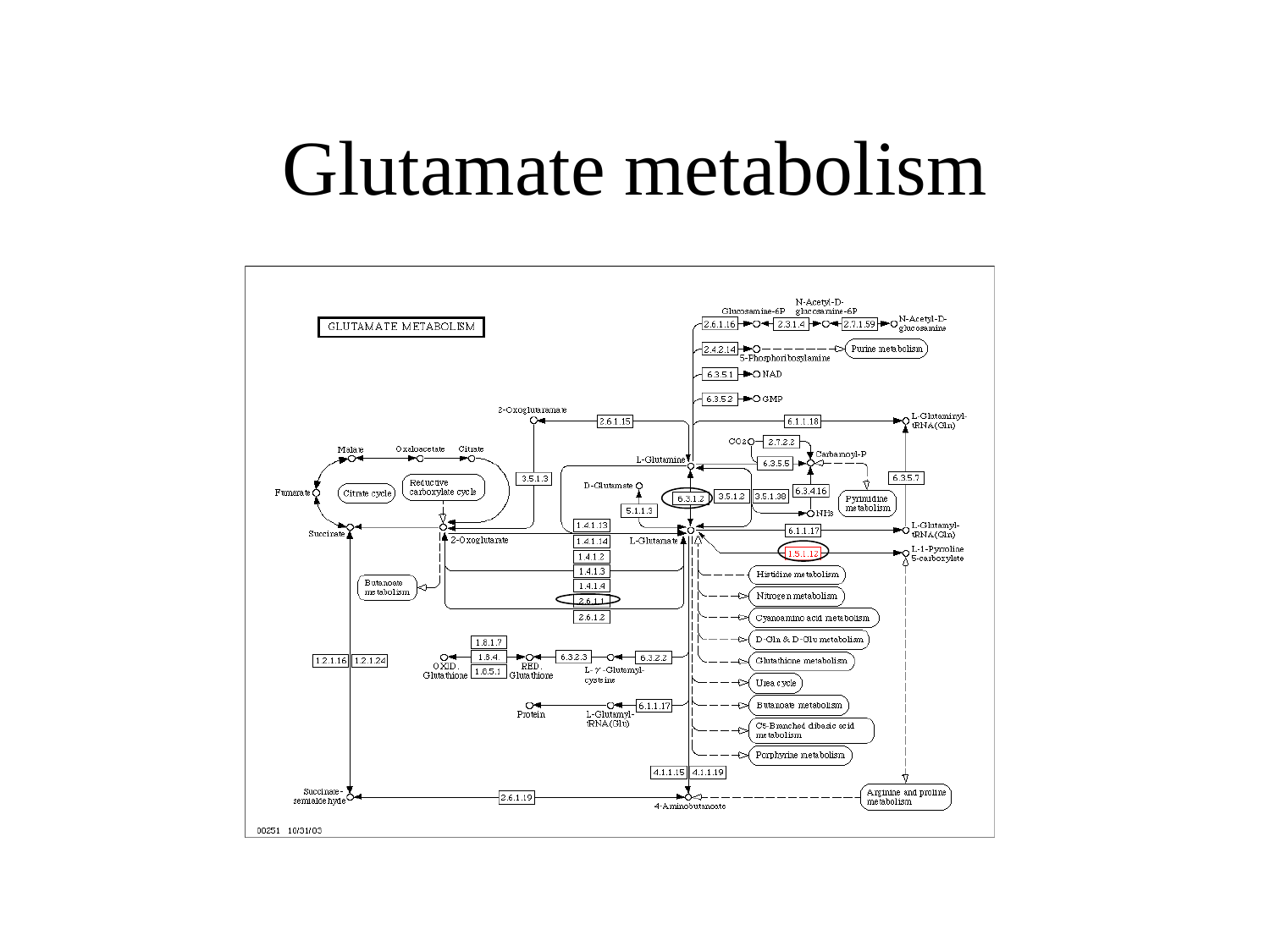

# Glutamate metabolism

## Slide 24
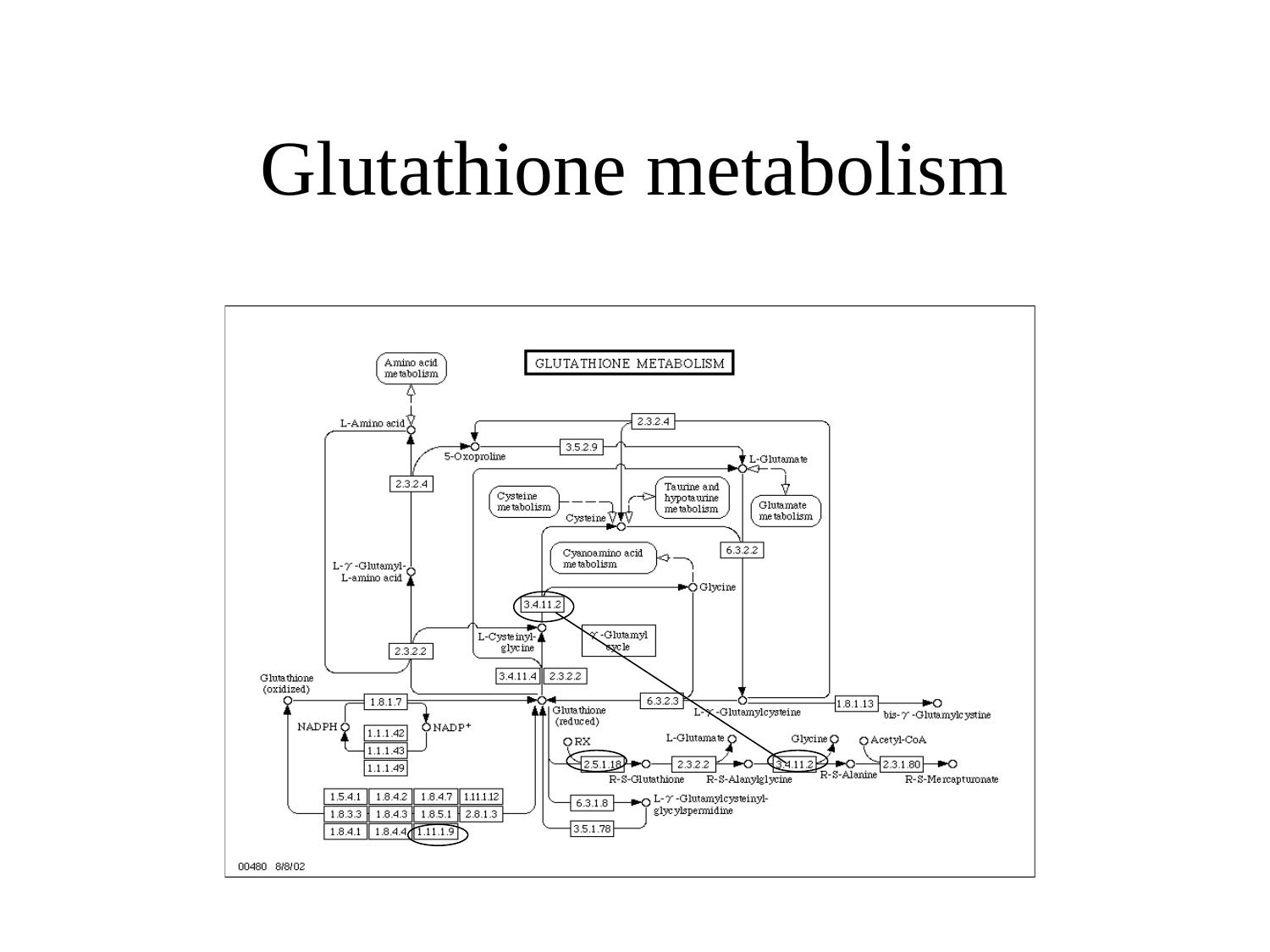

# Glutathione metabolism

## Slide 25
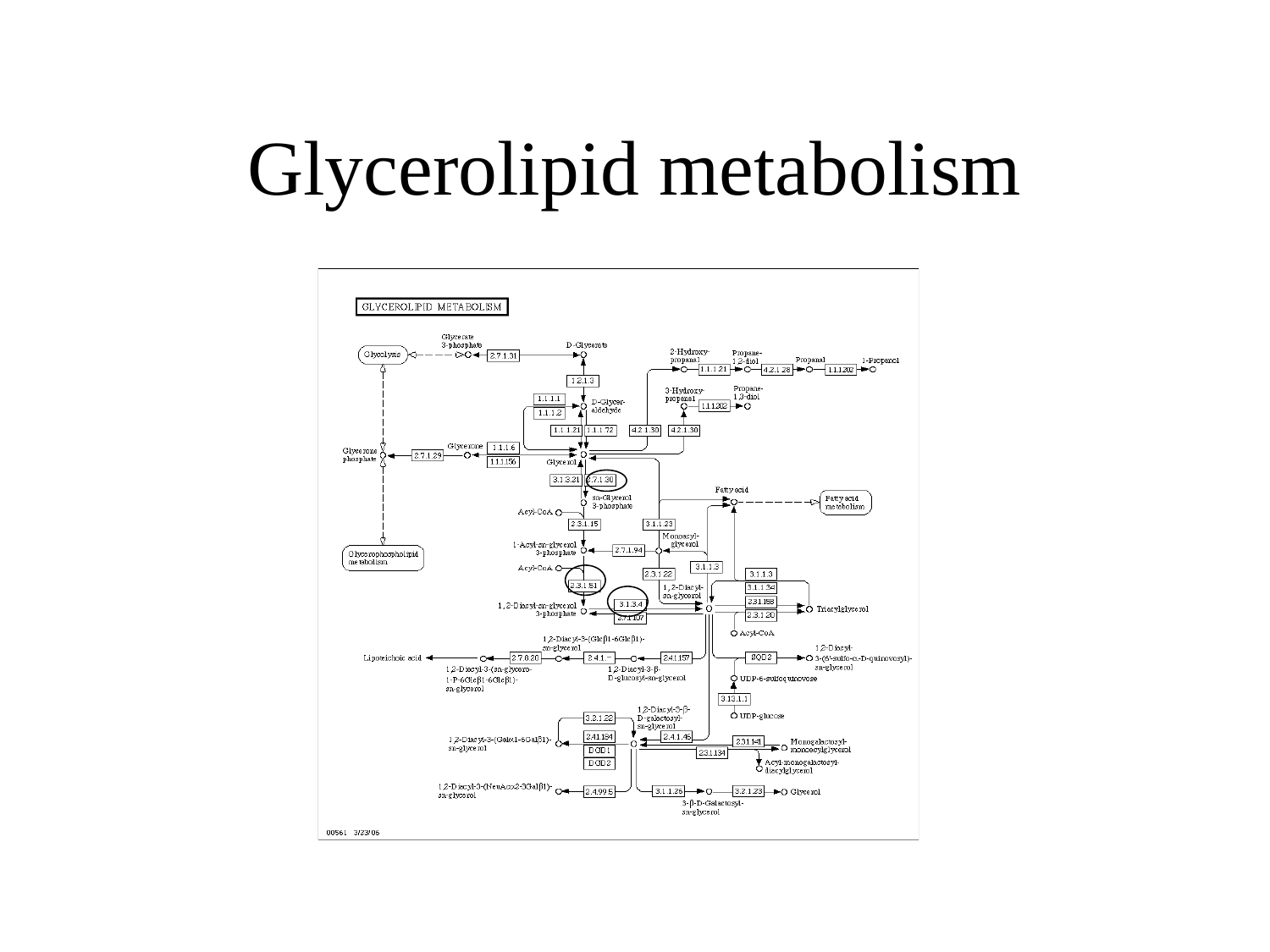

# Glycerolipid metabolism

## Slide 26
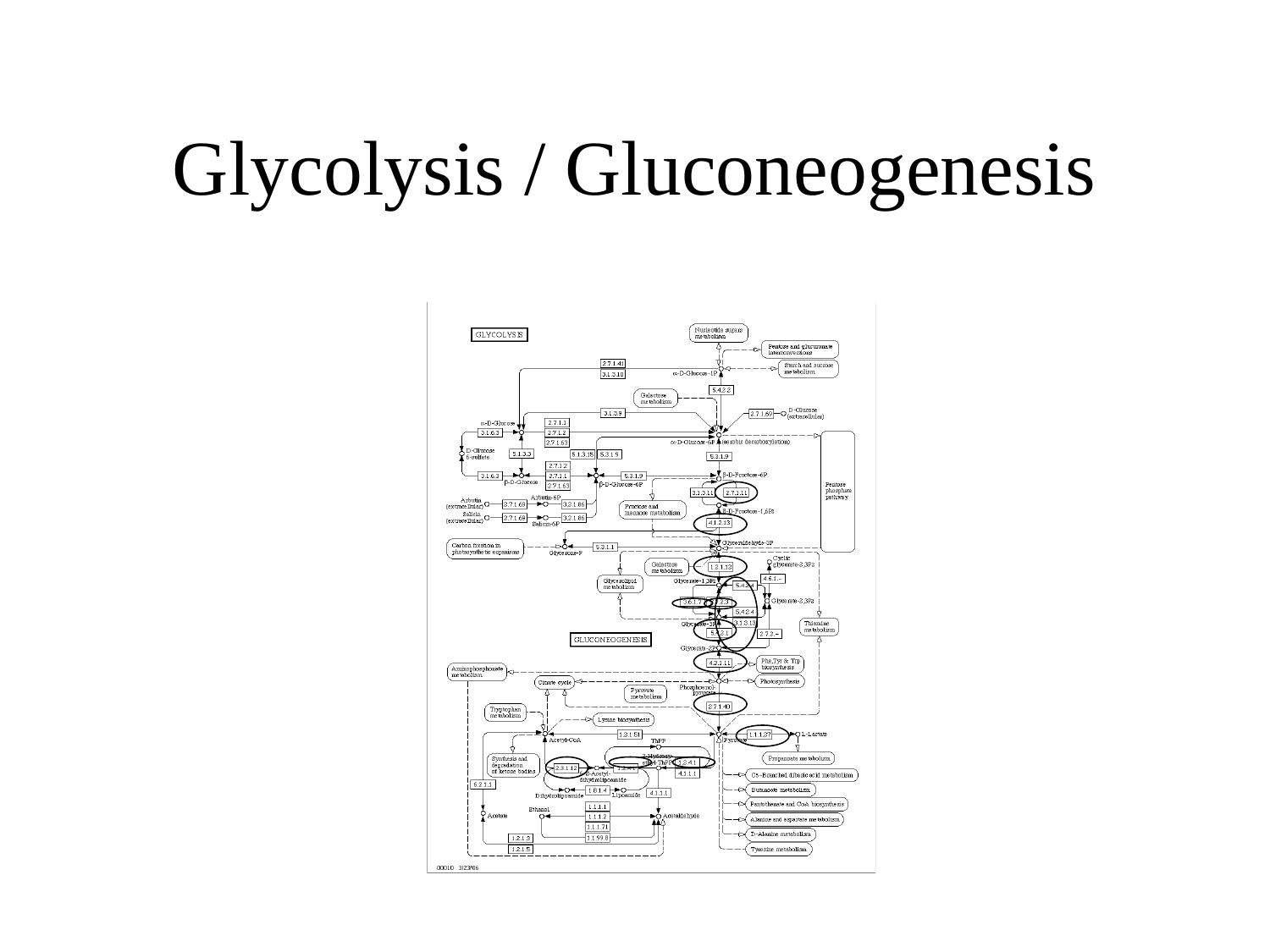

# Glycolysis / Gluconeogenesis

## Slide 27
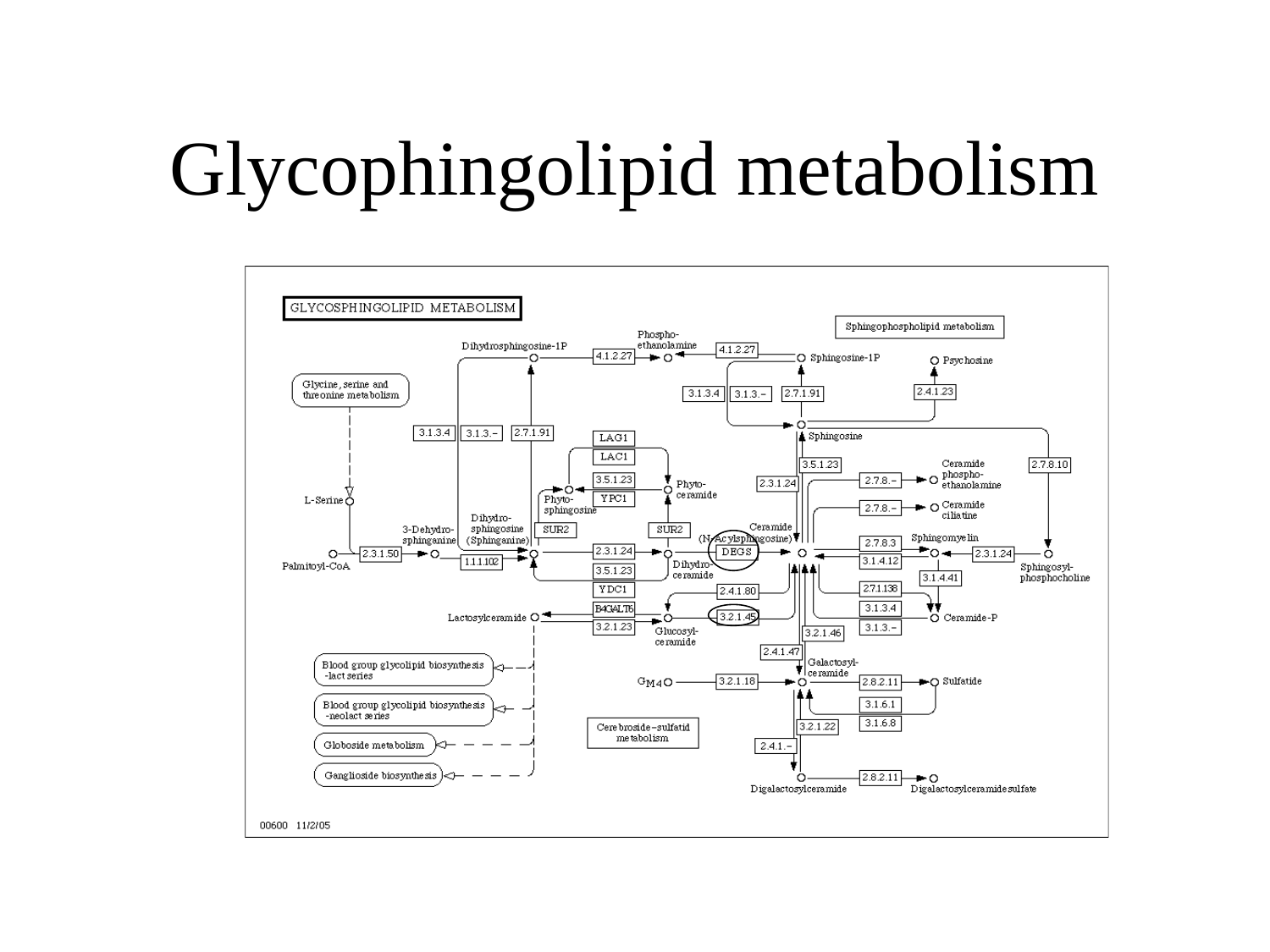

# Glycophingolipid metabolism

## Slide 28
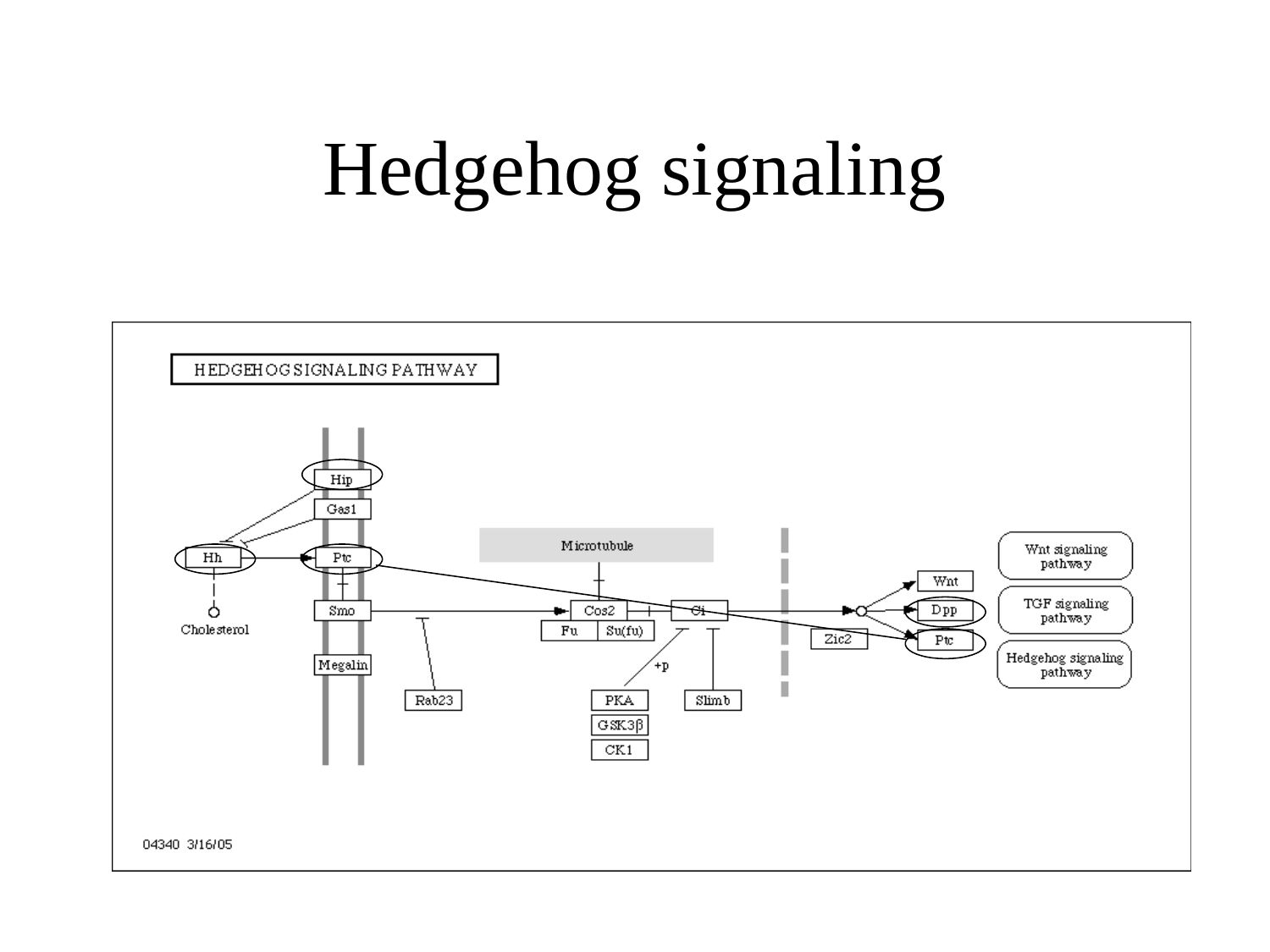

# Hedgehog signaling

## Slide 29
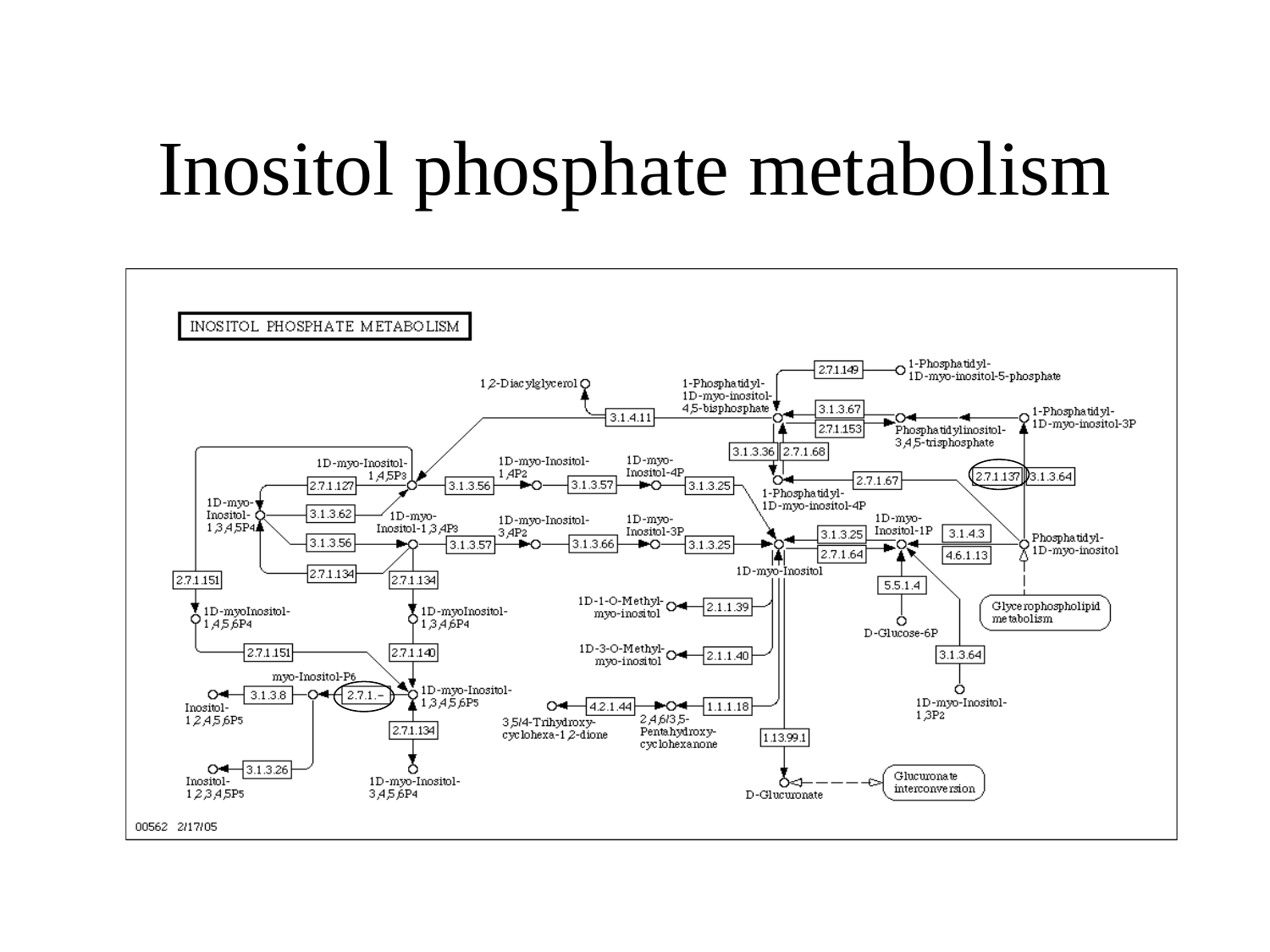

# Inositol phosphate metabolism

## Slide 30
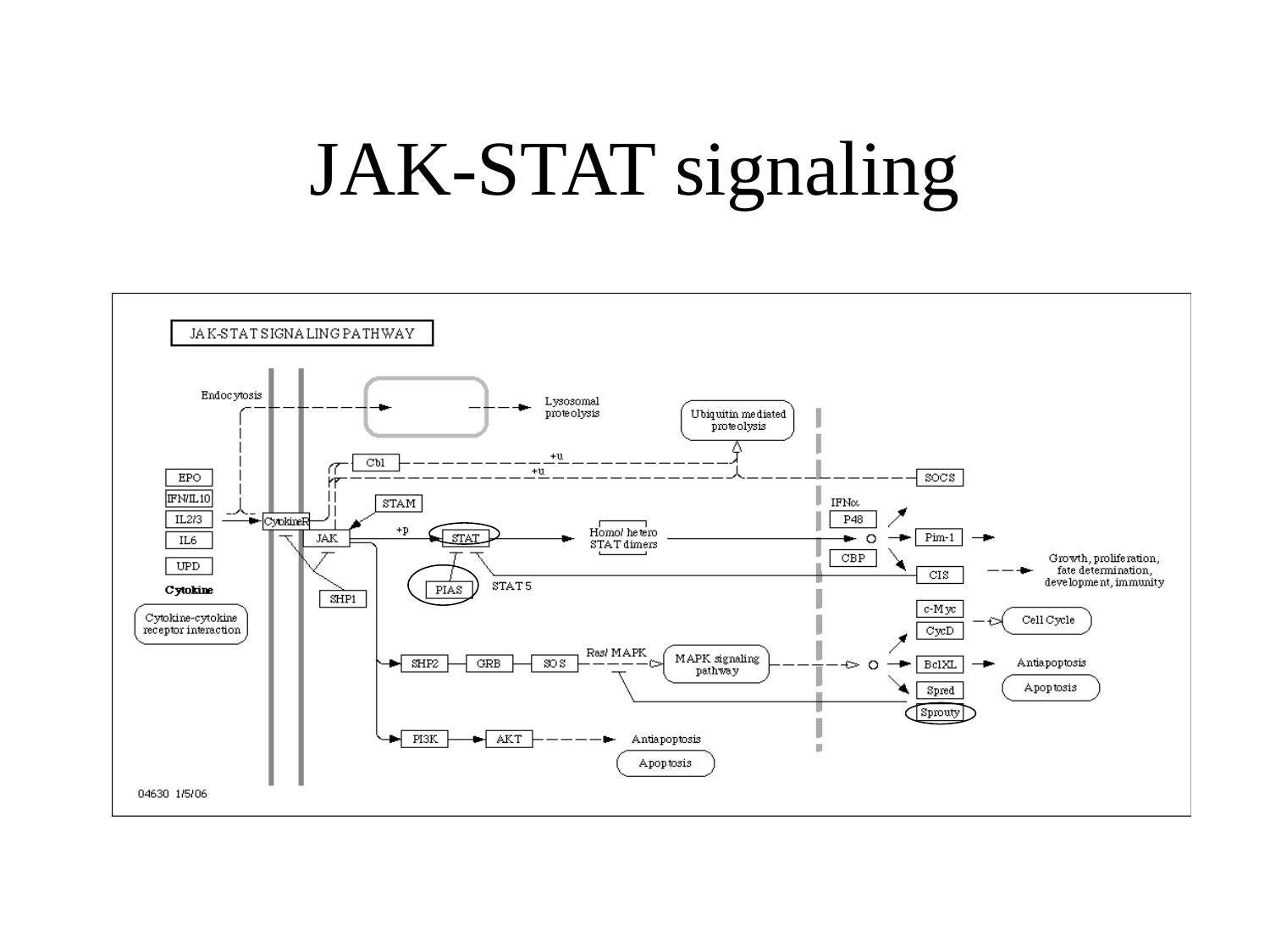

# JAK-STAT signaling

## Slide 31
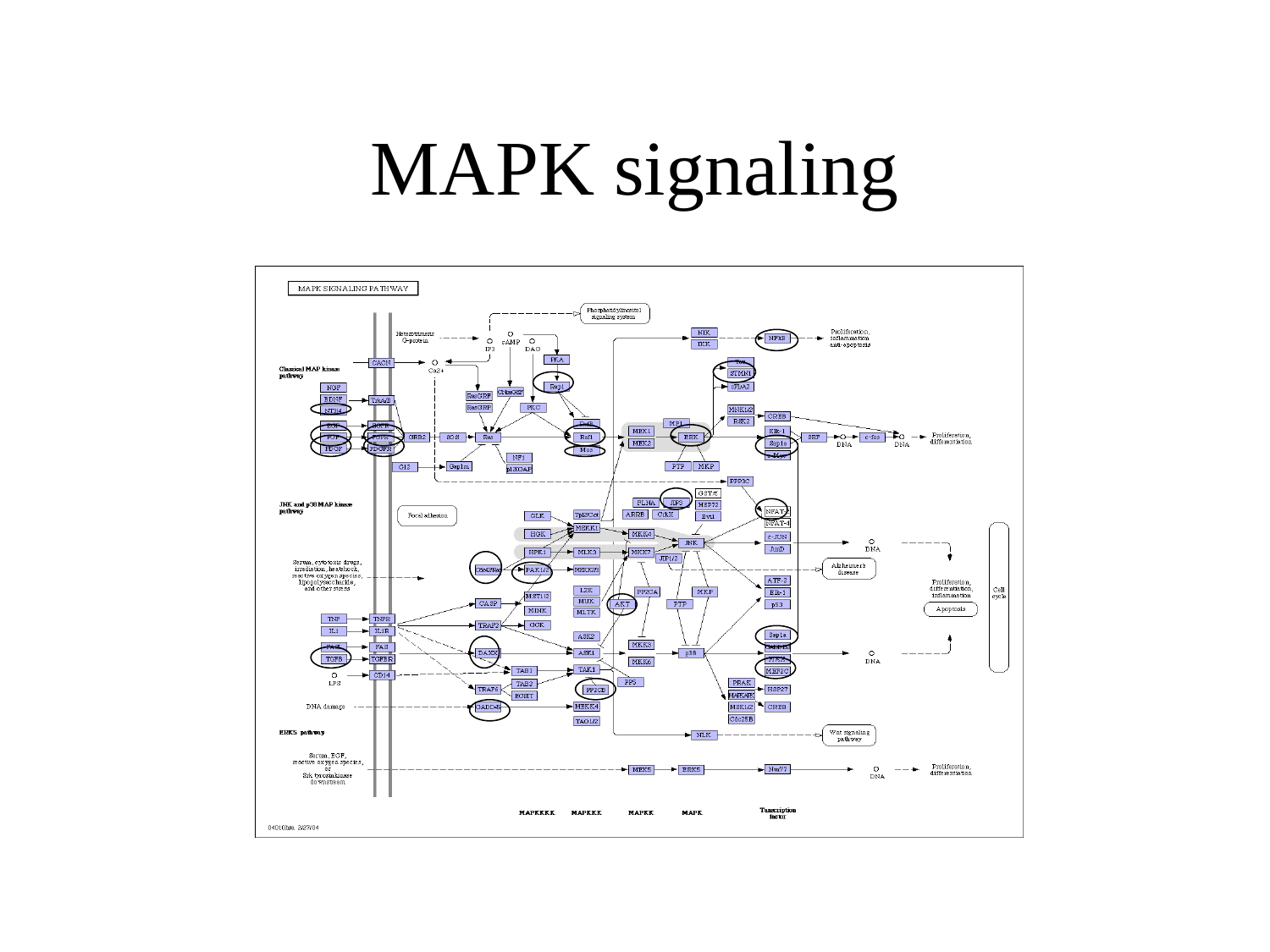

# MAPK signaling

## Slide 32
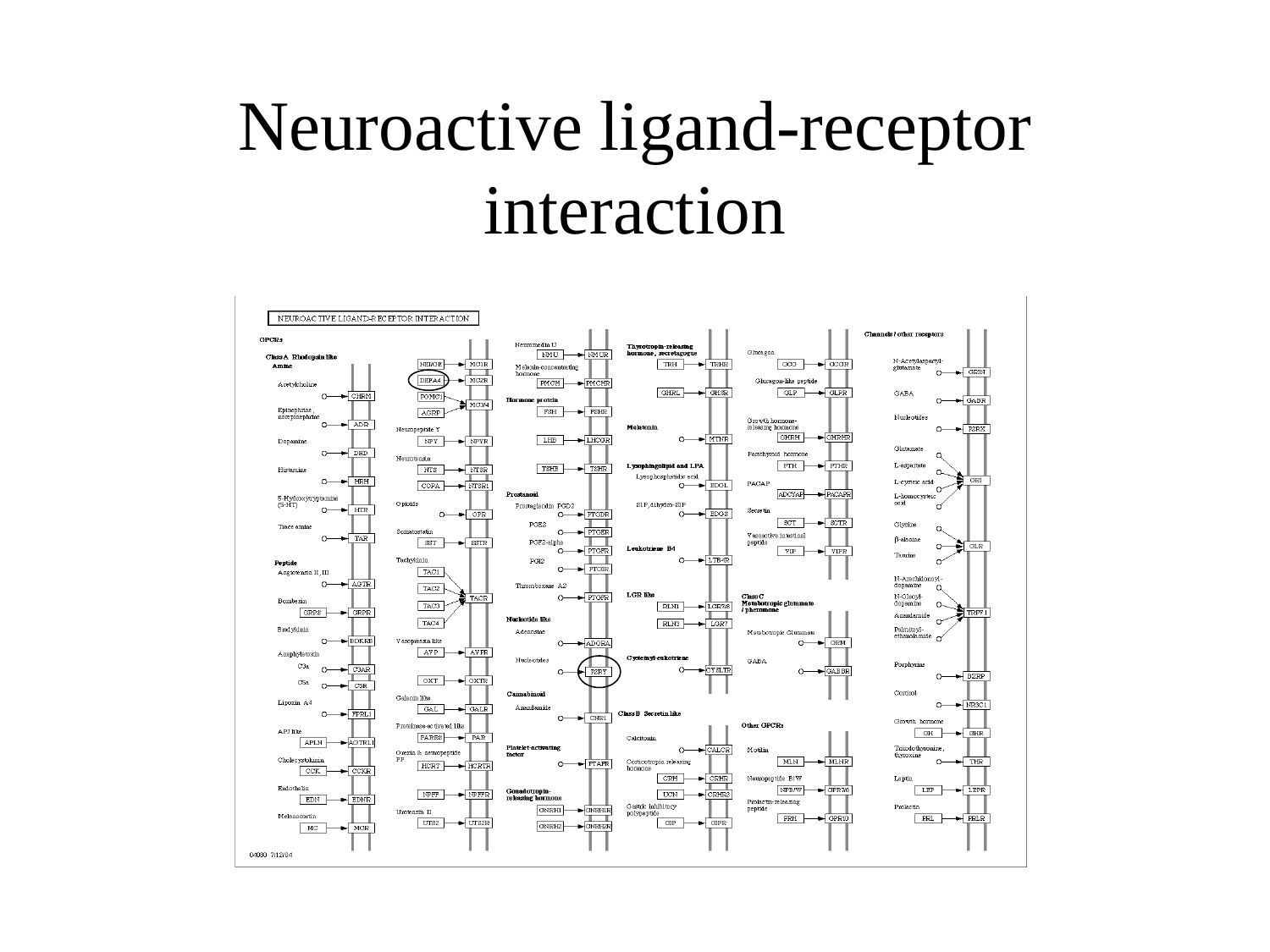

# Neuroactive ligand-receptor interaction

## Slide 33
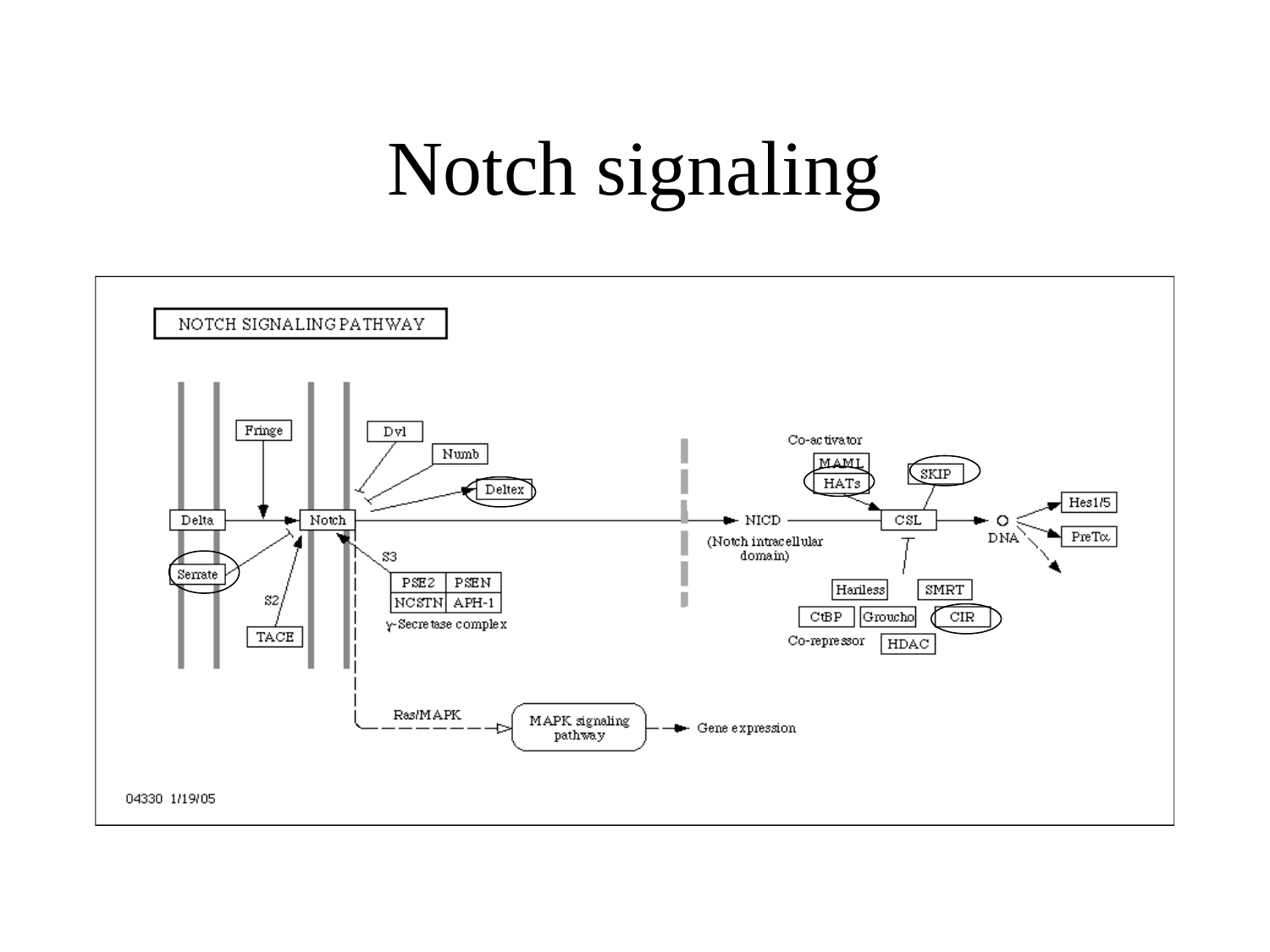

# Notch signaling

## Slide 34
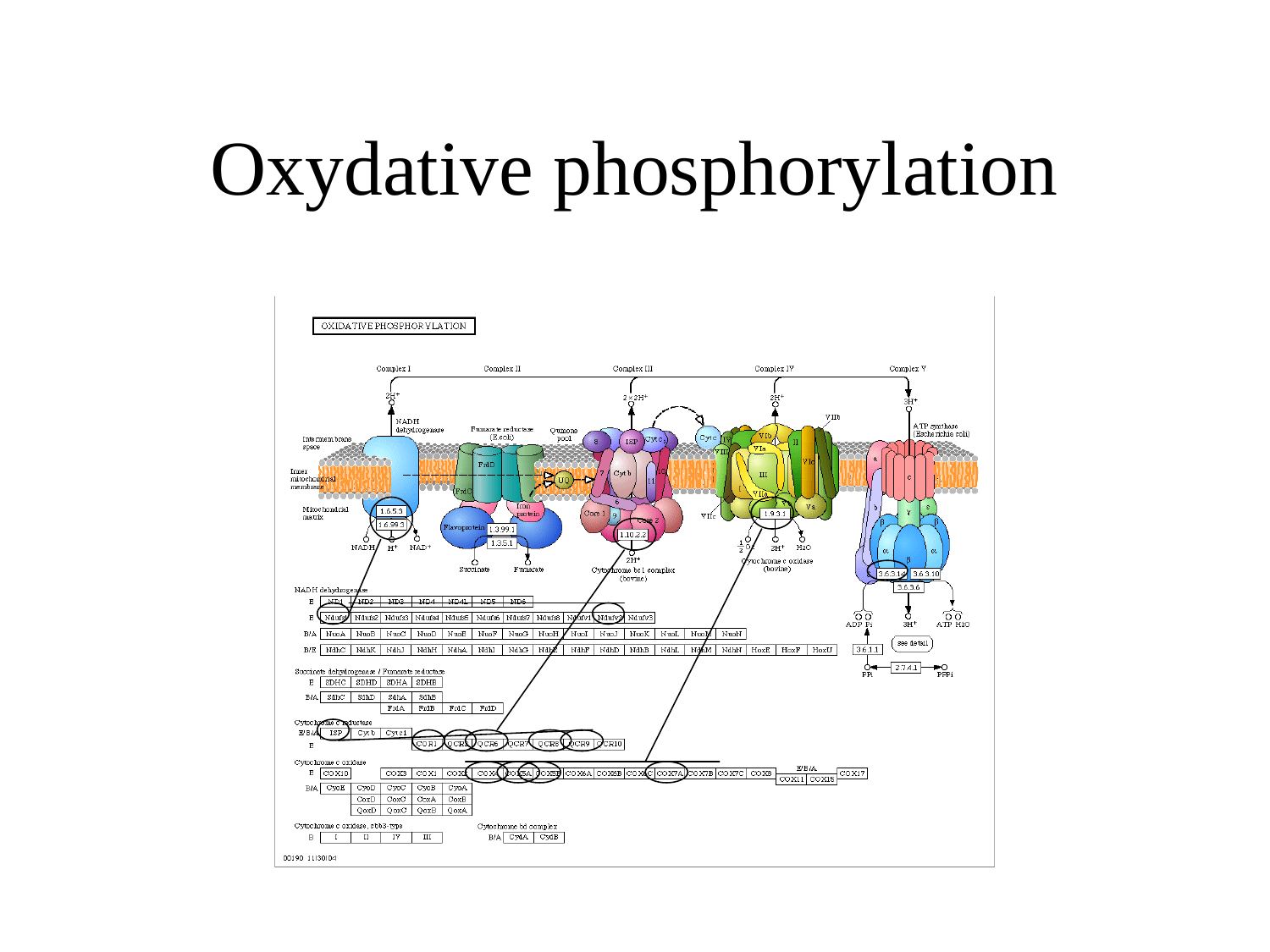

# Oxydative phosphorylation

## Slide 35
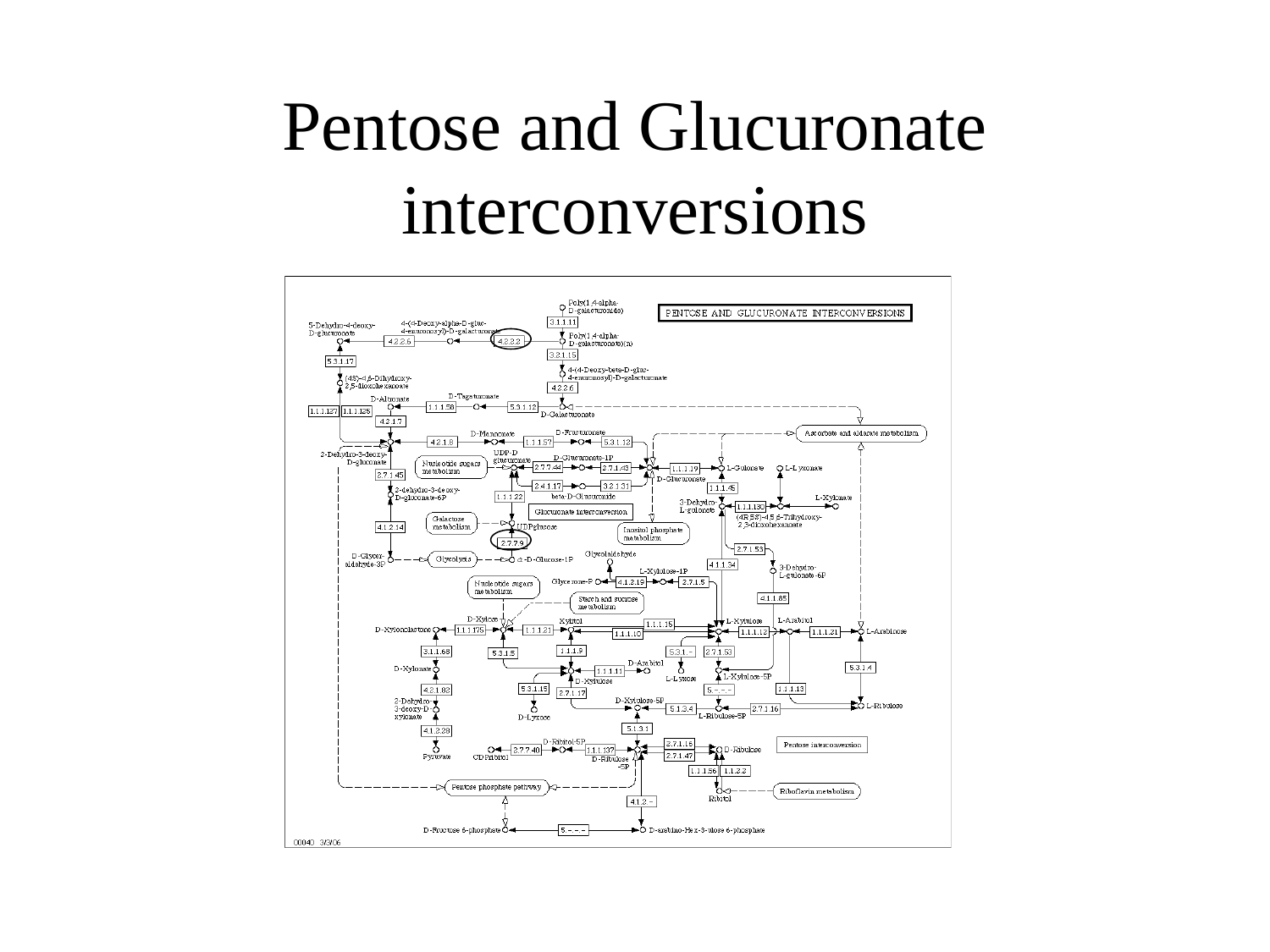

# Pentose and Glucuronate interconversions

## Slide 36
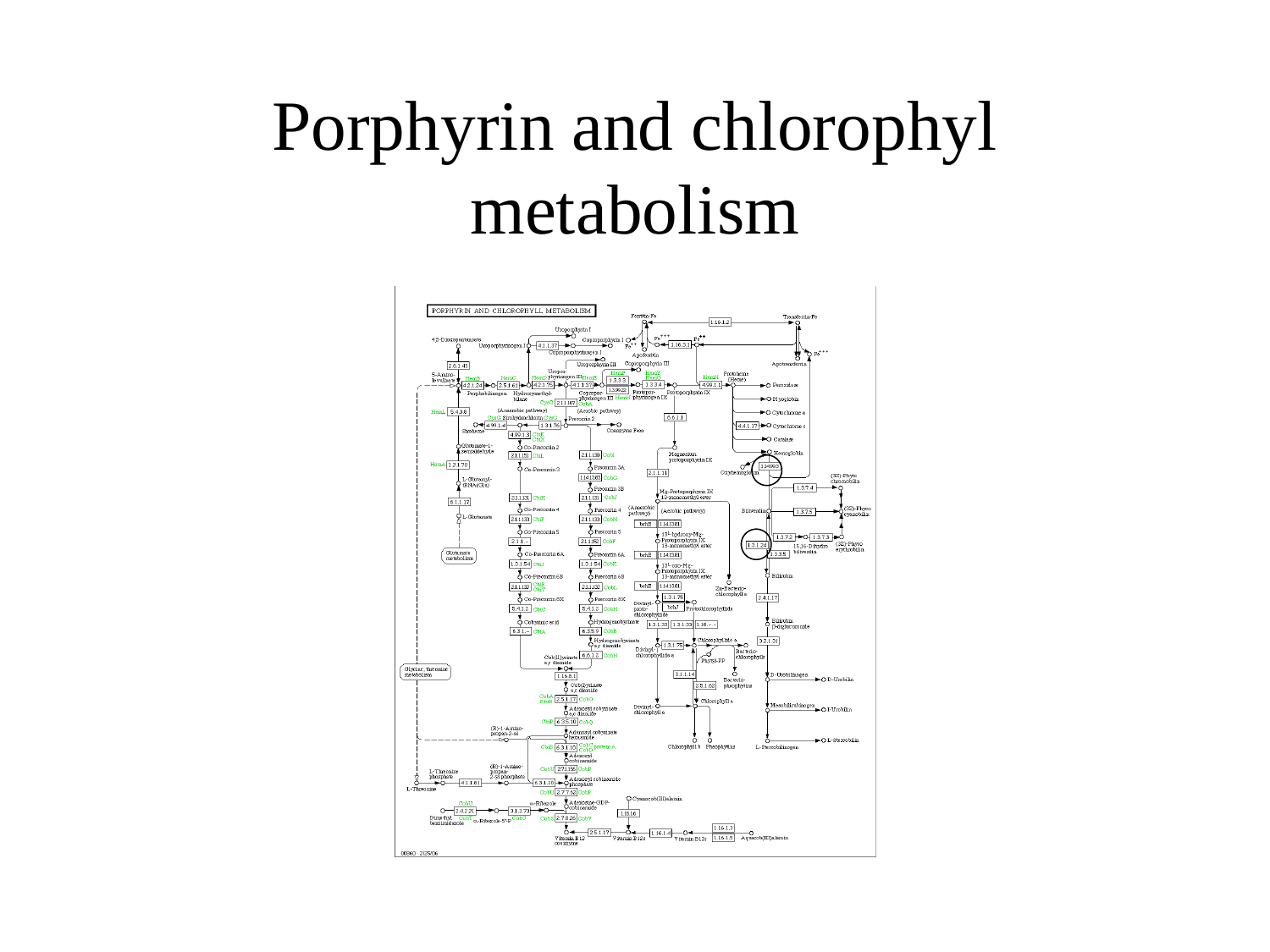

# Porphyrin and chlorophyl metabolism

## Slide 37
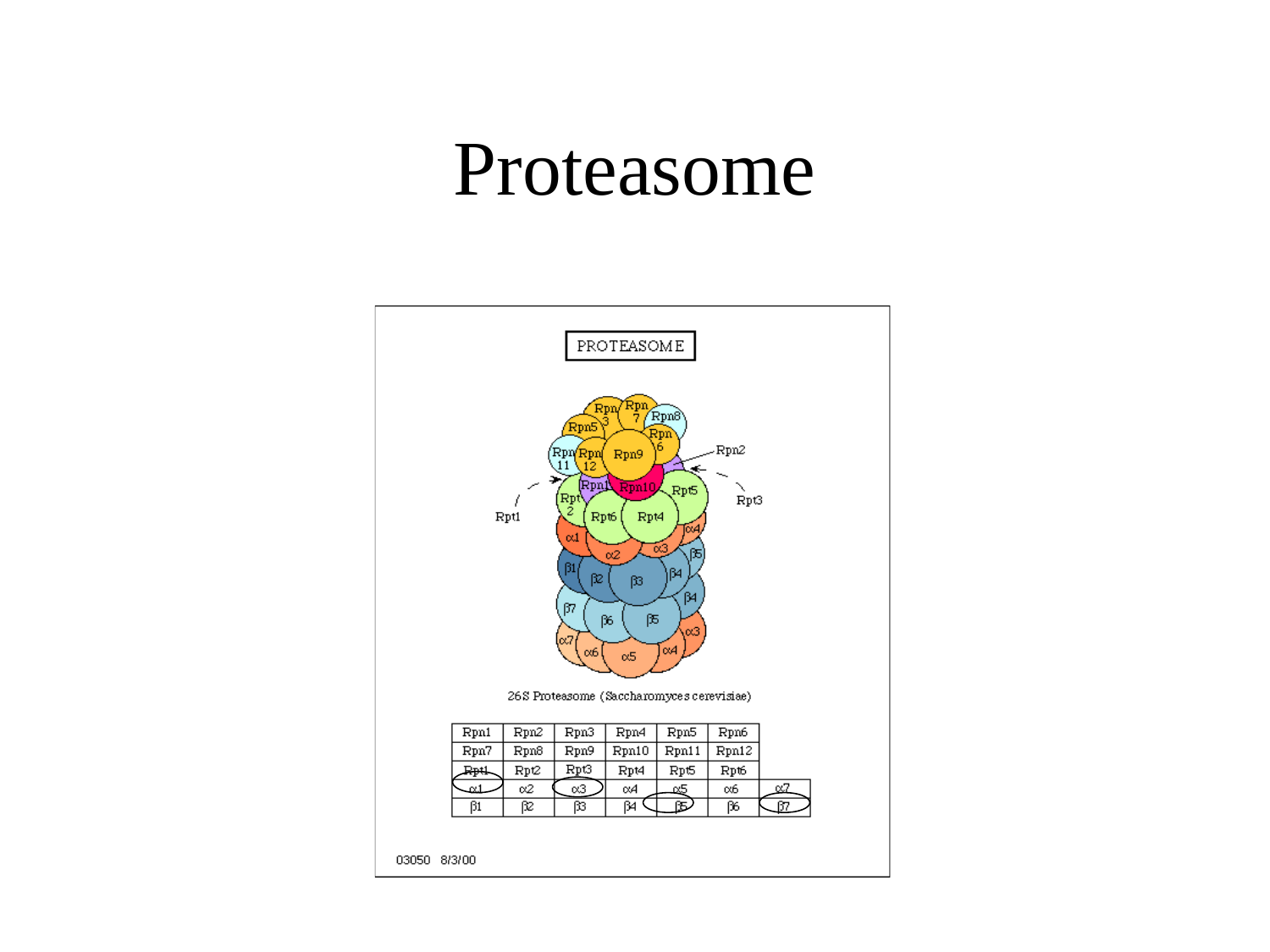

# Proteasome

## Slide 38
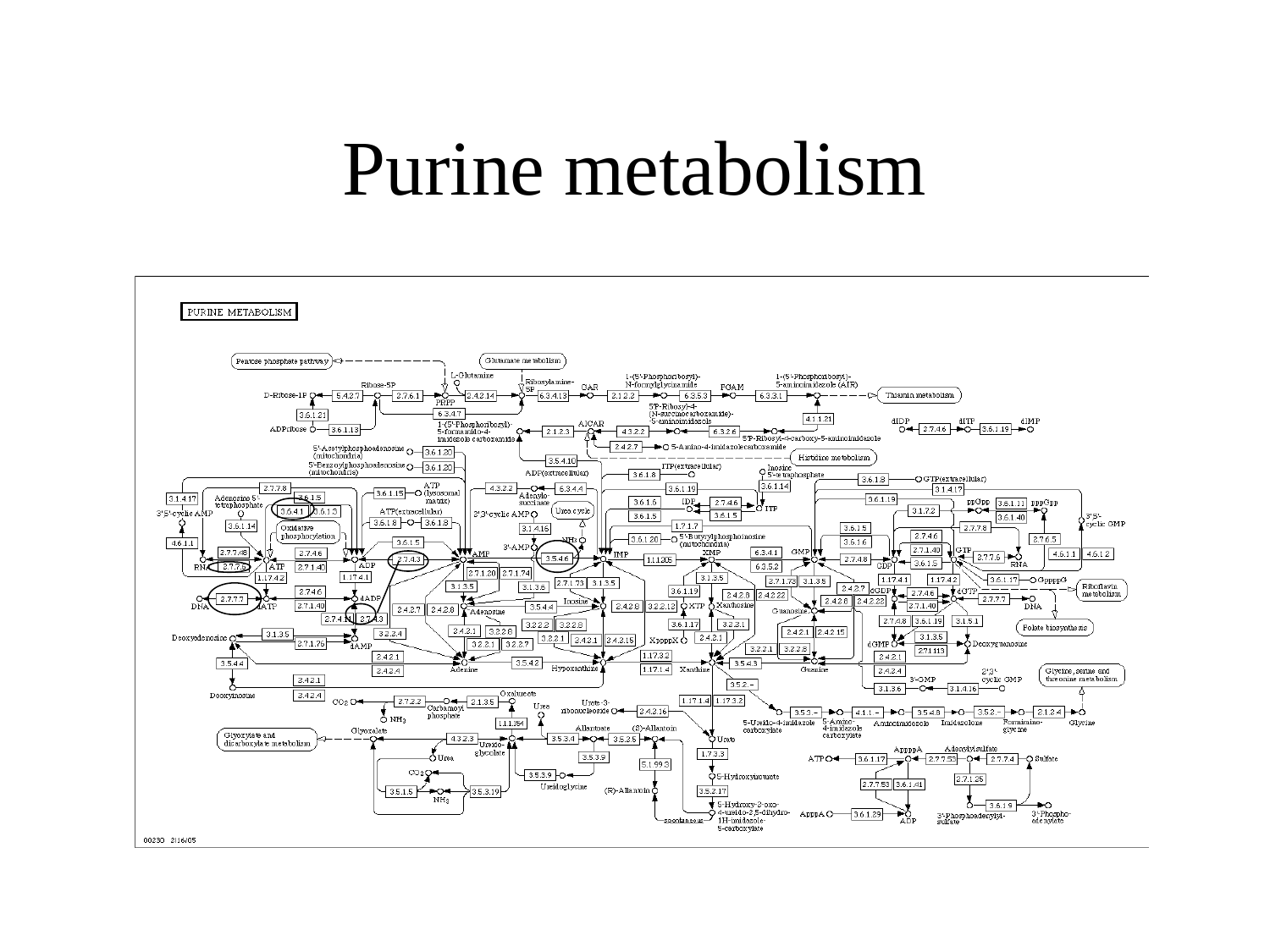

# Purine metabolism

## Slide 39
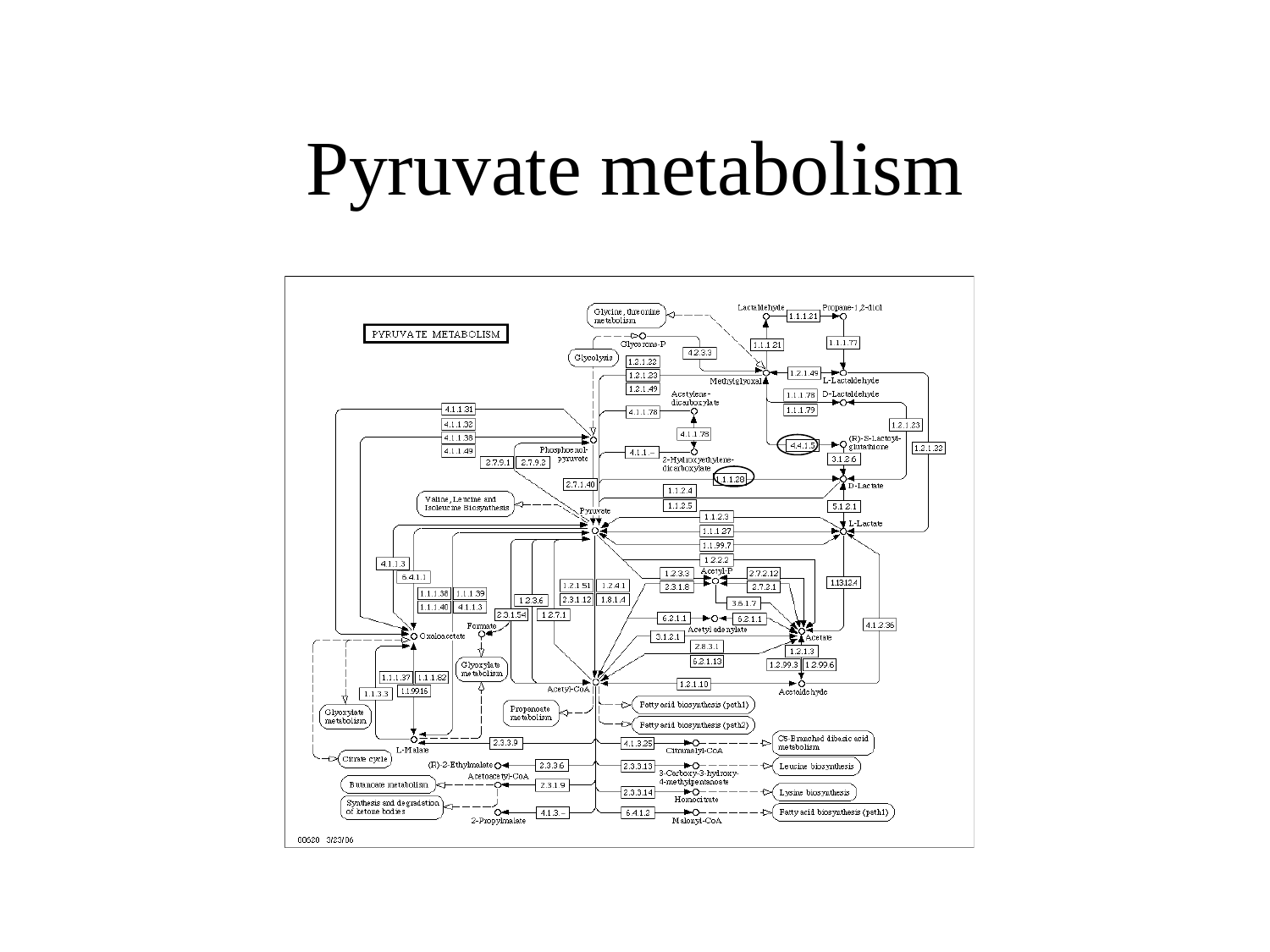

# Pyruvate metabolism

## Slide 40
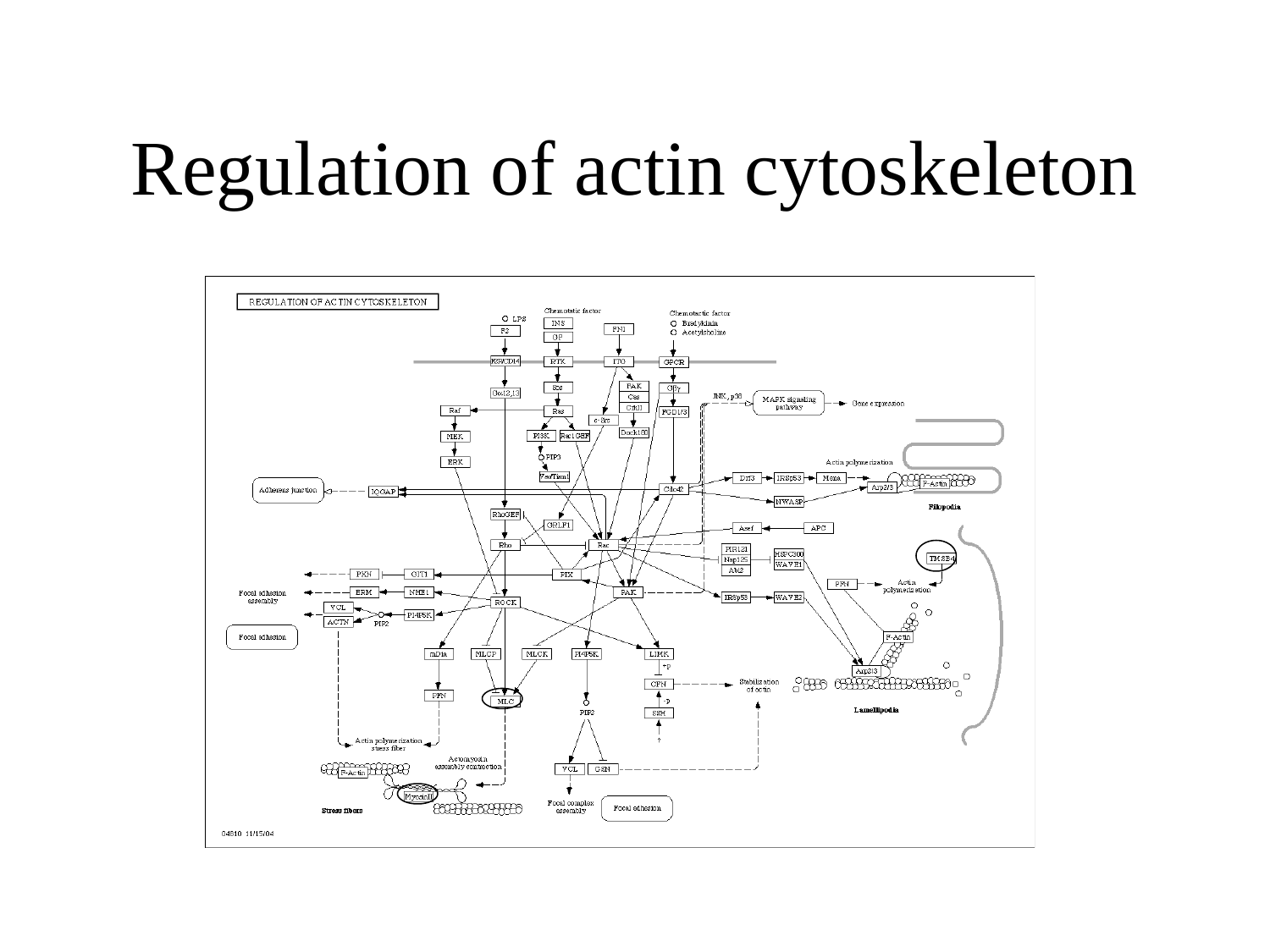

# Regulation of actin cytoskeleton

## Slide 41
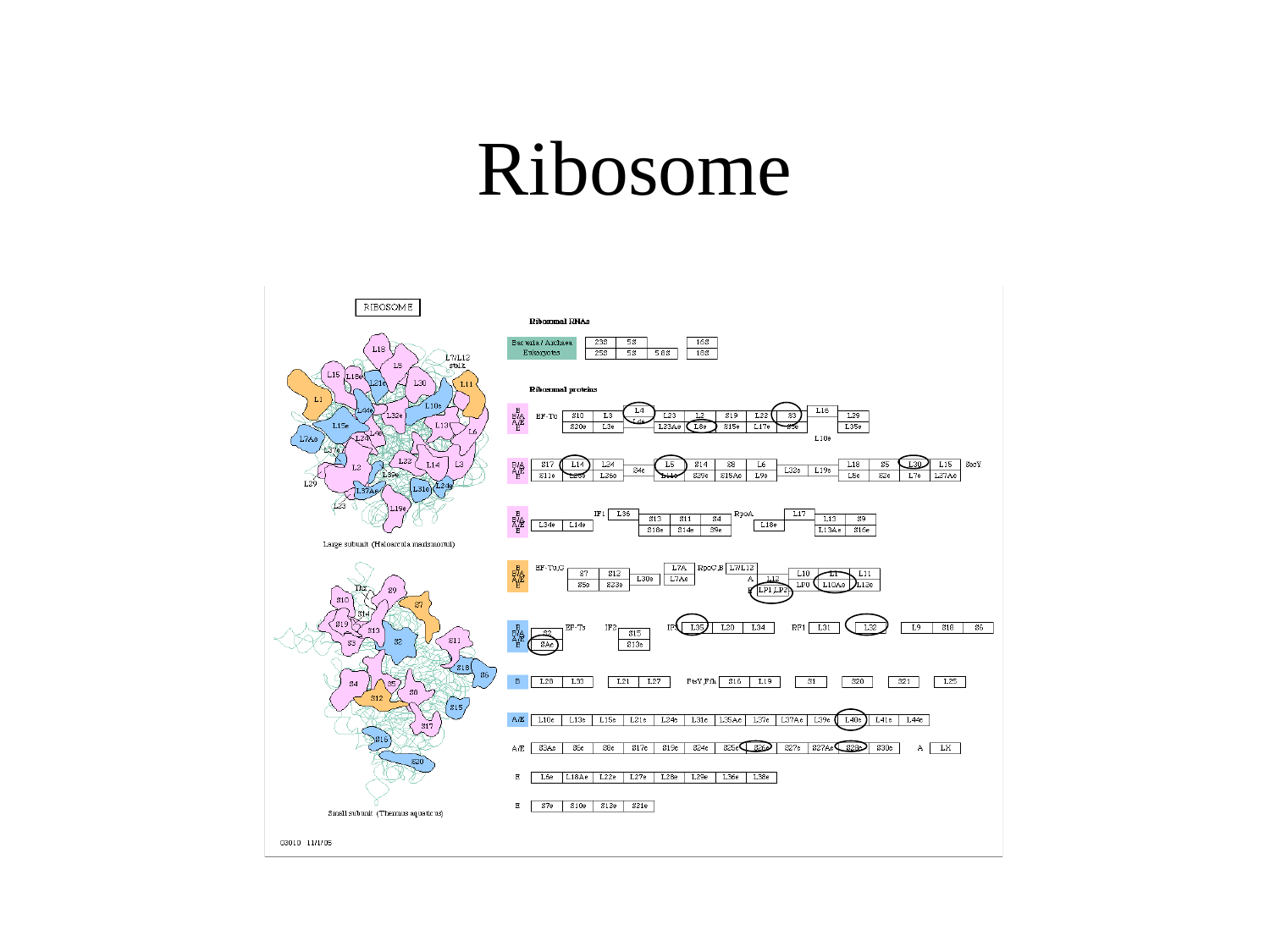

# Ribosome

## Slide 42
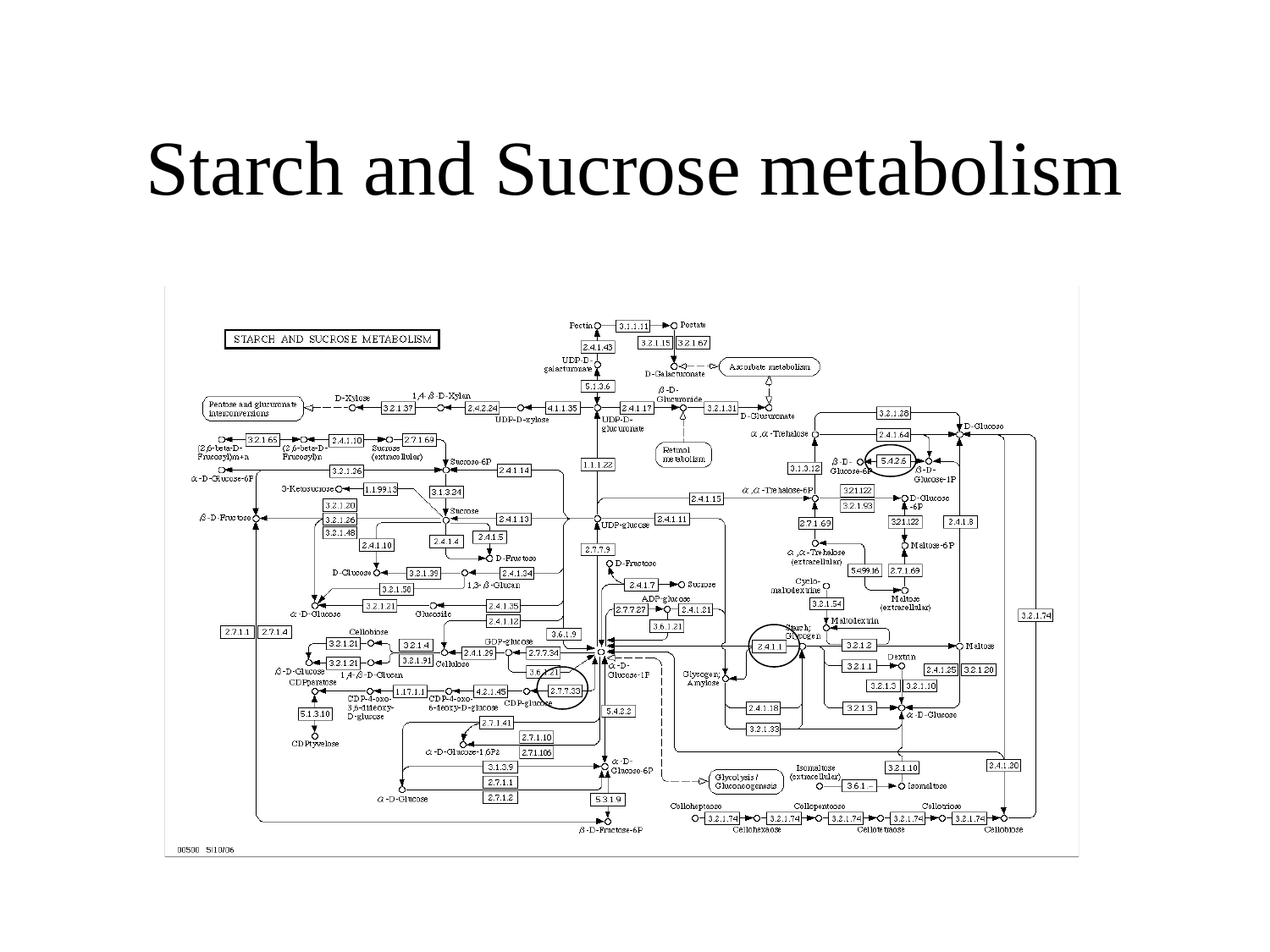

# Starch and Sucrose metabolism

## Slide 43
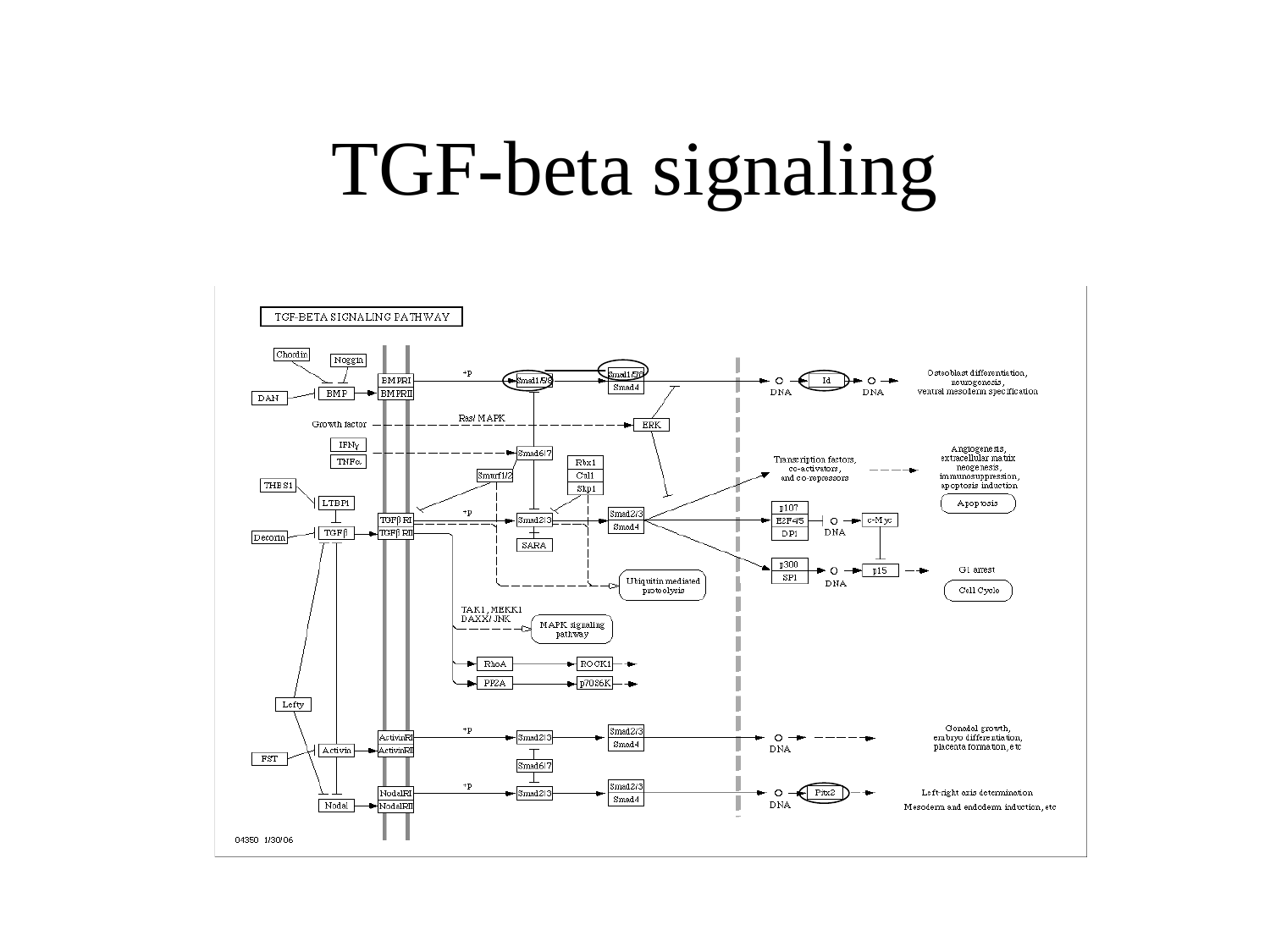

# TGF-beta signaling

## Slide 44
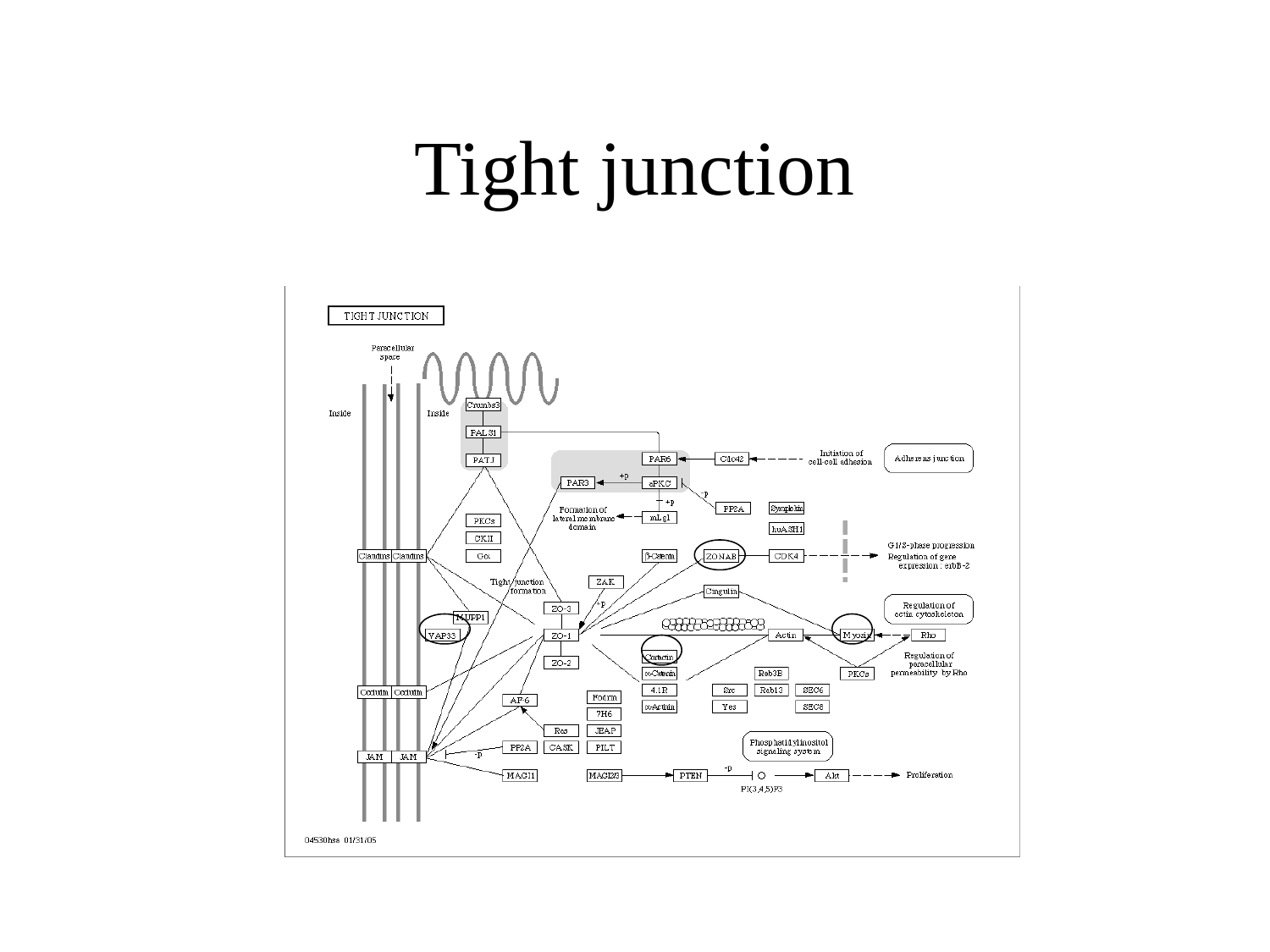

# Tight junction

## Slide 45
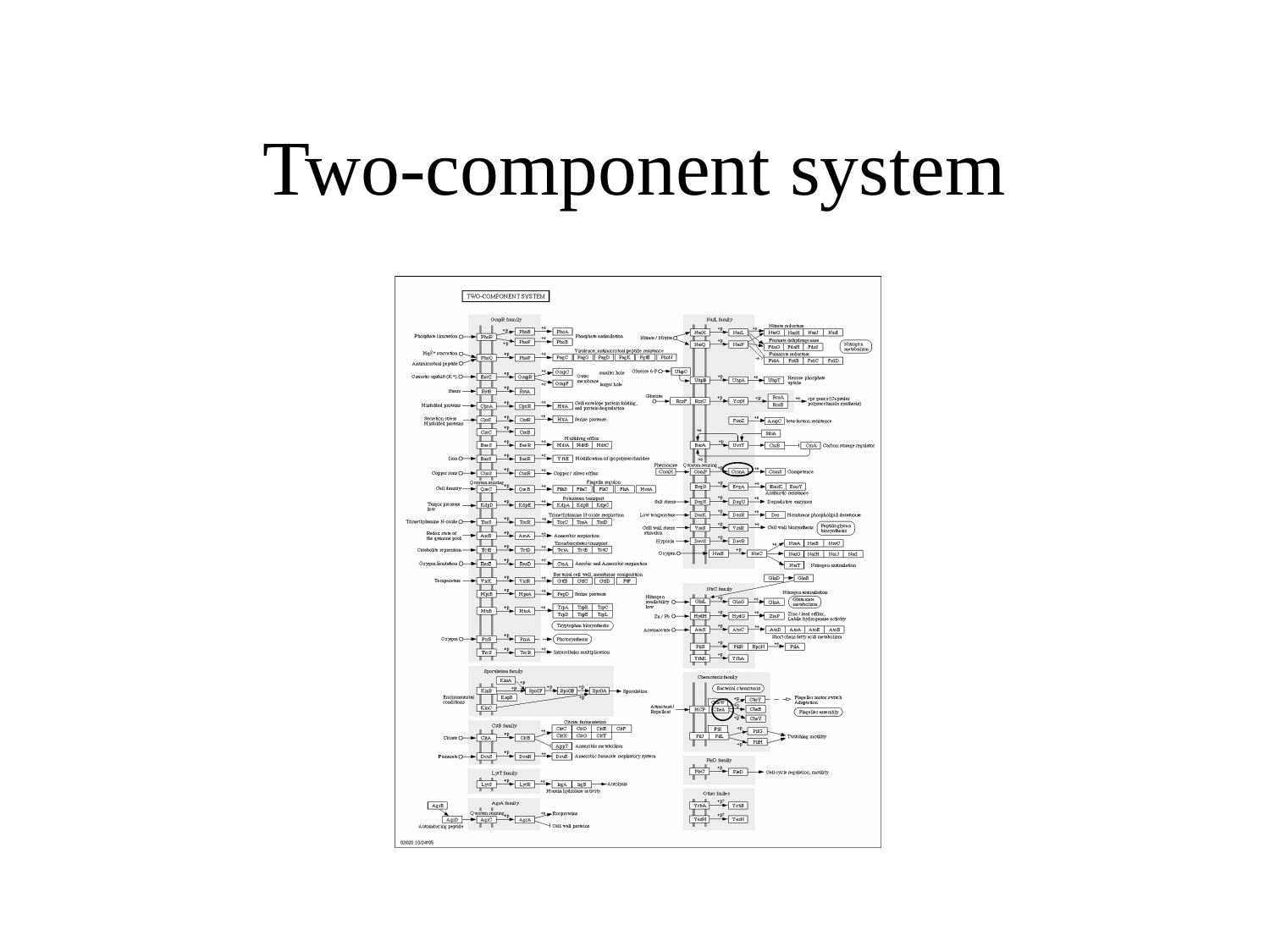

# Two-component system

## Slide 46
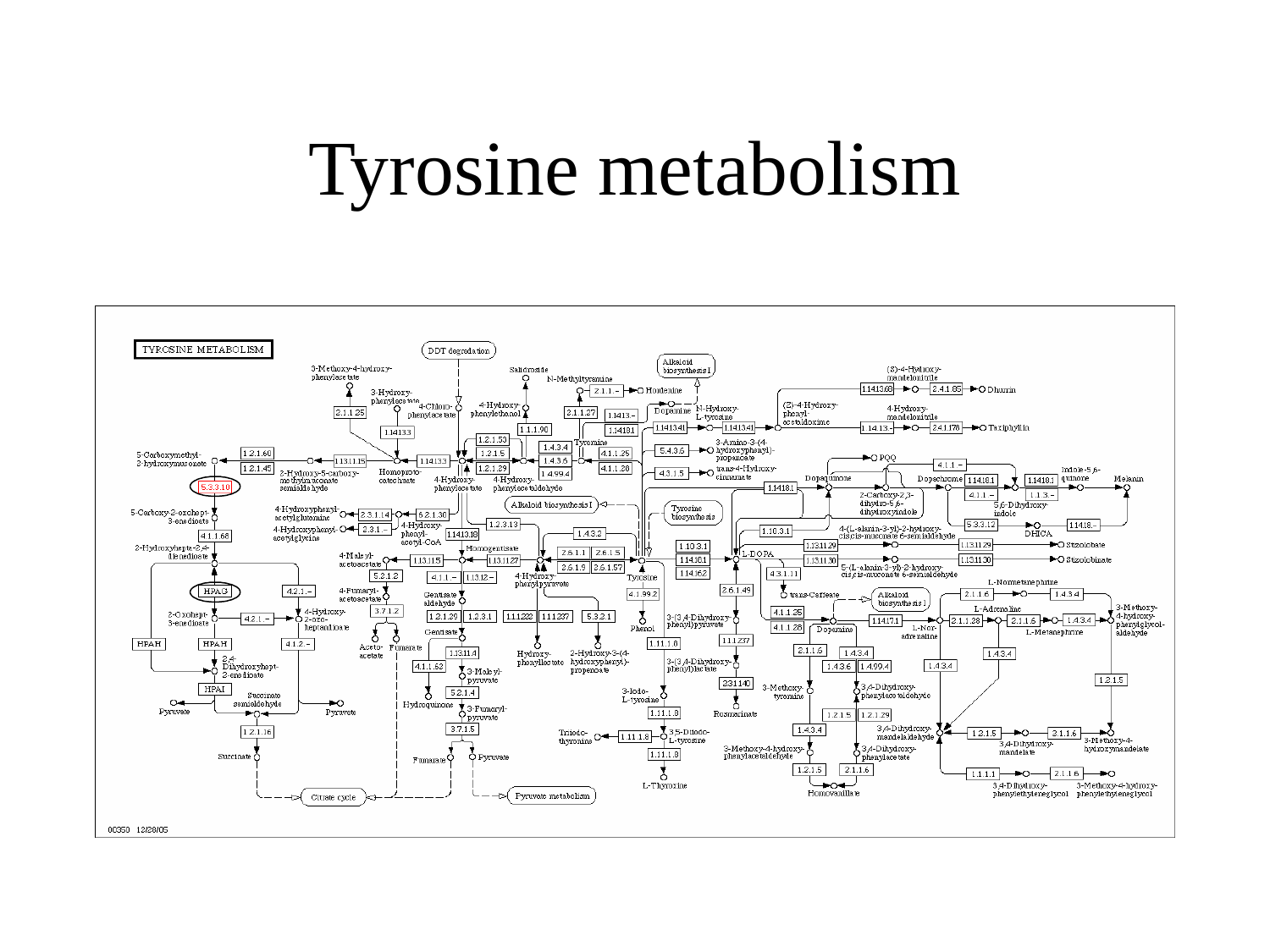

# Tyrosine metabolism

## Slide 47
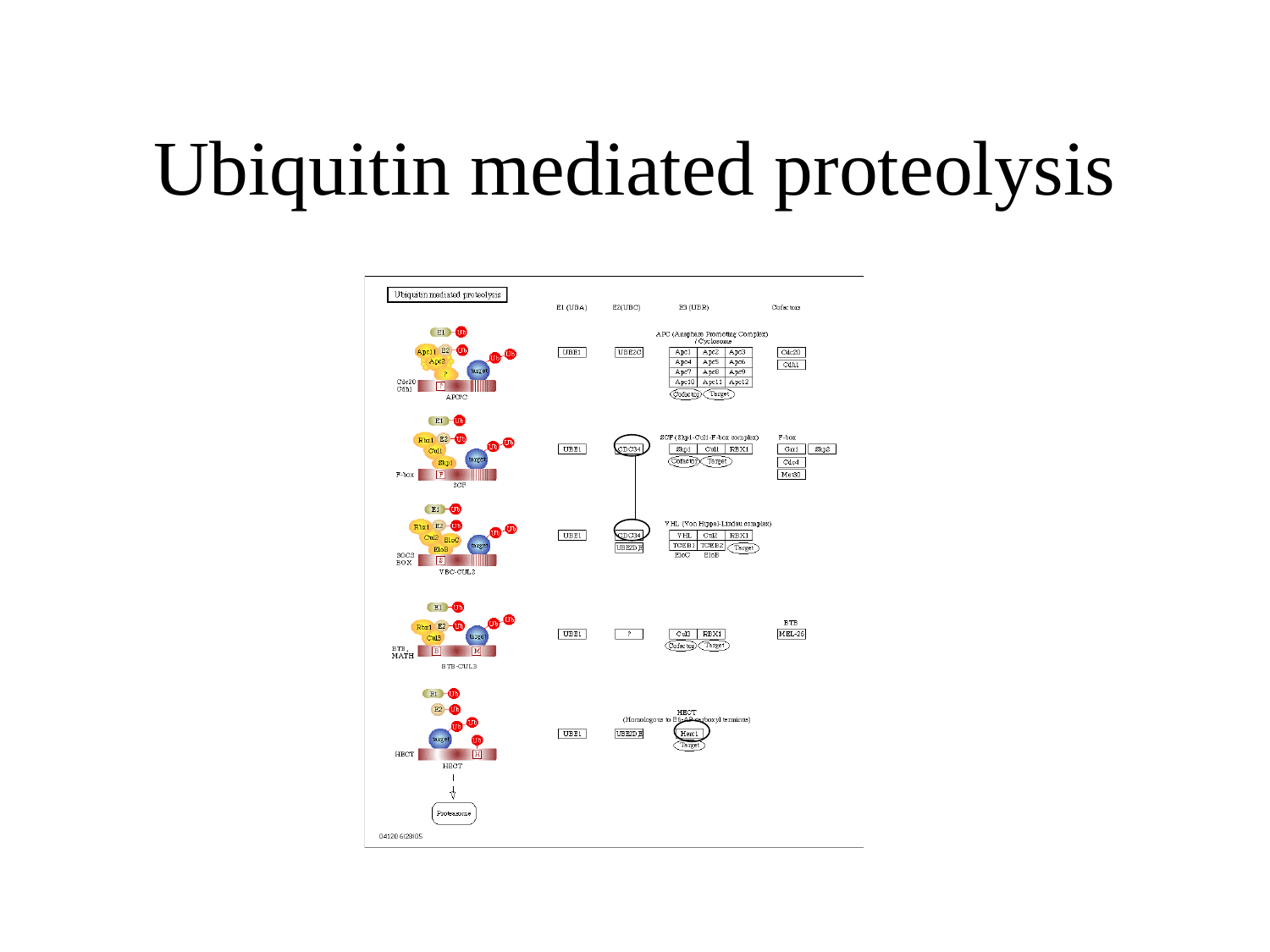

# Ubiquitin mediated proteolysis

## Slide 48
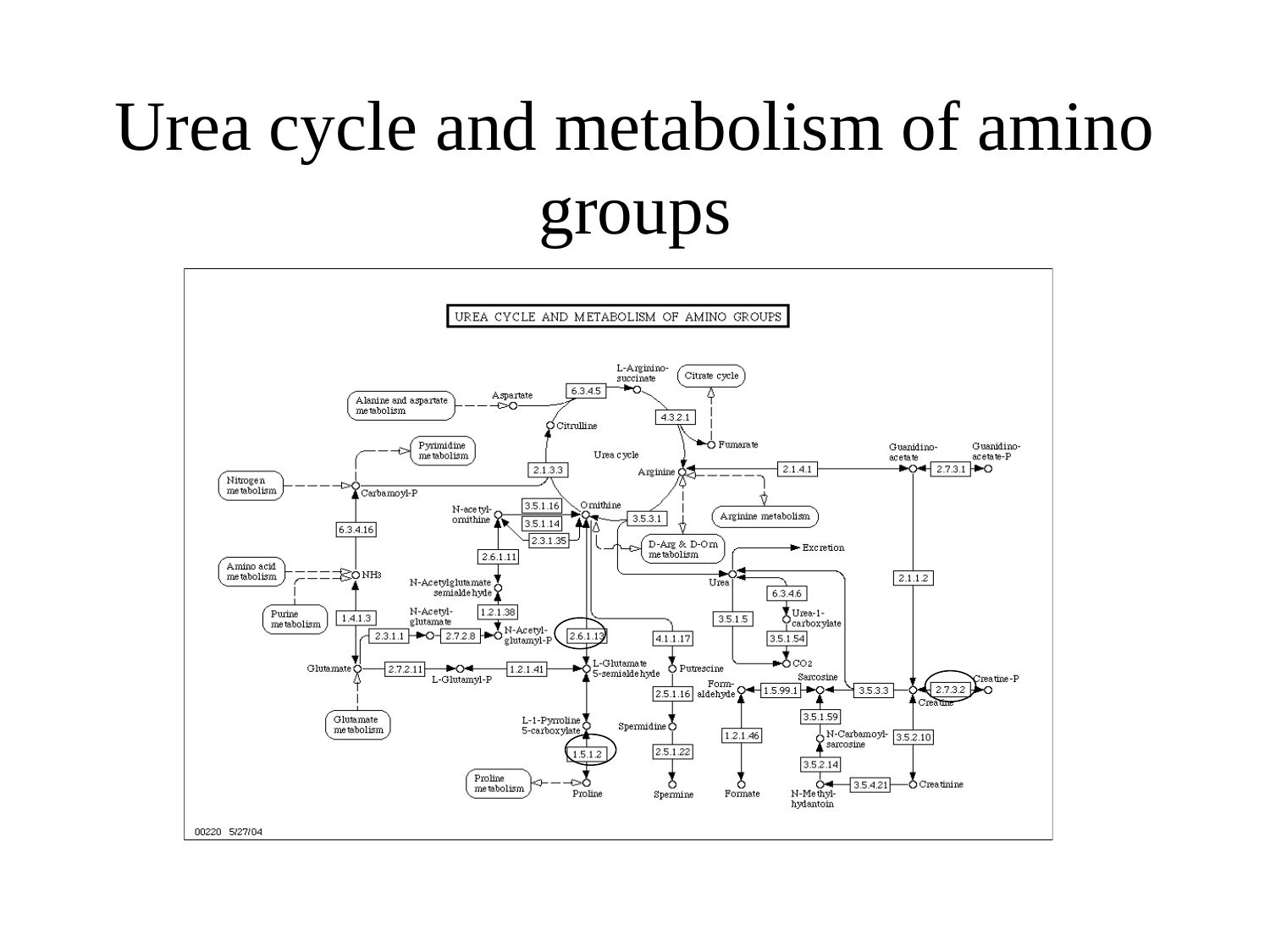

# Urea cycle and metabolism of amino groups

## Slide 49
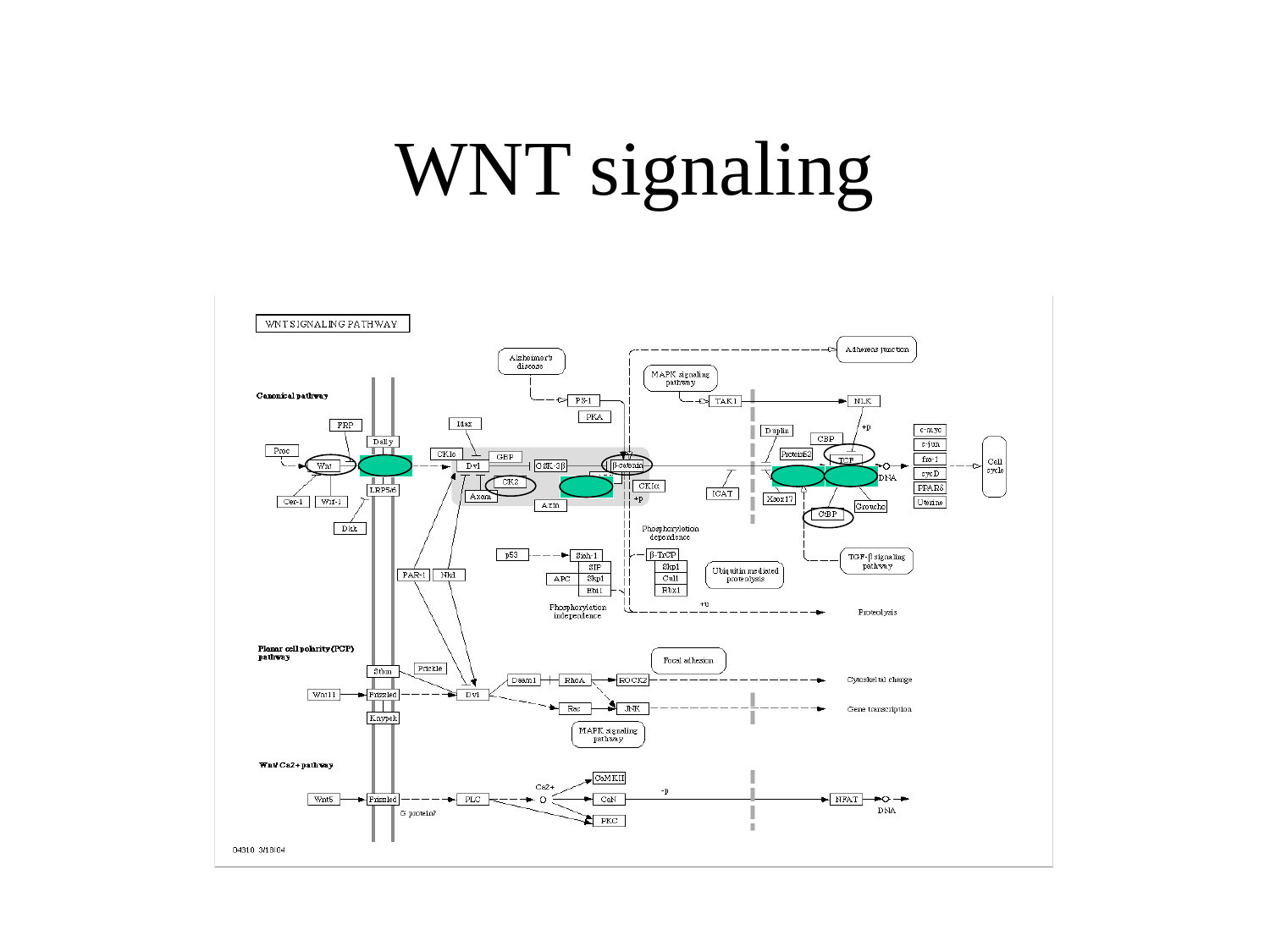

# WNT signaling
