# Supplementary material for: Biochemical pathways analysis of microarray results: regulation of myogenesis in pigs
Source: BMC Dev Biol. 2007 Jun 13;7:66. doi: 10.1186/1471-213X-7-66 (PMC1919358; doi:10.1186/1471-213X-7-66)

## Slide 1
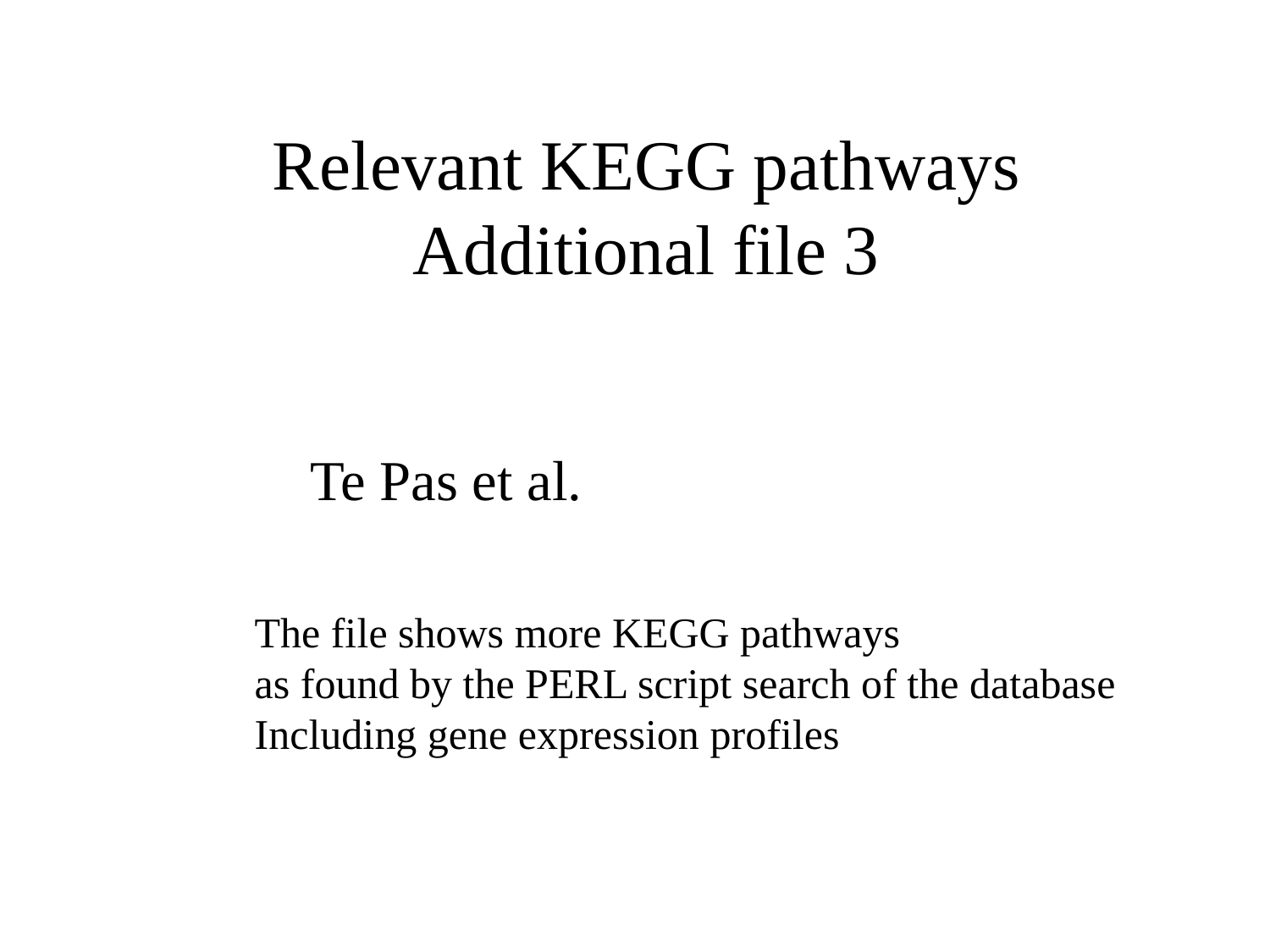

# Relevant KEGG pathways Additional file 3
Te Pas et al.
The file shows more KEGG pathways
as found by the PERL script search of the database
Including gene expression profiles

## Slide 2
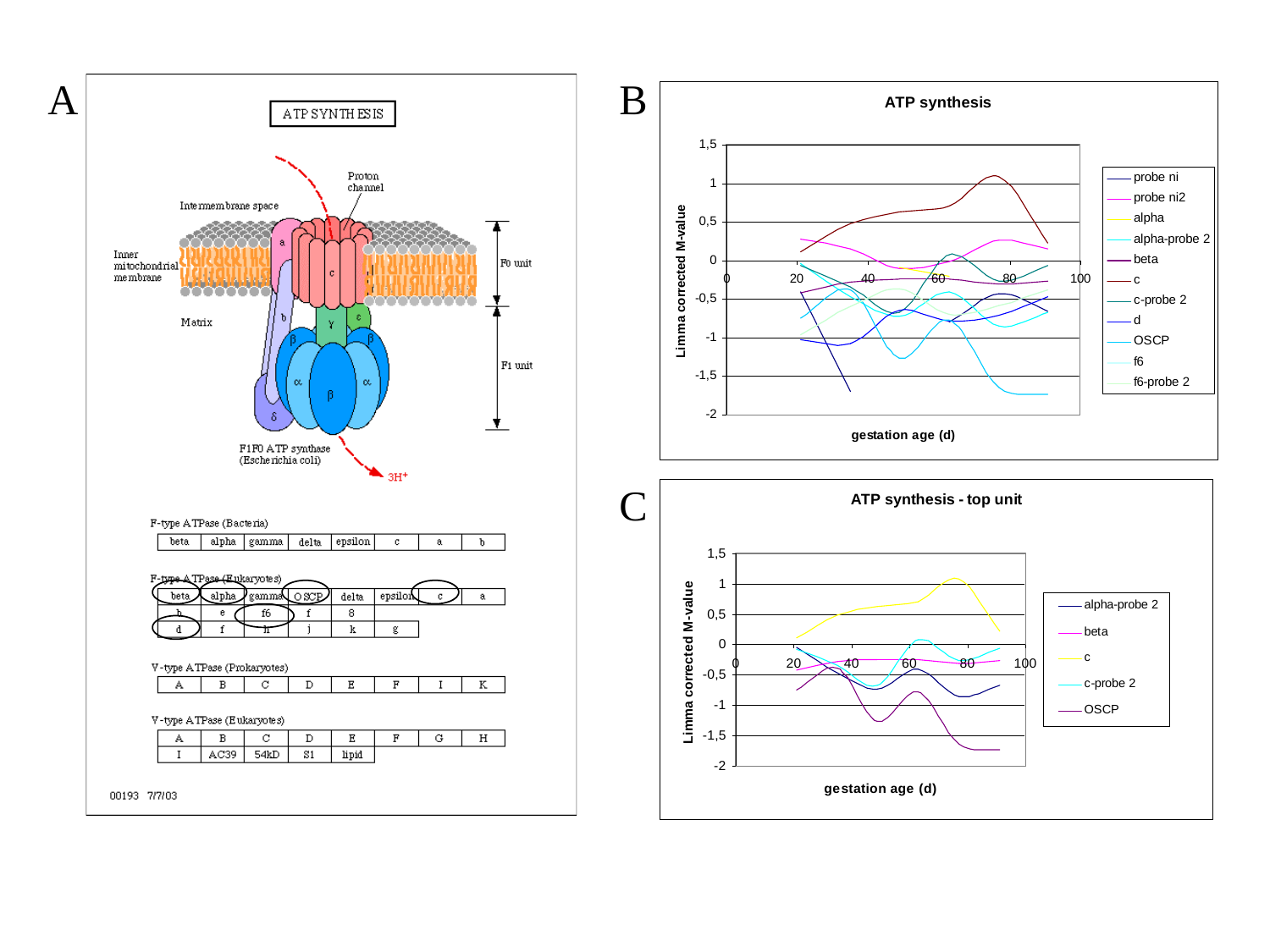

A				 B
				 C

## Slide 3
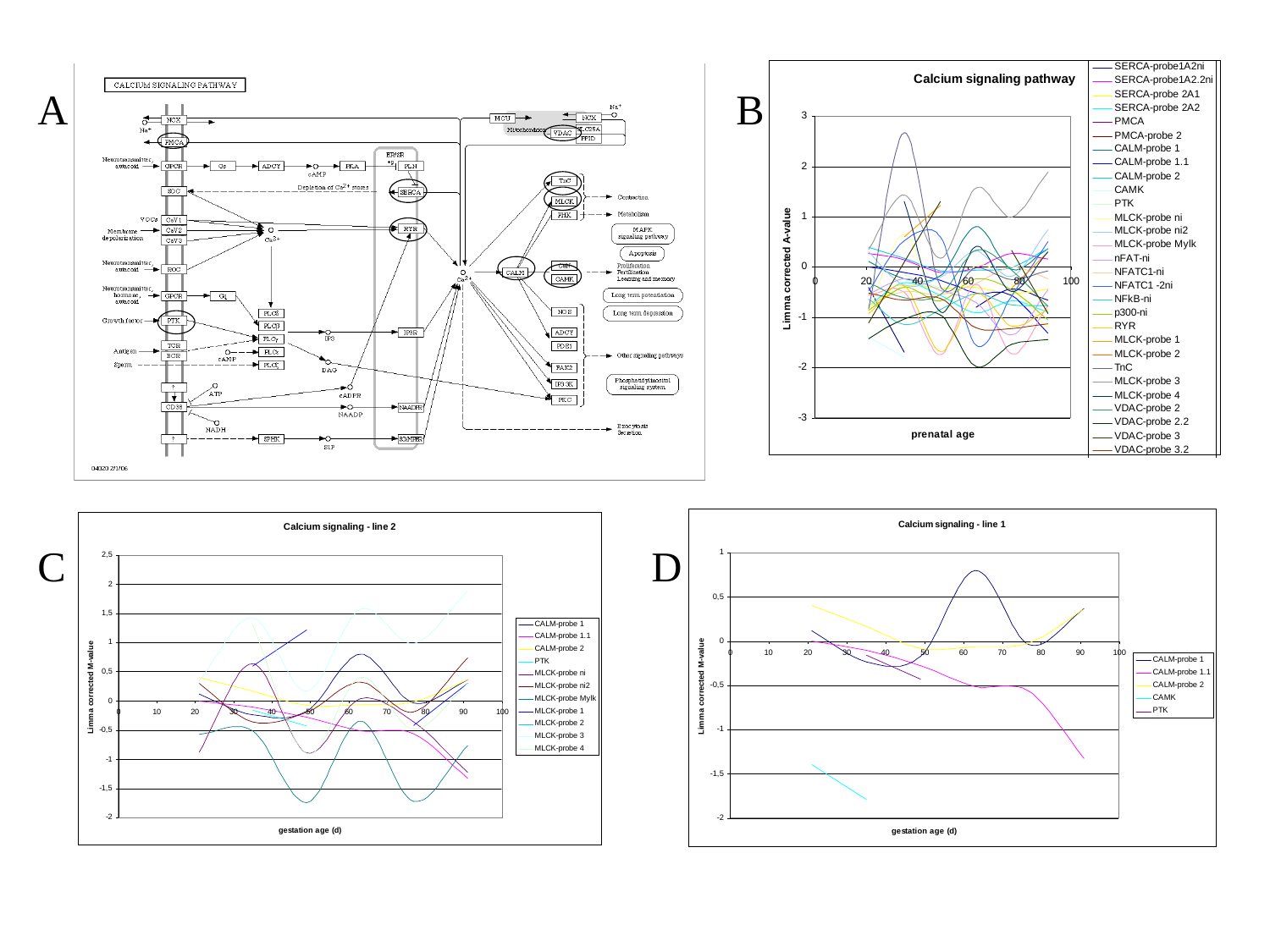

A					 B
C				 D

## Slide 4
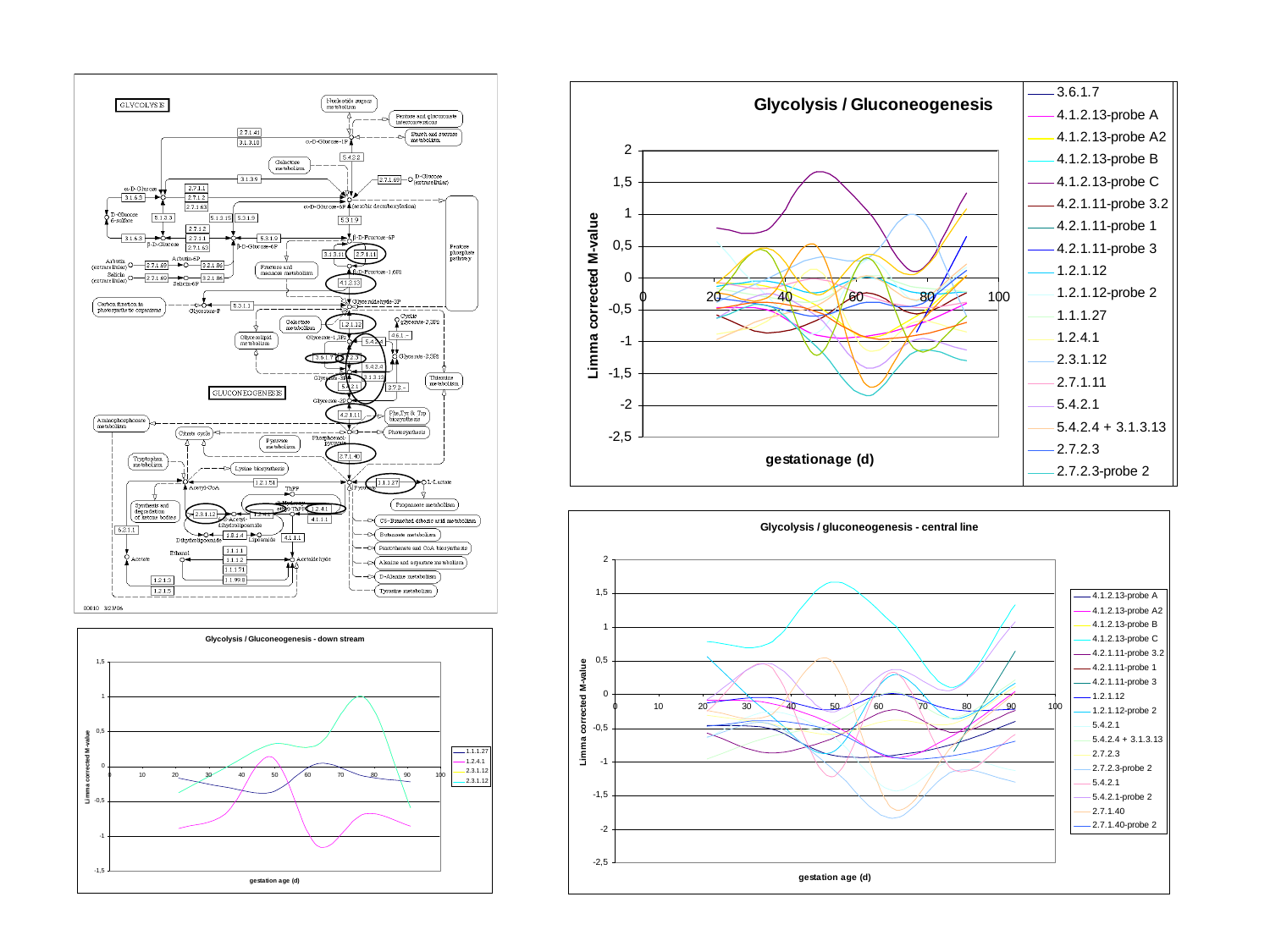

## Slide 5
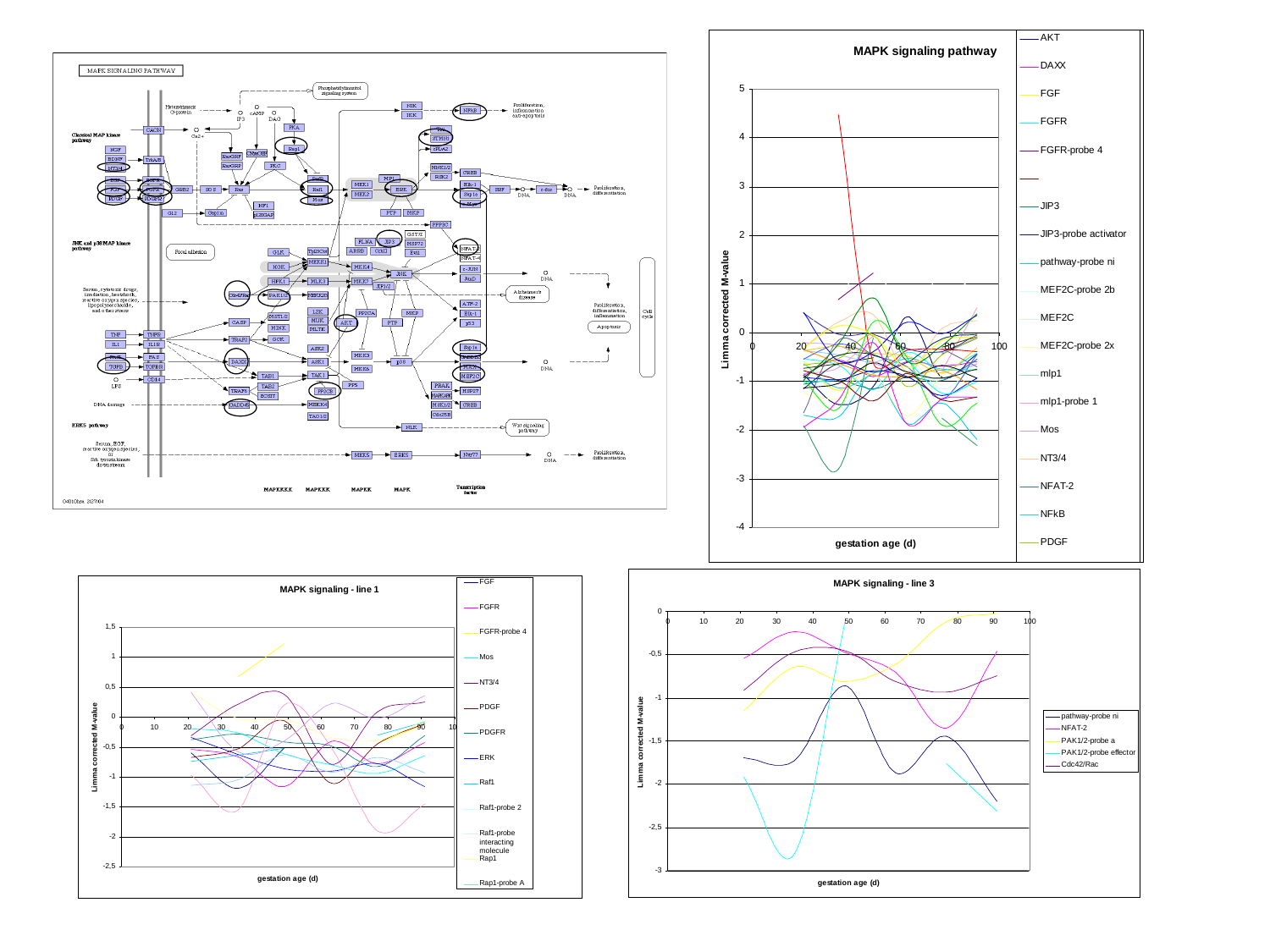

## Slide 6
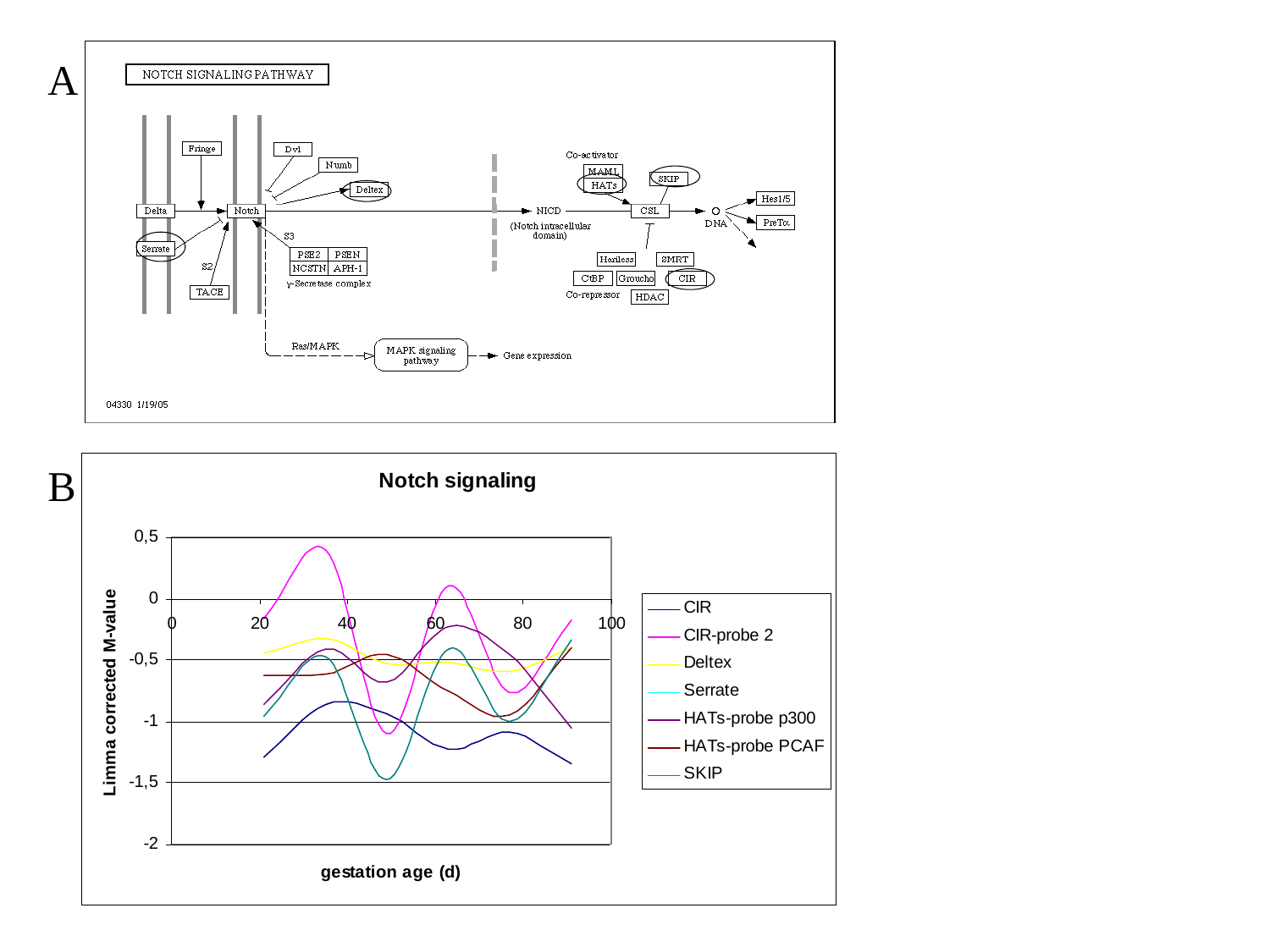

A
B

## Slide 7
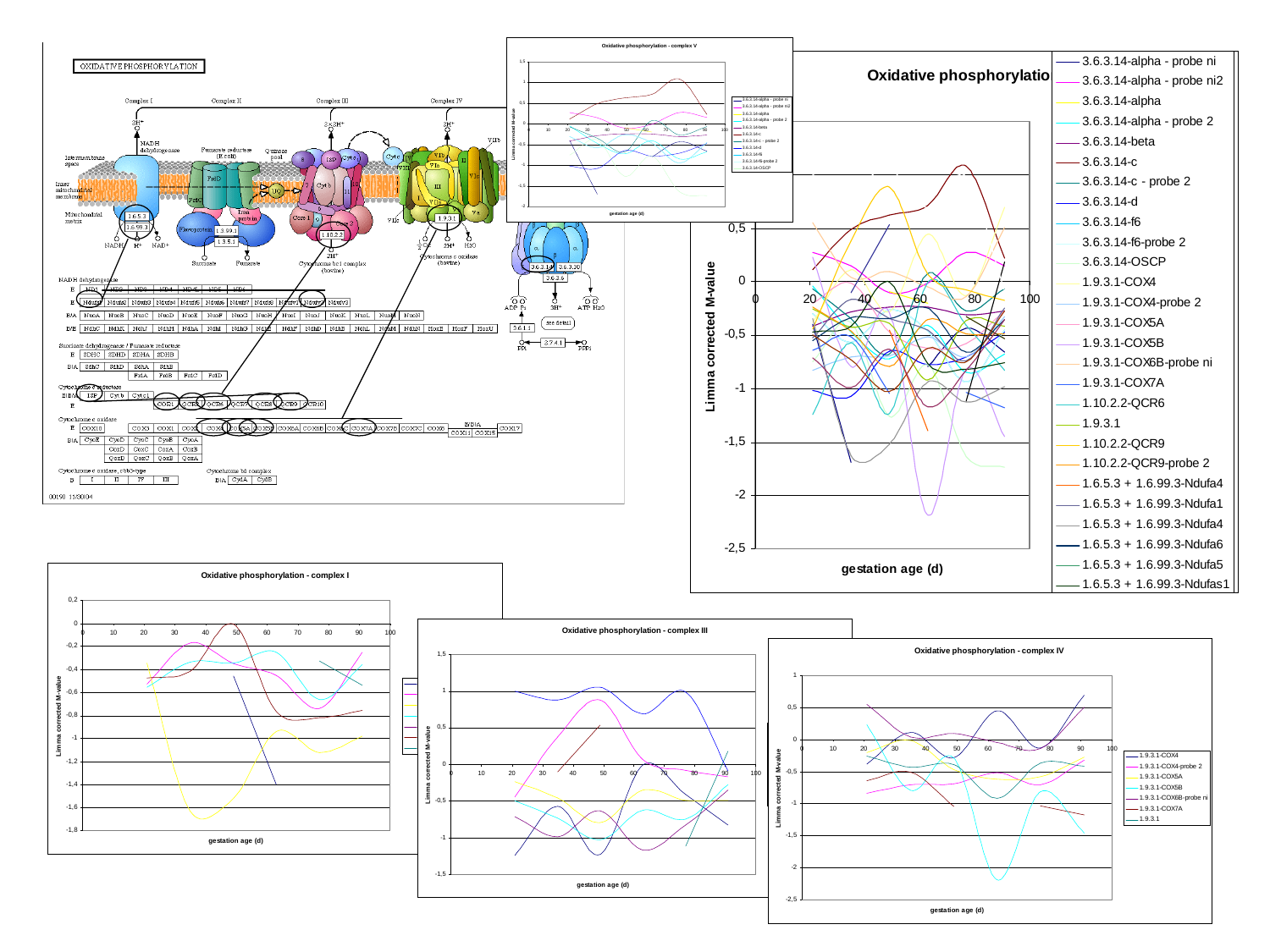

## Slide 8
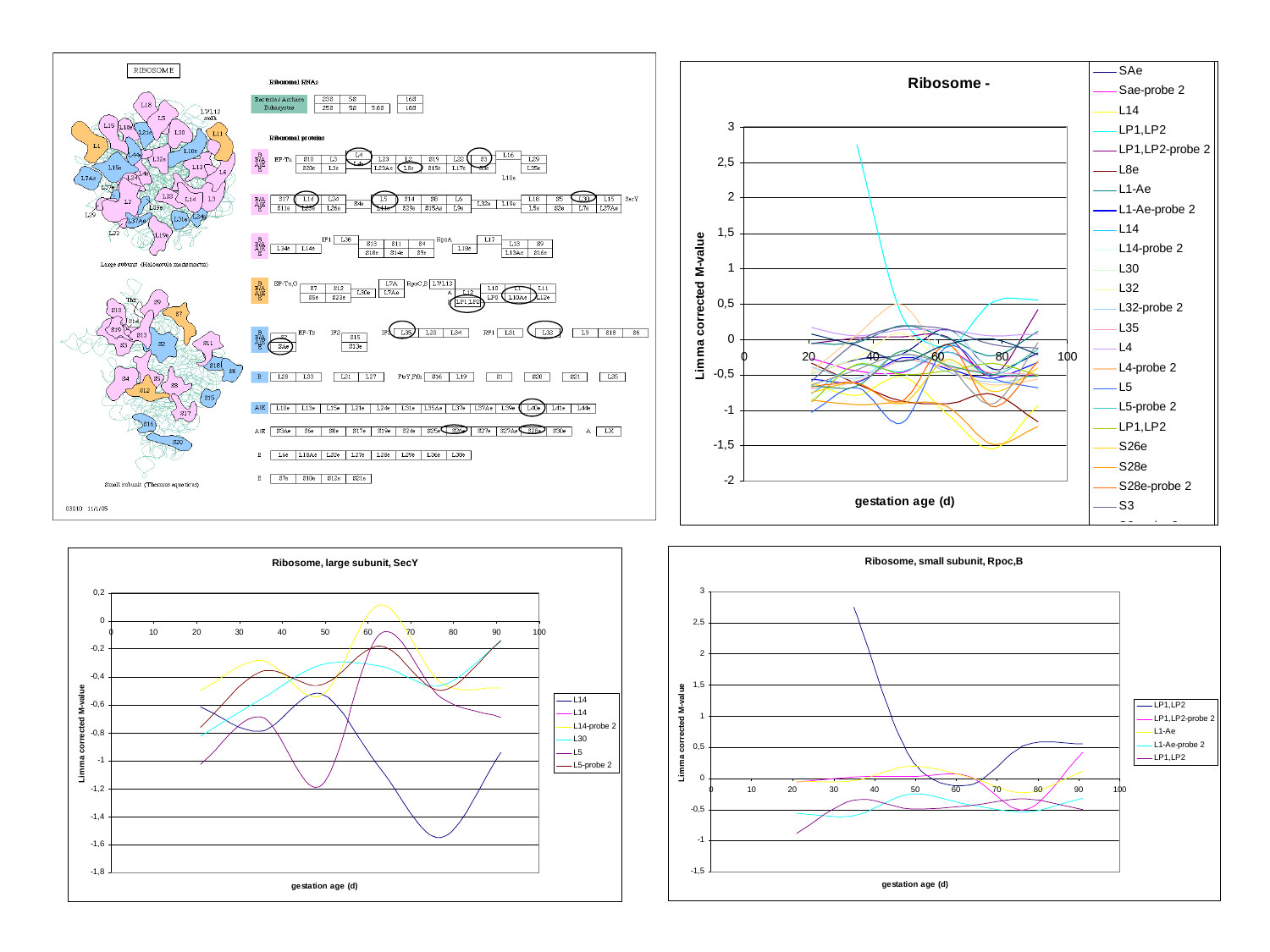

## Slide 9
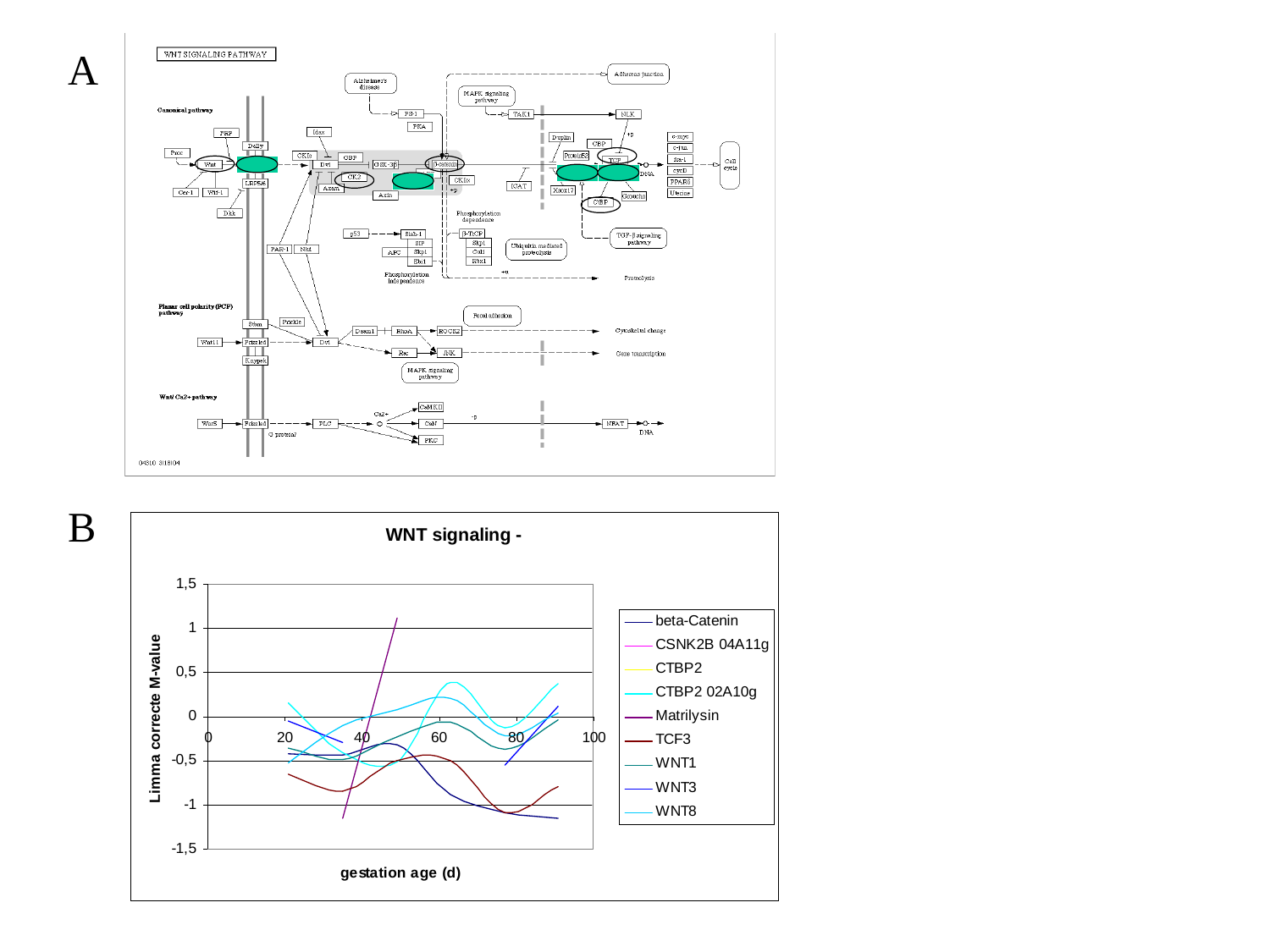

A
B

## Slide 10
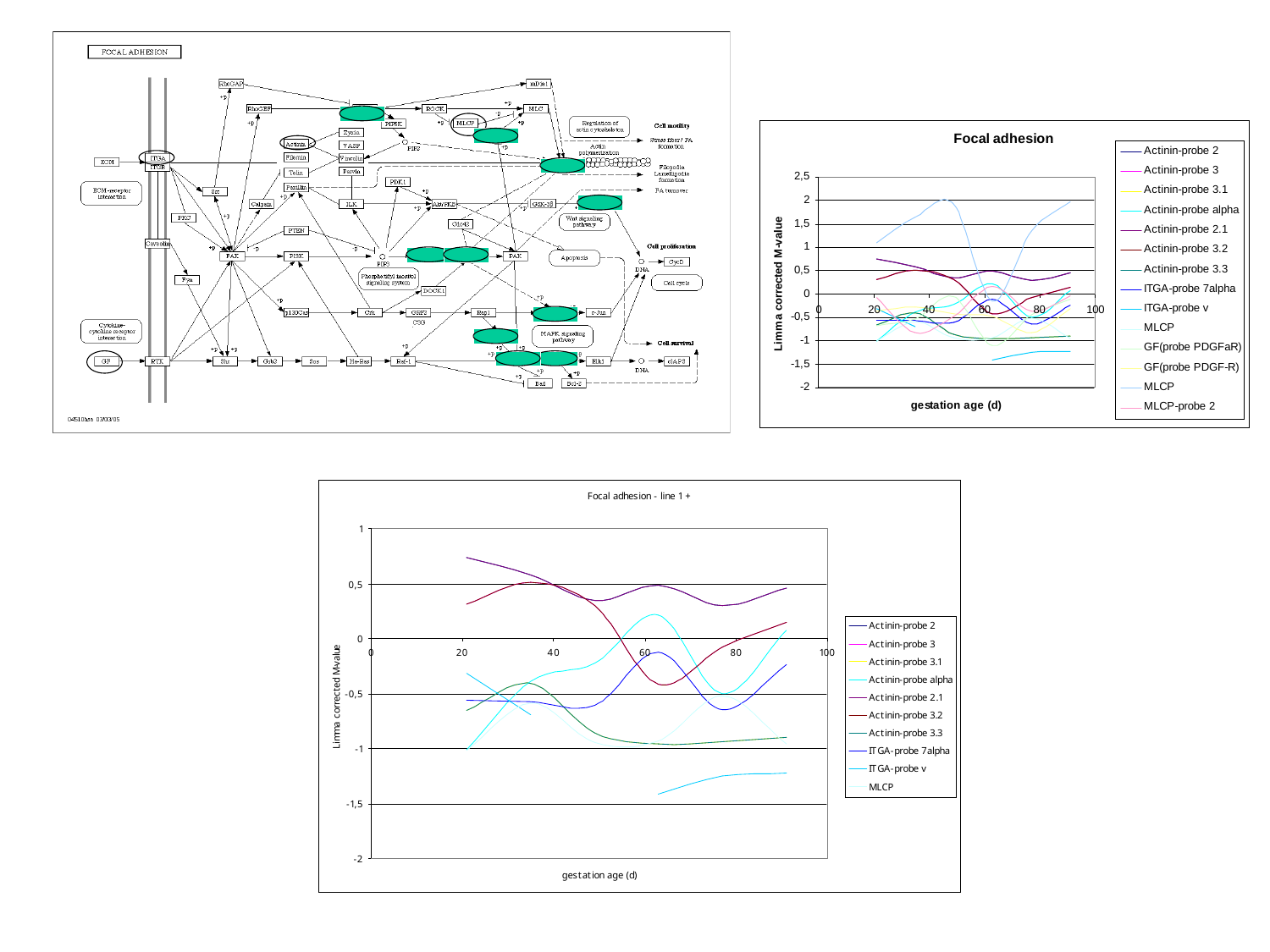

## Slide 11
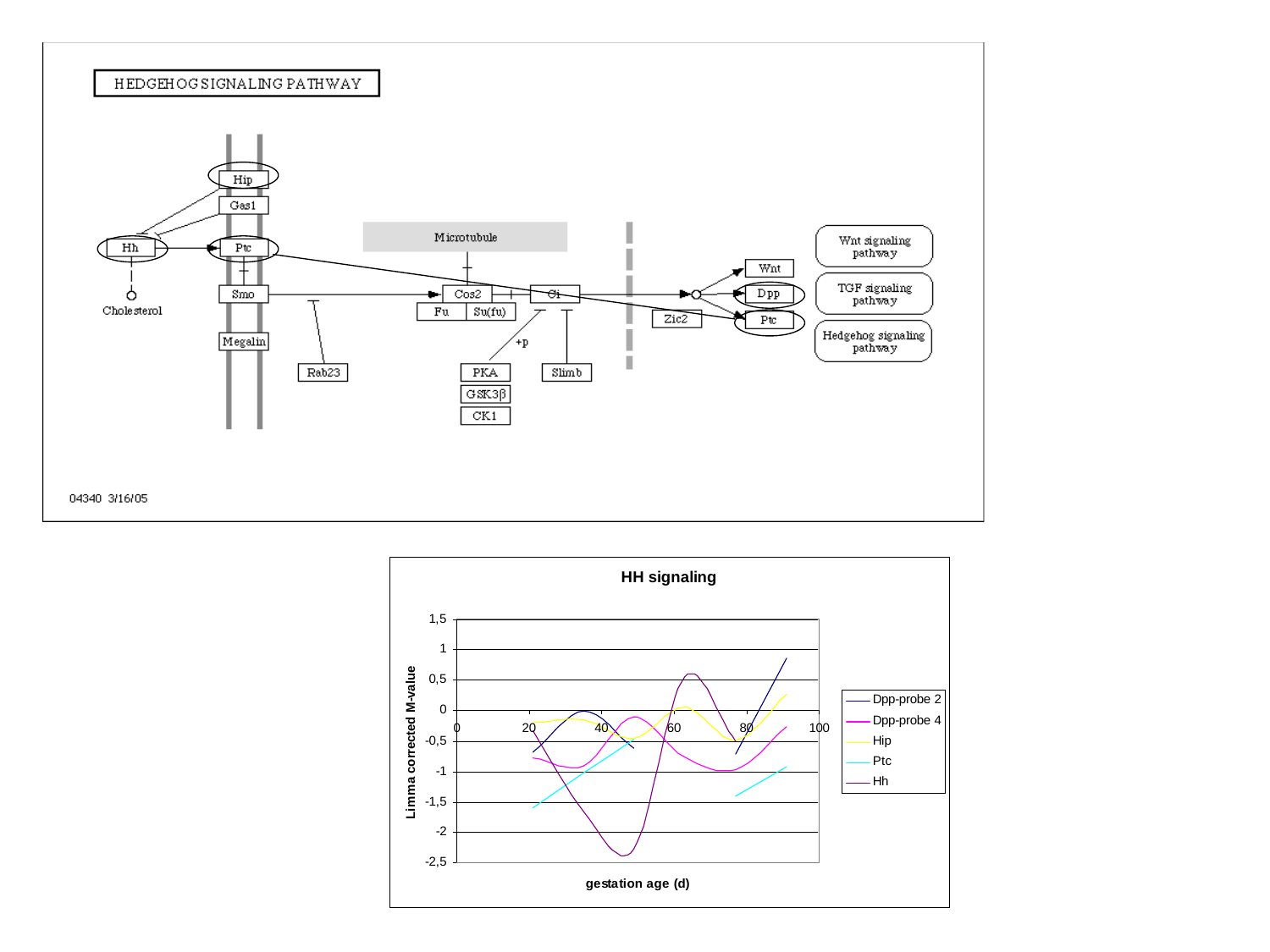

## Slide 12
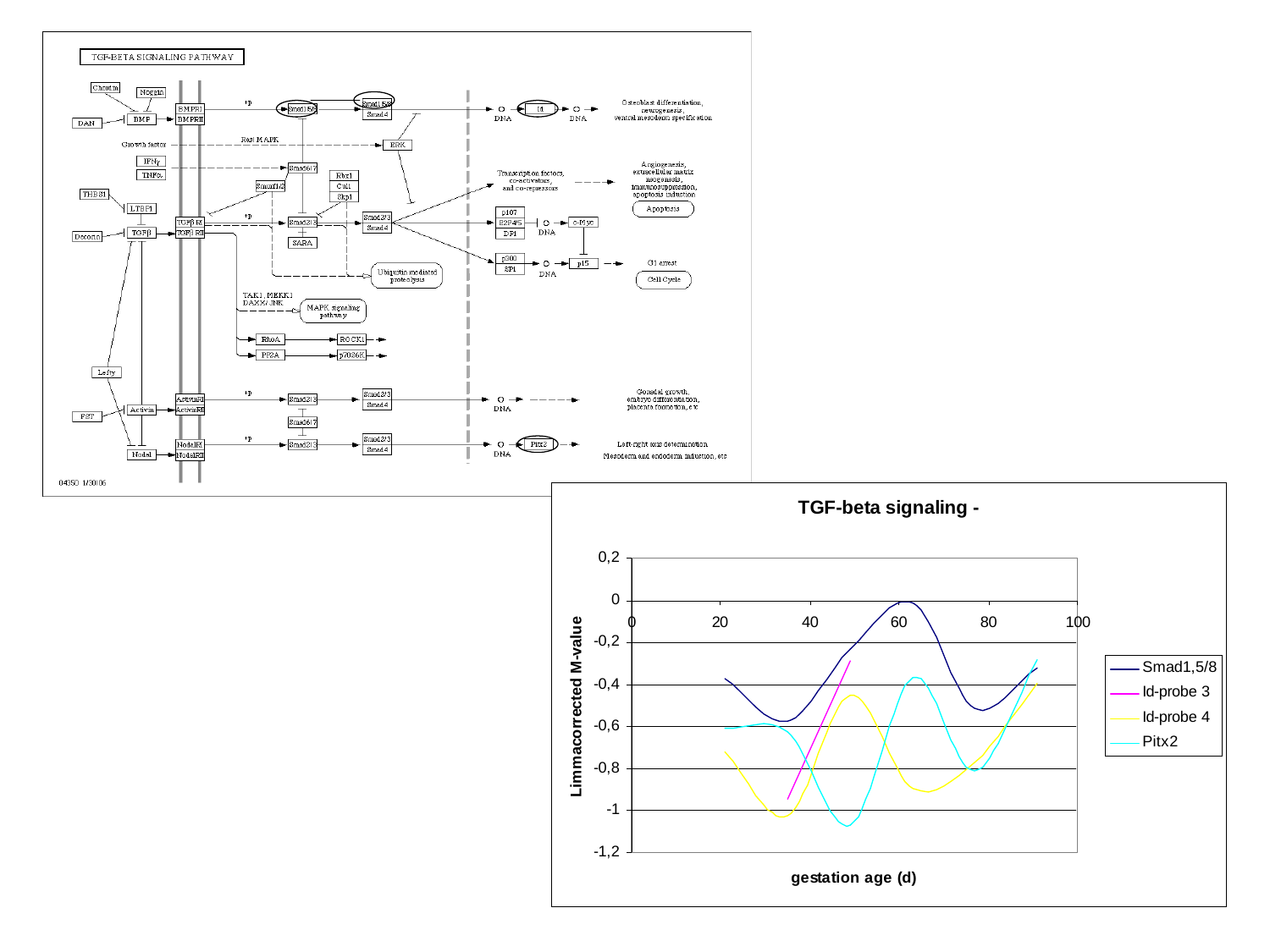

Supplement: Additional File 3 — Relevant KEGG pathways. Shows the pathways returned by KEGG with the genes with information on the microarrays indicated as circles around the gene name. Gene expression profiles are included [file 1471-213X-7-66-S3.ppt]
